# Expression of the sFLT1 Gene in Cord Blood Cells Is Associated to Maternal Arsenic Exposure and Decreased Birth Weight

**DOI:** 10.1371/journal.pone.0092677

**Published:** 2014-03-24

**Authors:** Sylvie Remy, Eva Govarts, Liesbeth Bruckers, Melissa Paulussen, Britt Wens, Elly Den Hond, Vera Nelen, Willy Baeyens, Nicolas van Larebeke, Ilse Loots, Isabelle Sioen, Greet Schoeters

**Affiliations:** 1 Environmental Risk and Health, Flemish Institute for Technological Research (VITO), Mol, Belgium; 2 Interuniversity Institute for Biostatistics and Statistical Bioinformatics, Hasselt University, Diepenbeek, Belgium; 3 Department of Biomedical Sciences, University of Antwerp, Antwerp, Belgium; 4 Department of Health, Provincial Institute for Hygiene, Antwerp, Belgium; 5 Department of Analytical, Environmental and Geochemistry (AEGC), Vrije Universiteit Brussel, Brussels, Belgium; 6 Department of Radiotherapy and Experimental cancer research, Ghent University, Ghent, Belgium; 7 Department Sociology, Faculty of Political and Social Sciences, University of Antwerp, Antwerp, Belgium; 8 Department of Public Health, Ghent University, Ghent, Belgium; 9 FWO Research Foundation, Brussels, Belgium; 10 Department of Environmental Medicine, University of Southern Denmark, Odense, Denmark; The University of Manchester, United Kingdom

## Abstract

There is increasing epidemiologic evidence that arsenic exposure in utero is associated with adverse pregnancy outcomes and may contribute to long-term health effects. These effects may occur at low environmental exposures but the underlying molecular mechanism is not clear. We collected cord blood samples of 183 newborns to identify associations between arsenic levels and birth anthropometric parameters in an area with very low arsenic exposure. Our core research aim was to screen for transcriptional marks that mechanistically explain these associations. Multiple regression analyses showed that birth weight decreased with 47 g (95% CI: 16–78 g) for an interquartile range increase of 0.99 μg/L arsenic. The model was adjusted for child’s sex, maternal smoking during pregnancy, gestational age, and parity. Higher arsenic concentrations and reduced birth weight were positively associated with changes in expression of the *sFLT1* (soluble fms-like tyrosine kinase-1) gene in cord blood cells in girls. The protein product of *sFLT1* is a scavenger of vascular endothelial growth factor (VEGF) in the extracellular environment and plays a key role in the inhibition of placental angiogenesis. In terms of fetal development, inhibition of placental angiogenesis leads to impaired nutrition and hence to growth retardation. Various genes related to DNA methylation and oxidative stress showed also changed expression in relation to arsenic exposure but were not related to birth outcome parameters. In conclusion, this study suggests that increased expression of *sFLT1* is an intermediate marker that points to placental angiogenesis as a pathway linking prenatal arsenic exposure to reduced birth weight.

## Introduction

Arsenic (As) is a naturally occurring semi-metallic element that can contaminate groundwater used for drinking and irrigation in areas where it’s abundant. In Asia, millions of people are chronically exposed to a high level of As in their drinking water from wells drilled into As-rich geologic strata [1]. Concerns are growing about inorganic As (iAs) in foods [2], especially in fruit juices and in rice even in areas with low levels of naturally occurring As. At high doses As increases risks of hyperkeratosis, pigmentation changes, cardiovascular diseases, hypertension, respiratory, neurological, liver and kidney disorders, as well as diabetes mellitus. As a carcinogen it is known to cause bladder, lung, and skin cancer (IARC, group I) [3]. iAs is metabolized by a series of methylation reactions, converting it to methylarsonic acid (MMA) and dimethylarsinic acid (DMA), which are excreted in urine [4]. Mainly inorganic As^III+^ and MMA, are reactive, have longer half-times in the tissues and considered more toxic.

iAs and its methylated metabolites pass the placenta. Concentrations measured in maternal blood correlate with concentrations in cord blood [5]. It has been suggested that transplacental transfer of As is driven by GLUT1 [6], which is known as the main transplacental glucose transporter [7]. The main site of GLUT1 receptors is the syncytiotrophoblast [8], with a greater degree of expression on the microvillous membrane compared to the basal membrane. Less abundantly, GLUT1 is expressed in cytotrophoblast cells and cells of the fetal vasculature [8]. When the intervillous space - that lines the microvillous membrane - is filled with maternal blood (2nd and 3th trimester of pregnancy), Arsenic present in maternal blood can easily pass to the fetal circulation by GLUT1 receptors. During the first 8–9 weeks of pregnancy, when the spiral arteries are blocked by plugs of invading trophoblast cells, maternal blood flow to the intervillous space is restricted [9]. As a result, the intervillous space is not yet filled with maternal blood but with a acellular fluid, derived from plasma and endometrial glands [10]. It is not clear whether As can already enter the intervillous space via plasma when the spiral arteries are blocked, however an increased odds of child’s later intellectual disability has been associated with specific exposure during this period [11].

Levels of As in breast milk are very low, even in areas with highly contaminated drinking water. In a study conducted in Bangladesh [12], the As concentration in urine of infants whose mothers reported exclusive breast-feeding ranged from 0.3 to 29 μg/L, whereas concentrations for those who reported partial breast-feeding ranged up to 1520 μg/L. Thus, exclusive breast-feeding protects the infant from exposure to As. The effects of As in breast-fed infants are probably more attributable to the intrauterine period of life as compared to the neonatal period, but further studies are necessary to confirm this.

There is a fairly consistent picture of *in utero* or early-life exposure to As at high levels in relation to elevated risk of adverse birth outcome in areas where As concentrations in drinking water that rise far above 40 μg/L (**Table S1**). However, epidemiological data reporting the effects at the very low exposure end, i.e. below the current WHO drinking water guidelines of 10 μg/L, are currently scarce. The most recent studies investigating low ranges of exposure reported that blood As levels were associated to reduced birth weight [13], [14] and birth length, and chest and head circumference [14]. This triggered us to study whether the reported associations could be confirmed in the Flemish population of Belgium, in which As concentrations were even 10 times lower as compared to both studies mentioned above. The majority of human cohort studies is conducted in areas with elevated As exposure and often lack individual exposure data and mechanistically effect data which could lead to understand a causal role for *in utero* As exposure in the development of later life disease.

Currently, the molecular mechanisms of action at higher As levels are partly understood. Toxicity pathways that have been suggested include DNA methylation [15]–[20], steroid receptor signaling [21]–[24], and oxidative stress [23], [25]–[29]. The mechanistic insights are derived mainly from *in vitro* studies and animal studies.

To the best of our knowledge, only one study exists in which gene expression had been investigated in relation to As exposure at the low-dose end, i.e. in New Hampshire (United States) [30]. The median As concentration in household tap water was 0.36 μg/L (IQR: 0.02–3.55). The expression of *AQP9* was identified as a potential placental biomarker for As exposure, while phospholipase *ENPP2* was positively associated with infant birth weight.

In the present paper we present the results of a cross sectional study of the association between birth outcomes and biomarkers of fetal exposure to As in a sample of mother–child pairs of the general population of Flanders. The As levels in the cohort are all very low. In addition, we measured gene expression of cord blood cells as an intermediate biomarker to screen biological pathways that may relate As exposure to the health outcome.

## Materials and Methods

### Ethics Statement

Written informed consent was provided by all mothers that participated in this study. The mothers also signed the informed consent on behalf of the children enrolled in the study. The study protocol was approved by the ethical committee of the University of Antwerp (Reference UA A08 09).

### Study Population

The study was a part of a biomonitoring program for environmental health surveillance in Flanders, Belgium [31]. Newborn-mother couples (n = 183) were recruited from the general population of Flanders between August 2008 and July 2009 using a multistage sampling procedure. This population consisted exclusively of uncomplicated liveborn singleton pregnancies. Ten maternities (two in each of the five Flemish provinces) - at least 20 km apart from each other - were randomly selected. All mothers that came to give birth were invited if they lived at least 10 years in Flanders and were able to fill in a Dutch questionnaire. The number of participants per province was proportional to the number of inhabitants in that province (status at 01/01/2006). The birth outcomes of interest (birth weight, head circumference at birth and gestational age) were obtained via the medical records of the maternities. Small for gestational age (SGA) was calculated as birth weight less than the 10^th^ percentile of birth weight for each week of pregnancy defined by externally obtained sex-specific reference weight curves 2001–2010 (Studiecentrum voor Perinatale Epidemiologie). Covariate/confounder data of individual health and lifestyle were obtained via questionnaires. The characteristics of the study population (N = 183) are presented in **Table S2**).

### Assessment of Arsenic Exposure

Total As exposure was measured in cord and maternal blood samples. Cord blood was collected immediately after birth, while maternal blood was collected during the mothers’ stay at the maternity unit of the hospital. Arsenic concentration in whole blood was measured by high resolution inductively coupled plasma-mass spectrometry (ICP-MS) [32]. The limit of detection (LOD) was 0.028 μg/L. For measurements below the LOD, we assigned a value of half the LOD for analysis. The Pearson correlation coefficient between the natural logarithm (Ln-transformed) of the As values in cord and maternal blood was determined. For the purpose of this study, statistical analysis was done with As measured in cord blood, as it will be linked to outcomes at birth, and As in maternal blood As was measured two days after delivery.

### Cord Blood Collection and Processing for Transcriptome Profiling

The gene expression pattern of blood cells were analysed with an Agilent microarray platform. Aliquots of cord blood (2–3 mL) were collected in Tempus Blood Tubes (Applied Biosystems) as quickly as possible after delivery. After collection, the content of the tubes was mixed by inverting the tubes. Samples were kept at 4°C at the hospital (max 48 hours) and frozen at −20°C upon arrival in our lab.

### RNA Processing and Quantification

Total RNA was extracted from the blood samples using the Tempus Spin RNA isolation kit (Applied Biosystems) according to manufacturer’s instructions. RNA yields (13–160 μg/sample) were measured using the NanoDrop Spectrophotometer (NanoDrop Technologies). The integrity of the RNA was tested with capillary gel-electrophoresis using RNA 6000 Chips (Agilent Technologies) analyzed on the Agilent 2100 Bioanalyzer. RIN values ranged from 7 to 9.6. Samples were stored at −80°C.

### RNA Amplification and Labeling

Total RNA was amplified and labelled to generate complementary RNA (cRNA) labelled with cyanine (Cy)3-CTP using the Low Input Quick Amp Labelling (one color) kit (Agilent Technologies) according to the manufacturer’s instructions. The single-stranded, labelled cRNA was purified with Qiagen’s RNeasy mini spin columns (Qiagen). Yield and specific activity (dye-incorporation rate) were determined using the NanoDrop spectrophotometer.

### Microarray Hybridisation and Data Preprocessing

For each sample, 1.65 μg cRNA was hybridized per microarray (4×44 K Agilent Whole Human Genome, design 014850) during 17 h using the automated HS4800TM pro hybridization station (Tecan), according to the manufacturer’s instructions. The arrays were scanned with an Agilent scanner (G2565BA). The arrays were subjected to a primary quality control using the Agilent Feature Extraction Software (Version 10.7). For each feature on the array, the software accurately determines the intensity (gProcessedSignal, signal intensity derived from Cy3 fluorescent dye), which were used as input for further processing and analysis (Matlab, version R2012b). Normalization between arrays was performed by means of quantile normalization upon logarithmic transformation of the data (log_2_-scale). For sequences that are replicated on the array, mean(log_2_(gProcessedSignal)) was calculated. Feature Extraction files include quality control parameters for each of the sequences to assess reliability of the data. A reliable sequence is defined as a sequence 1) of which the intensity is higher than the feature noise level, 2) of which the intensity is below the feature saturation level, 3) that is not a population outlier, 4) of which the spot is uniform, and 5) of which the background is not an outlier. This information was taken into account in the analysis as described in section ‘Statistical analysis’.

### RNA Processing and qPCR

For a subset of 30 samples RNA was reverse transcribed to cDNA for qPCR analysis using a Roche LightCycler480. Prior to cDNA synthesis, RNA samples were subjected to DNase treatment (DNA-free™ Kit, Invitrogen). cDNA synthesis and qPCR reactions were performed as described previously [33]. PrimeTime™ assays obtained from Integrated DNA Technologies were used (Leuven, Belgium). Details of the 5′ exonuclease PrimeTime™ assays that were used are given in **Table S3**. qPCR data processing was performed using the LightCycler software [33]. Stability of the reference genes (GNB2L1, RPLP0, RPL13A) was verified using geNorm [34]. Gene expression changes were analyzed using the qBase software [35]. Gene expression changes were calculated as fold changes (FC) relative to the expression level in one of the samples that was chosen as reference. Subsequently, FCs were log_2_-transformed to enable comparison with microarray data.

### Statistical Analysis

For the identification of associations between As exposure and adverse pregnancy outcome, database management and statistical analyses were performed with SAS software version 9.3 (SAS Institute Inc., Cary, NC, USA). Linear and logistic regression analyses were used to quantify the effect on different growth parameters associated with an interquartile range (IQR) increase of the exposure to As. The exposure-response relations were adjusted for *a priori* fixed known confounders, gender of the newborn and smoking of the mother during pregnancy, that were included irrespective of their significance level. Other influencing factors (covariates) were included when significant at the 0.05 level in the multiple regression model. The following covariates were considered: gestational age (only for birth weight and head circumference), maternal age, parity, stress/pressure during pregnancy, maternal education level, (passive) smoking before pregnancy, alcohol use before/during pregnancy, maternal height, maternal body mass index (BMI), equivalent income, infections/complication during pregnancy, use of folic acid during pregnancy and caesarean section (only for gestational age). Effect modification (interaction) was analyzed in models including main effects and cross-product terms, and by doing stratified analyses. A p-value <0.20 for the effect of the cross-product was taken as an indication of interaction. Assumptions of normality, constancy of variance, independence (randomness), and linearity were checked with informal diagnostic plots and formal tests (White’s General test for constancy of variance [36], Kolmogorov-Smirnov test for normality, and the lack of fit test for linearity [37]). Influence statistics (residuals, leverage) were used to detect outliers and influential data points, and regression models were fitted with and without these influential outliers.

For the statistical analysis of the transcriptome data Matlab was used, version 2012b. For our analysis we focused on a subset of genes that were associated with intrauterine growth, DNA methylation, oxidative stress, and As-related genes (**Table S4**). Genes related to the first 3 processes were retrieved from the Ingenuity Knowledge Base (Ingenuity Systems, www.ingenuity.com), the list of As- related genes was obtained from a recent publication [30]. We evaluated whether changes in the expression of these molecules in cord blood cells can be related to 1) As exposure, and/or 2) prenatal growth. For both comparisons we evaluated on the subset of most extreme samples in the dataset. With regards to As exposure, we tested whether ‘high’ exposed samples show differences in expression as compared to ‘low’ exposed samples by independent two-sample t-test (two-sided, unequal variance) for boys and girls separately. ‘High’ means As level in cord blood higher than geometric mean plus standard deviation (above 1.81: μg/L, n_boys_ = 14, n_girls_ = 17), and ‘low’ smaller than geometric mean minus standard deviation (below 0.17 μg/L: n_boys_ = 12, n_girls_ = 19). P-value below 0.05 was used as a cut-off for statistical significance. We aimed to follow a similar approach to select ‘high’ and ‘low’ birth weight babies. For this, adjusting birth weight a priori for gestational age was necessary to categorize the samples. A linear regression model - including a quadratic term - was fitted separately for male en female babies with gestational age as the independent variable and birth weight as the outcome variable. For each individual, the ratio between observed birth weight and the estimated birth weight at the same gestational age was calculated. Following Ln-transformation of this ratio, ‘high’ and ‘low’ birth weight samples were identified. ‘High birth weight’ means Ln-transformed ratio above mean plus standard deviation (n_boys_ = 14, n_girls_ = 10), and ‘low birth weight’ smaller than mean minus standard deviation (n_boys_ = 15, n_girls_ = 14). Analogue to As exposure, we tested whether ‘low birth weight’ samples show differences in expression as compared to ‘high birth weight’ samples for boys and girls separately. Following a targeted approach (focused on a subset of genes among the whole array), we decided to not correct for multiple testing. Transcripts for which the microarray data were not reliable for all samples being considered (according to the criteria mentioned previously) were excluded from statistical analysis.

**Figure 1:**
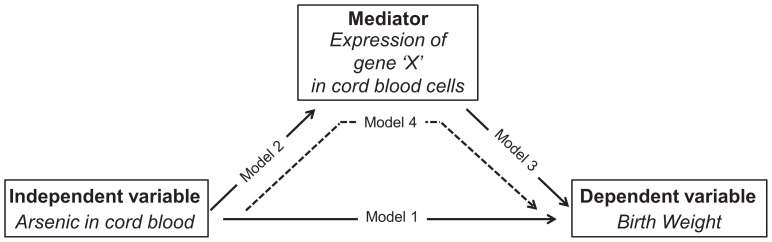
Mediation analysis. Microarray analysis was used to identify gene expression markers that are associated with both As exposure and birth weight. Subsequently, a stepwise approach [38] was followed to investigate the mediating effects of the candidate gene expression markers in the association between As exposure and birth weight by linear regression models. The method is described for hypothetical marker gene ‘X’. To demonstrate mediation, four requirements must be met: Model 1) Birth weight (dependent variable) should be associated with As exposure (independent variable); model 2) The expression of gene ‘X’ (mediator) should be associated with As exposure; model 3) Birth weight should be associated with the expression of gene ‘X’; and model 4) the expression of gene ‘X’ should be a significant predictor of birth weight, while controlling for As exposure. The estimated change in birth weight related to As exposure in model 4 should be less compared to model 1 to demonstrate partial mediation, and drop to zero to demonstrate full mediation.

To demonstrate mediating effects of gene expression in the association between As exposure and birth weight, a mediation analysis was performed by the procedure described by Baron and Kenny (1986) [38]. This method was applied to the markers identified in microarray analysis (previous paragraph) that may potentially link As exposure to birth weight. Applied to hypothetical marker gene ‘X’ in mediating the association between As and birth weight, a stepwise approach was followed in which the 4 requirements of mediation [38] were verified by linear regression (**Figure 1**). In the regression models, As exposure was categorized in two groups, a group of ‘high’ exposure (level in cord blood above geomean plus standard deviation, n = 31) and a group of ‘median or low’ exposure (the remaining samples, n = 152). For birth weight, the continuous measure (expressed as gram birth weight) was used. The following covariates were considered: gender, gestational age, smoking during pregnancy, and parity. Effect modification (interaction) was analyzed in models including main effects and cross-product terms. To meet the four requirements of mediation, birth weight (dependent variable) should be associated with As exposure (independent variable) (*Model 1*). Secondly, As exposure should be a significant predictor of the expression of gene ‘X’ (the mediator) in cord blood cells. In addition, the expression of gene ‘X’ should be a significant predictor of birth weight (model 3). In the final step (model 4), it must be confirmed that the expression of gene ‘X’ is a significant predictor of birth weight, while controlling for As exposure. The estimated change in birth weight related to As exposure (β_As_) in model 4 should be less compared to model 1 to demonstrate mediation. Maximum evidence for mediation (full mediation) occurs if by inclusion of the expression of gene ‘X’, β_As_ drops to zero. In partial mediation β_As_ in model 4 is weaker, yet still significant, compared to model 1. In that case, gene expression of gene ‘X’ accounts for some – but not all – of the observed association between As exposure and birth weight. The following covariates were considered: gender, gestational age, smoking during pregnancy, and parity. Effect modification (interaction) was analyzed in models including main effects and cross-product terms.

For confirmation of the expression of *sFLT1* as measured with microarray by use of qPCR, the correlation between both measures was calculated for transcript NM_001159920. Input values obtained from 30 samples were log_2_-transformed fold changes relative to one of the samples (which was randomly selected). Analogue, correlation between qPCR data obtained from NM_001159920 and NM_001160030 (two transcript variants of sFLT1 was calculated). For NM_001159920, the difference in expression among ‘high’ and ‘low’ As exposure was analyzed by independent two-sample t-test (two-sided, unequal variance) and compared with differential expression measured by microarray.

## Results

### Arsenic Exposure in Relation to Birth Outcome Parameters

**Figure 2:**
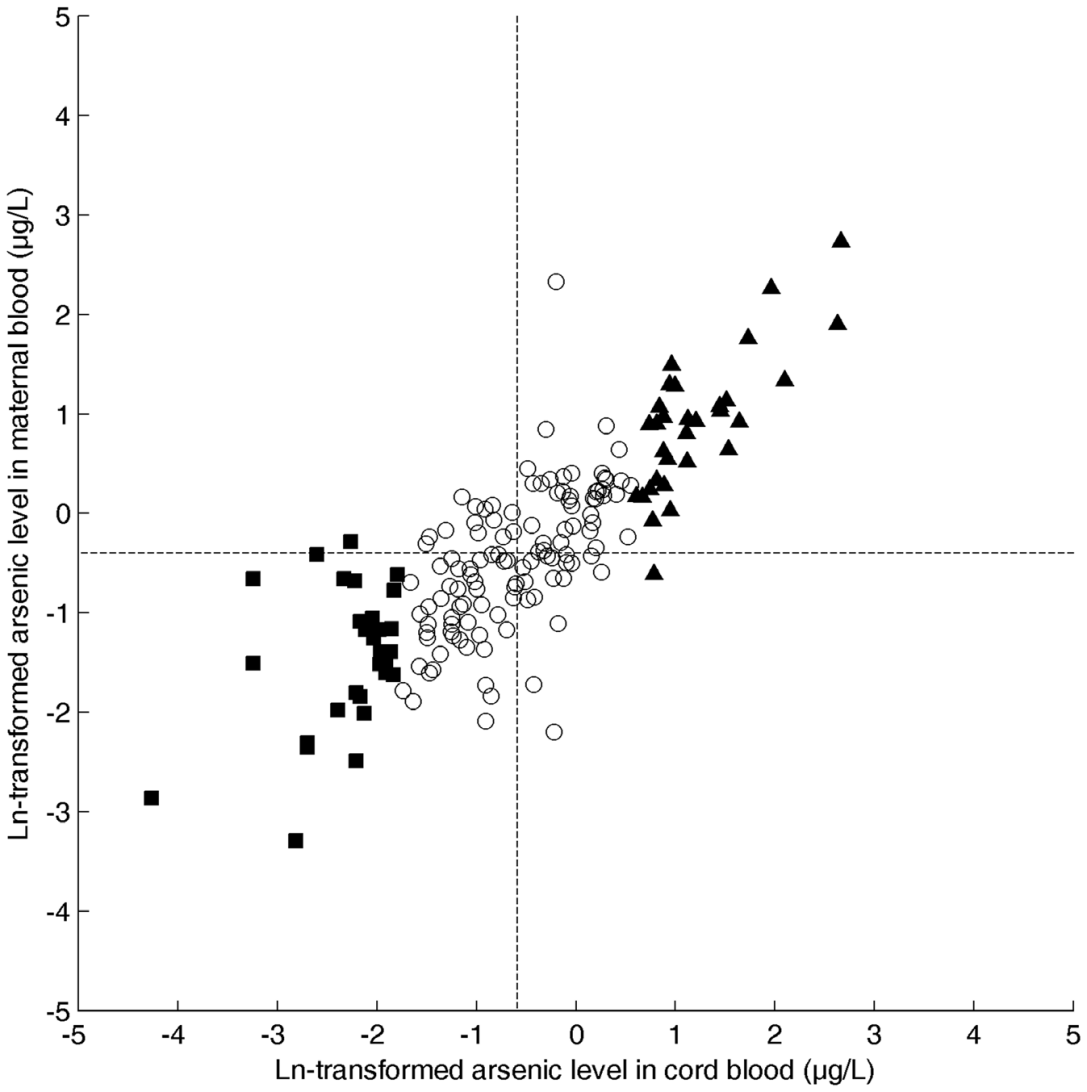
Correlation between the level of arsenic measured in cord blood and maternal blood. The Pearson correlation coefficient r between the natural logarithm (ln-transformed) of the As values in cord blood (horizontal axis) and maternal blood (vertical axis) equals 0.82 (N = 177, p-value <0.0001). The vertical and horizontal dotted line correspond to the geometric mean of cord blood and maternal blood As levels respectively. For downstream analysis, the level of As in cord blood was categorized, i.e. ‘low As’ (filled squares): samples for which As level smaller than geometric mean minus standard deviation; ‘high As’ (filled triangles): higher than geometric mean plus standard deviation; ‘median As’ (open circles): all samples in between.

The geometric mean value of As in cord blood was 0.56 μg/L (95% CI: 0.47–0.66 μg/L). The As levels ranged up to 14.4 μg/L, with one value below the limit of detection (LOD) of 0.028 μg/L (replaced by LOD/2), and an interquartile range of 0.99 μg/L. As levels in maternal and cord blood were highly correlated (Pearson correlation coefficient for Ln-transformed values r = 0.82 (p<0.0001)). The correlation between both measures is visualized by **Figure 2**.

Fourteen of the newborns (7.7%) were born as SGA-babies, the gestational age ranged from 34 to 42 weeks with a median of 40 weeks (**Table S2**).

The multiple regression models showed a significant association (p<0.05) between increasing As concentrations in cord blood and an increased risk of SGA and lower birth weight. For an increase of As cord blood levels with the interquartile range ( = 0.99 μg/L), the risk for SGA babies increased with 38% (95% CI: 11–71%) and birth weight decreased with 47 g (95% CI: 16–78 g) (**Table 1**). The models were adjusted for child’s sex, smoking of the mother during pregnancy, gestational age (only for birth weight), and parity (only for birth weight). The model assumptions were fulfilled, and the associations stayed statistically significant after fitting the model without the influential outliers. No statistically significant associations were found between As levels in cord blood and head circumference at birth and gestational age.

**Table 1:** Multiple logistic (SGA) and linear (birth weight) regression analyses.

Birth outcome	N	Explanatory variable	Unit	Estimate^a^ (95% CI)	p-value
SGA	178	Cord blood As	↑0.99 μg/L	1.38 (1.11, 1.71)	0.0037
		Sex of newborn	boys vs. girls	1.13 (0.32, 3.97)	0.8448
		Smoking during pregnancy	yes vs no	2.91 (0.67, 12.71)	0.1562
Birth weight	177	Cord blood As	↑0.99 μg/L	−47 (−78, −16)	0.0033
		Sex of newborn	boys vs. girls	161 (45, 277)	0.0069
		Smoking during pregnancy	yes vs no	−111 (–288, 66)	0.2185
		Gestational age	↑ 1 week	216 (172, 261)	<0.0001
		Parity	0 vs. 2+	−184 (−327, −42)	0.0115
			1 vs. 2+	−30 (−179, 120)	0.6942
			0 vs. 1	−155 (−292, −17)	0.028

N = number of subjects; CI = Confidence Interval; SGA = Small for Gestational Age; As = Arsenic; ↑ = increase with interquartile range; vs. = versus.

a Interpretation of the estimate: for SGA: the odds of having SGA is multiplied with the estimate ( = the odds ratio); for birth weight: the estimate is the increase/decrease in weight.

The associations were not modified by sex when looking at the cross-product term of sex with As levels (p-values ranged from 0.63–0.90). When doing stratified analyses, the associations between As and birth outcomes were still statistically significant in boys, but not in girls both for SGA and birth weight. Both estimates were however in the same order, but due to power (only about 90 individuals in each model) not statistically significant in girls.

### Expression of Genes Associated to ‘Embryonal Growth’

**Figure 3:**
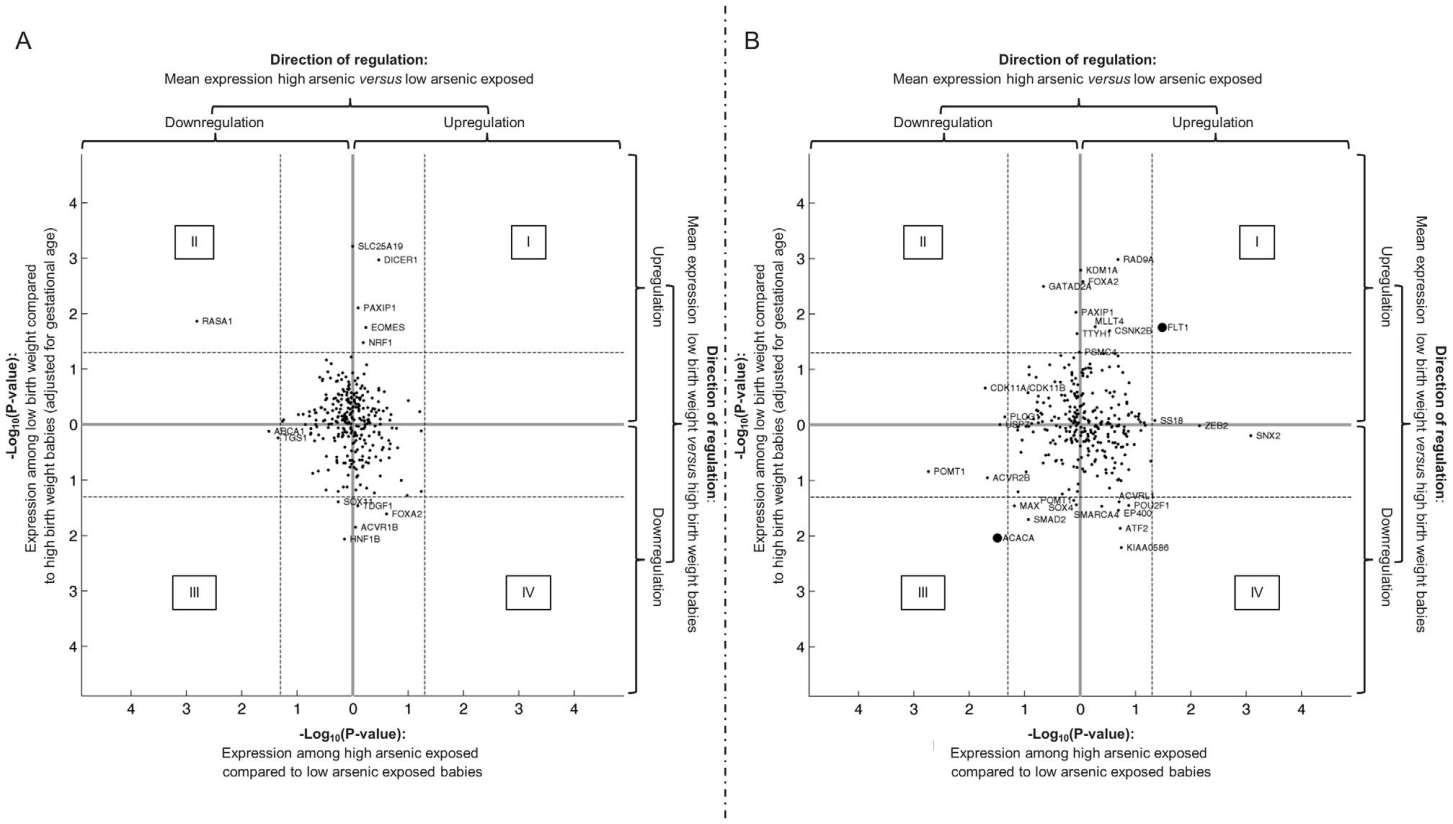
Genes associated to ‘embryonal growth’. This figure shows the potential of genes related to embryonal growth (N = 156) to link the level of As exposure to decreased birth weight for boys (A) and girls (B) separately. For each of the sequences, the significance (−log10(PValue)) of differential regulation between ‘high’ and ‘low’ As is plotted on the horizontal axis, and between ‘low’ and ‘high’ birth weight is plotted on the vertical axis. The direction of regulation (up or down) is included in the plot. The 1^st^ quadrant is composed of transcripts that show higher expression among the group of high As and the group of low birth weight as compared to the group of low As and the group of high birth weight, respectively. Making the same comparisons, transcripts that show lower expression can be found in the 3^rd^ quadrant. The dashed threshold lines represent p-value equal to 0.05. For transcripts of which the p-value is smaller than 0.05 in either one of the comparisons, the corresponding gene symbol is included in the plot.

To identify molecules that might link the level of As exposure to elevated risk on SGA, cord blood gene expression was analyzed. After microarray quality control filtering, 158 different genes could be annotated to the function ‘embryonal growth’ according the Ingenuity Knowledge Base (**Table S4**). **Figure 3** demonstrates the potential of the transcripts to 1) discriminate the group of ‘high’ As exposure from the group of ‘low’ As exposure, and 2) discriminate the group of ’low’ birth weight from ‘high’ birth weight (adjusted for gestational age). The analysis was performed for boys (**Figure 3A**) and girls (**Figure 3B**) separately. For each of the sequences, the significance (−log10(PValue)) of differential regulation between ‘high’ and ‘low’ As is plotted on the horizontal axis, and between ‘low’ and ‘high’ birth weight is plotted on the vertical axis. Using quadrant visualisation, this plot also takes into account the direction of regulation. The 1^st^ quadrant is composed of transcripts that show higher expression among the group of high As and the group of SGA as compared to the group of low As and the group of LGA, respectively. Making the same comparisons, transcripts that show lower expression can be found in the 3^rd^ quadrant. Aiming to identify molecules that might link the level of As exposure to elevated risk on SGA these quadrants are of special interest, since the other 2 contain genes that show opposite regulation of transcription in ‘high’ versus ‘low’ As exposure, as compared to ‘low’ versus ‘high’ birth weight. The corresponding gene symbol of transcripts with p-value smaller than 0.05 (dashed threshold lines) in either ‘high’ versus ‘low’ As exposure or ‘low’ versus ‘high’ birth weight is included on the plot. Among girls, two transcripts show stronger potential to link the level of As exposure to elevated risk on SGA. One of them is upregulated in ‘high’ As exposed and ‘low’ birth weight samples, i.e. a soluble transcript variant of *FLT1* (NM_001159920). The corresponding p-value for differential expression between ‘high’ and ‘low’ As exposure equals 0.033, and 0.017 between ‘low’ and ‘high’ birth weight. The other transcript (*ACACA*, Acetyl-CoA Carboxylase Alpha) is downregulated in expression in ‘high’ As exposed and ‘low’ birth weight samples. The corresponding p-value for differential expression between ‘high’ and ‘low’ As exposure equals 0.032, and 0.009 between ‘low’ and ‘high’ birth weight. For boys, no transcripts could be identified that show strong potential to link the level of As exposure to elevated risk on SGA.

**Figure 4:**
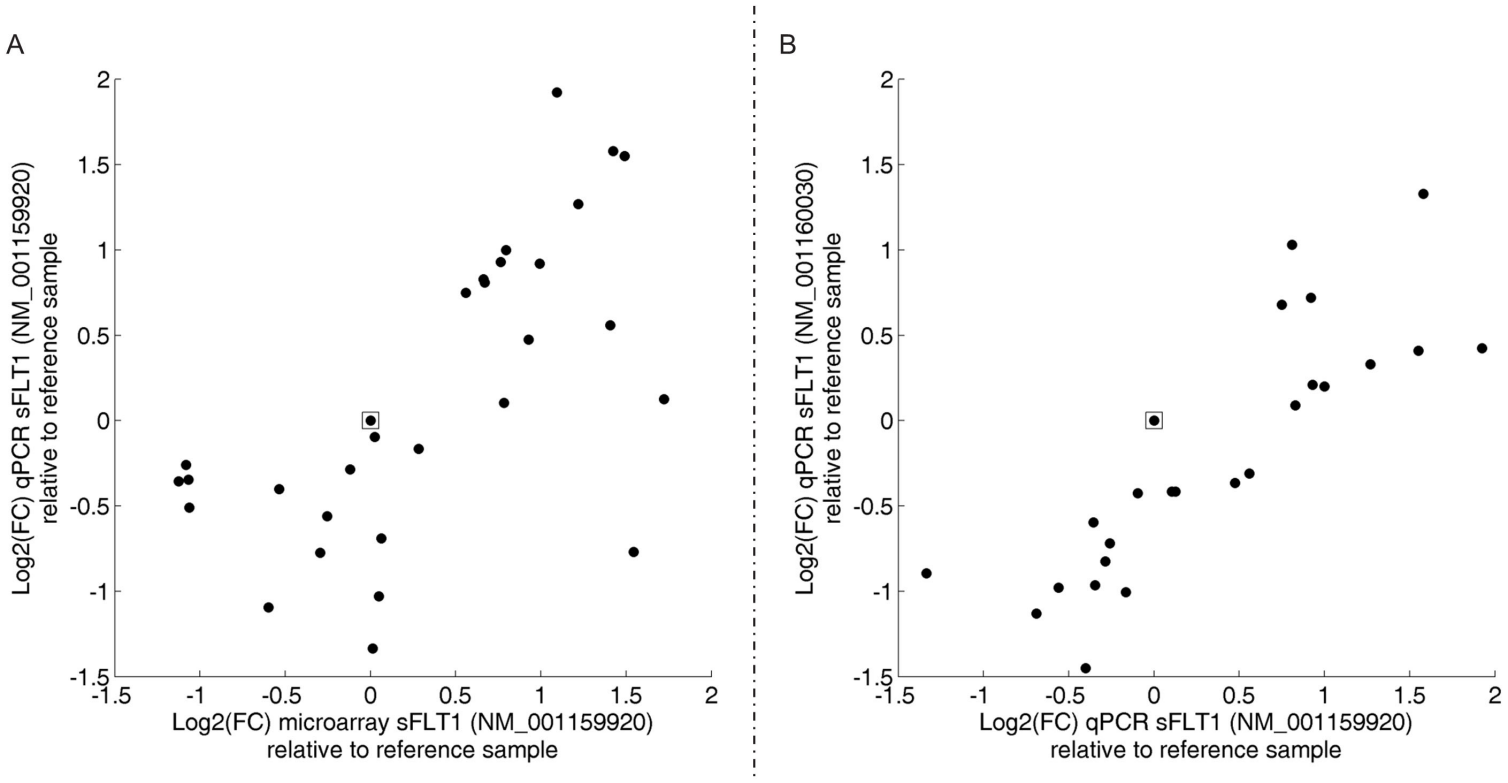
qPCR analysis of sFLT1. A) Confirmation of gene expression of sFLT1 (NM_001159920) as measured by microarray with by qPCR for a subset of 30 samples. For each sample, the expression of *sFLT1* (was calculated as ratio to one given sample (reference sample, open square) and subsequently logarithmically transformed (log2 scale). The Pearson correlation coefficient equals = 0.64 (p = 1.33E−04). B) Correlation between two transcript variants of sFLT1 (NM_001159920 and NM_001160030, N = 30). The pearson correlation coefficient equals 0.87 (p-value = 2.55E−04).

The expression of *FLT1* was also measured by qPCR in a subset of 30 samples. Different transcript variants of *FLT1* exist, coding for either the soluble isoform (*sFLT1*, NM_001159920, NM_001160030, NM_001160031) or the transmembrane isoform (*mFLT1*, i.e. NM_002019) of the protein. The data obtained for the *mFLT1* did not successfully pass quality control (lower expression range, near detection limit). For *sFLT1*, reported microarray data are restricted to NM_001159920 as the sequence spotted on the microarray is specific for that variant. Use of qPCR enabled us to measure two different variants of *sFLT1*, variant NM_001160031 was not detectable. Microarray and qPCR data of NM_001159920 correlated very well (Pearson correlation coefficient r = 0.64, p = 1.33E−04). The correlation between both measures is visualized by **Figure 4A**. The subset of 30 samples that was measured by qPCR included 15 samples of ‘high’ As exposure and 15 samples of ‘low’ As exposure. The samples were selected such that discrimination in expression as measured by microarray was highly significant (P-value ‘high’ vs ‘low’ as measured by qPCR was 8.2291E−10). Measuring the expression by qPCR, the difference between the two groups remained highly significant (P-value = 3.2857E−07). In addition, correlation between qPCR data obtained for NM_001159920 and NM_001160030 was assessed (**Figure 4B**, Pearson correlation coefficient r = 0.87, p = 2.55E−04).

### Expression of Genes Associated to ‘DNA Methylation’

Analogue to the approach described in the previous paragraph, genes involved in DNA methylation were analyzed (**Figure S1**). The list of genes retrieved from the Ingenuity Knowledge Base can be found in **Table S4**. The expression of *MYC* was positively associated to the As level in boys (**Figure S1A**). In girls (**Figure S1B**), respectively *ARID4A* and *PLD6* were significantly up- and downregulated, with increasing As levels. However, none of these genes could be linked to decreased birth weight.

### Expression of Genes Previously Associated with as Exposure

To enable comparison with a recent publication [30], we also focused on the expression of a panel of 9 As-related genes (*AKR1C3, ENPP2, HMOX1, LEP, NFE2L2, TYMS, AQP9, AS3MT,* and *SLC39A2*). They all successfully passed quality control filtering (**Table S4**). None of them was found to be significantly different (p-value <0.05) in expression among ‘high’ versus ‘low’ exposure or among ‘low’ versus ‘high’ birth weight.

### Expression of Genes Associated to Oxidative Stress

Several studies indicate that oxidative stress may play a role in As toxicity. A list of genes that are known to be involved in oxidative stress responses was retrieved from the Ingenuity Knowledge Base (**Table S4**). A volcano plot that includes p-value and fold change (in ‘high As’ *versus* ‘low As’) for all 152 genes for boys and girls separately can be found in **Figure S2**. Among boys (**Figure S2A**), the expression of four genes differed between ‘high’ and ‘low’ As (*GPX7, SNCA, YBX1,* and *MYC*). In girls (**Figure S2B**), *BRCA1, MMP2, MMP9, PEMT, S100A12, SELK, TAT, and VNN1* were differentially expressed. The direction of regulation can be derived from the plot.

### Mediating Effects of Gene Expression in the Association between Arsenic Exposure and Birth Weight

Two genes showed changes in expression in response to As exposure that could also be linked to reduced birth weight, i.e. *sFLT1* and *ACACA*. To explore to what extent the expression of these genes mediates the association between As exposure and birth weight, we verified whether the requirements to demonstrate mediation were met according to Baron and Kenny, 1986 [38] (**Table 2**). The association between As exposure and birth weight which we demonstrated in table 1 remained significant after categorizing the exposure level (**Table 2**, model 1), while adjusting for the same set of parameters (gender, gestational age, smoking during pregnancy, and parity). The interaction between As exposure and gender was not significant (p = 0.21) and therefore not included in the regression model. Birth weight among ‘high’ exposed babies was 236 g lower (95% CI: −397; −79 g, p = 0.0035) compared to low or median exposed babies. Subsequently, we evaluated the expression of *sFLT1* and *ACACA* in function of As level, while adjusting for gender and gestational age. Increased *sFLT1* expression was significantly associated with As exposure (p = 0.0196, **Table 2**, model 2). The interaction between gender and As exposure was also evaluated, but was not significant and therefore not considered. The log2 transformed fold change of high exposed relative to low or median exposed babies was equal to 0.30 (95% CI: 0.05; 0.55). Considering the expression of *ACACA*, the significant difference that was demonstrated for girls between ‘high’ and ‘low’ exposure could not be reproduced by analyzing ‘high’ versus ‘low or median’ exposed (p = 0.3891). Including the interaction between gender and As exposure did not improve the result. Due to this, we limited the proceeding steps in mediation analysis and the statistical output in table 2 to *sFLT1.* In the next step, we evaluated birth weight in function of *sFLT1*, while adjusting for gender, gestational age, smoking during pregnancy, and parity (**Table 2**, model 3). The interaction between *sFLT1* expression and gender was significant (p = 0.0450) and therefore also included. A significant reduction in birth weight and *sFLT1* expression was found for girls (p = 0.0061), but not for boys (p = 0.9775). Duplication in *sFLT1* expression in girls was associated with a reduction in birth weight of 182 g (95% CI: −311; −52 g). In the final model (**Table 2**, model 4), we demonstrate that *sFLT1* was a significant predictor of birth weight among the population of girls (p = 0.0118), while controlling for As exposure. The reduction in birth weight associated with ‘high’ As exposure was less pronounced (−219 g, 95% CI: −376; −62 g) as compared to the first model (−236 g, 95% CI: −394; −79 g) in which the effect of gene expression was not included. This indicates that sFLT1 may be considered as a (partial) mediator in the association between As exposure and reduced birth weight.

**Table 2:** *sFLT1* gene expression as a mediator in the association between arsenic exposure and birth weight.

Model	N	Dependent variable	Predictor variable		β	95% CI	p-value	p-value <0.05
**Model 1**	177	Birth Weight (g)	Arsenic (high vs. median or low)		−236	−394; −79	0.0035	*
**Model 2**	183	sFLT1 expression	Arsenic (high vs. median or low)		0.30	0.05; 0.55	0.0196	*
**Model 3**	177	Birth Weight (g)	Interaction sFLT1:Gender				0.0450	*
			sFLT1 (expression level)	Boys	−2	−123; 119	0.9775	
				Girls	−182	−311; −52	0.0061	*
**Model 4**	177	Birth Weight (g)	Interaction sFLT1:Gender				0.0429	*
			sFLT1 (expression level)	Boys	14	−105; 133	0.8175	
				Girls	−164	−292; −37	0.0118	*
			Arsenic (high vs. median or low)		−219	−376; −62	0.0066	*

N = number of subjects; CI = Confidence Interval; SGA = Small for Gestational Age; g = gram;

* = p-value <0.05; High arsenic = Arsenic level in cord blood above geomean plus standard deviation (N = 31); Median or low arsenic: arsenic level in cord blood below geomean plus standard deviation (N = 152).

Interpretation of the estimate (β): **Model 1)** Difference in birth weight between high arsenic exposed and low to median exposed newborns; **Model 2)** Log2(fold change) in sFLT1 expression between high arsenic exposed and low to median exposed newborns; **Model 3)** Difference in birth weight for a duplication in sFLT1 expression; **Model 4)** For sFLT1: Difference in birth weight for a duplication in sFLT1 expression - For arsenic: Difference in birth weight between high arsenic exposed and low to median exposed newborns.

The associations in model 1 were adjusted for gender, gestational age, smoking during pregnancy, and parity; The associations in model 2 were adjusted for gender and gestational age; The associations in model 3 and 4 were adjusted for gender, gestational age, smoking during pregnancy, parity, and the interaction between gender and sFLT1.

As a sensitivity analysis, it has been verified whether the associations reported were influenced by maternal BMI and pregnancy complications. Including BMI in each of the linear regression models shown in **table 2** did not influence the significant findings that are reported. The fraction of the population that reported pregnancy complications (i.e. high blood pressure, pre-eclampsia, or gestational diabetes during pregnancy) was very small (N = 8, table S2), therefore the associations reported were not adjusted for these complications. It has been verified whether the significant associations reported in the mediation analysis (**Table 2**) were maintained 1) after adjusting for pregnancy complications; and 2) when the participants reporting one of these complications were excluded. By inclusion of this variable in the models, the associations reported remained significant. By exclusion of the participants reporting complications, the significant associations were also maintained.

## Discussion

This study provides evidence that As exposure even at low environmental levels is associated with birth anthropometric parameters. Decrease in birth weight, independent of gestational age, and an increased risk for babies born SGA was related to increasing As concentrations in cord blood samples of newborn babies. The association was observed at an exposure range which is applicable to the exposure of ‘control subjects’ in other studies (**Table S1**). Although maternal urinary As concentrations were not analysed in our cohort, we have data of urinary levels of total As and toxicologically relevant As (the sum of inorganic As, monomethylarsonic acid (MMA), and dimethylarsinic acid (DMA)) in a cohort from 101 women aged between 20 and 40 sampled in the same campaign. In this population, which is representative for Flanders, the median levels of total As and toxicological relevant As were respectively 16.2 μg/L (25^th^ percentile: 6.9 μg/L; 75^th^ percentile: 30.1 μg/L) and 4.3 μg/L (25^th^ percentile: 2.9 μg/L; 75^th^ percentile: 7.7 μg/L). The observed effect on birth weight confirms findings at relatively low levels [13], [14]. However, the median As level in our cord blood samples was 0.53 μg/L (25^th^ percentile: 0.2 μg/L; 75^th^ percentile: 1.2 μg/L) and is even about 10 times lower compared to both studies mentioned. No significant associations were found between As levels in cord blood and head circumference at birth and gestational age. The latter could be an indication that the associations between As and SGA and birth weight were not mediated by effects on gestational age.

We tested whether changes in gene expression in cord blood cells could reflect molecular links between As exposure and birth outcomes. Specific gene panels were selected that were previously already associated with intrauterine growth retardation, As exposure, or with potential mode of actions of As such as oxidative stress and DNA methylation. Of these, two genes showed changes in expression in response to As exposure that could also be linked to the risk of SGA in our birth cohort, i.e. *sFLT1* and *ACACA*. Based on mediation analysis, *sFLT1* is the most potential gene expression marker to link As exposure to reduced birth weight. The negative association between As exposure and birth weight; and also the positive association between As and *sFLT1* was gender independent (as the interaction term between gender and As was not significant). However, the effect of differential expression of *sFLT1* on birth weight was only significant in girls, while there was absence of evidence for an association between *sFLT1* and birth weight for boys.

*sFlt1* is a natural soluble factor, it is the truncated version of VEGFR1 (or *mFLT1*) lacking transmembrane and intracellular signaling domains. *sFLT1 is* a key molecule in inhibition of placental angiogenesis, it inhibits angiogenesis by binding to free forms of VEGF and PlGF [39]. In terms of fetal development, inhibition of placental angiogenesis leads to impaired nutrition and hence to growth retardation [40]. In a review by Tseng et al., chronic high level exposure to As has already been linked to increased rates of pre-eclampsia [41]. In humans sFLT1 is used as a biomarker for pre-eclampsia, but increased placental transcript levels have also been demonstrated in normotensive pregnancy with a SGA fetus [42]. This may indicate a common pathway involved in the development of both conditions. The major source of excess circulating sFLT1 protein in pre-eclampsia is the placenta [39]. In 2005, Rajakumar et al. showed that peripheral blood mononuclear cells comprise an extra-placental source of sFLT1 that could potentially contribute to the pre-eclampsia disease process [43]. Makris et al. investigated the relationship between uteroplacental ischemia (UPI, an important trigger of pre-eclampsia) and sFLT1 in non-human primates. They demonstrated that induction of ischemia in the placenta also affects *sFLT1* mRNA expression in circulating cells not exposed directly to ischemia [44]. Although the change in *sFLT1* expression was less than the rise in placental expression, it indicates that – at least for *sFLT1* - blood cells mRNA reflect transcriptional changes in the placenta.

Disturbed regulation of placental angiogenesis confirms in humans what has been demonstrated earlier in animal experiments. Sodium arsenite caused spontaneous abortion in mice via aberrant placental vasculogenesis and placental insufficiency [45]. In a broader framework, i.e. the cardiovascular system, As has been shown to lead to cardiovascular diseases [46], including hypertension [47], carotid atherosclerosis [48], and ischemic heart disease [49] in non-pregnant residents from highly contaminated areas. These vascular effects have been attributed to reduced NO levels, which acts as a potent vasodilator. *In vitro* studies using human umbilical vein endothelial cells confirmed that inorganic arsenite or arsenate (100 μM) significantly suppressed the activity of human eNOS [50]. It has been described that sFLT1 plays a key role in regulation of eNOS [51]. As an angiogenic inhibitor, an elevation in sFLT1 supresses eNOS expression. Considering the low exposure range in our population, it is reasonable to hypothesize that early molecular signs (sFLT1) of a cascade that eventually leads to disturbance of the vascular system are present, while downstream markers (such as eNOS) remain unchanged.

Although gender dichotomy in the mechanism of growth restriction driven by sFLT1 has not been reported before, a very recent study demonstrated sexual dimorphism in placental function in severe pre-eclampsia [52]. In summary, they showed that in pre-eclamptic pregnancies the placentas of males were associated with significantly higher expression of inflammatory, hypoxia and apoptotic molecules but reduced expression of a pro-angiogenic marker compared to placentas of female fetuses. In a mouse model of pre-eclampsia induced by sFLT1, significantly higher blood pressure was demonstrated in male offspring that were born to sFLT1–treated mothers as compared to control mice [53]. The effect was not present in female offspring. Reduced uterine perfusion in pregnant rats - that results in intrauterine growth restricted (IUGR) offspring - has also been associated with hypertension [54]. Male IUGR offspring remained hypertensive in adulthood, whereas female IUGR offspring developed marked elevations in mean arterial pressure prior to puberty but normalize their blood pressure after puberty to levels comparable to female control offspring. Ojeda et al. demonstrated that testosterone contributes to the elevations in mean arterial pressure in adult male IUGR offspring [55]. In studies that report gender dichotomy in the context of pre-eclampsia or IUGR, the effects are generally more pronounced in males. This is different from our findings in which the association between birth weight and *sFLT1* expression was restricted to female neonates. However, it suggests that hormonal status may play a role in phenotypic modulation. In the context of our results, it can be hypothesized that co-regulatory gender dependent signals are involved in directing the sFLT1-pathway towards its specific outcome. Accumulating data suggest that As may act as an endocrine disruptor. It has been demonstrated that monomethylated trivalent As species inhibit binding of glucocorticoid (GR) and progesterone receptor (PR) with their DNA response elements that are crucial to hormone-driven gene transcription [21].

Our results indicate that *sFLT1* is a partial mediator – and not a full mediator - of the association between As exposure and birth weight. This means that expression of *sFLT1*, accounts for some – but not all – of the observed association. It implies that additional factors are involved that could not be identified in our study. Gene expression in cord blood was considered as a surrogate marker to reflect adverse reactions in the tissues targeted by As exposure. However, it cannot be assumed that gene expression measurements in cord blood always reflect gene expression changes within other tissues. This implies that potential mediators may be missed. In addition, levels of regulation that were not covered in our study – such as post transcriptional and epigenetic regulations – may provide additional mediators to explain the association between As exposure and reduced birth weight.

## Conclusion

This study provides new insight in low-dose prenatal As toxicity. Results of the 2^nd^ Flemish Environment and Health Study showed that the concentration of As in cord blood of newborns in the present study was linked with reduced birth weight and a higher risk of having a SGA baby, even though exposure in Flanders is low compared to other regions. Impaired fetal growth is a risk factor to develop metabolic problems later in life. Molecular data indicate that impaired placental vascularisation, by upregulation of *sFLT1*, might be a mechanistic basis that mediates the association between As exposure and reduced birth weight.

## Supporting Information

Figure S1 Genes associated to ‘DNA methylation’. This figure shows the potential of genes related to DNA methylation to link the level of As exposure to decreased birth weight for boys (A) and girls (B) separately. For each of the sequences, the significance (−log10(PValue)) of differential regulation between ‘high’ and ‘low’ As is plotted on the horizontal axis, and between ‘low’ and ‘high’ birth weight is plotted on the vertical axis. The direction of regulation (up or down) is included in the plot. The dashed threshold lines represent p-value equal to 0.05. For transcripts of which the p-value is smaller than 0.05 in either one of the comparisons, the corresponding gene symbol is included in the plot.
(TIFF)

Figure S2 Genes associated to ‘Oxidative stress’. The volcano plot includes the p-value and fold change (in ‘high Arsenic’ *versus* ‘low Arsenic’) of genes related to oxidative stress (N = 152) for boys (A) and girls (B) separately. The dashed treshold lines correspond to p-value equal to 0.05. For transcripts of which the p-value is smaller than 0.05, the corresponding gene symbol is included in the plot.
(TIFF)

Table S1 Literature overview of adverse pregnancy outcomes in relation to As exposure. Abbreviations: CI: Confidence interval; OR = Odds ratio; P10 = 10^th^ percentile; P25 = 25^th^ percentile; P75 = 75^th^ percentile; P90 = 90^th^ percentile; SGA: small for gestational age 1. Ahmad SA, Sayed MH, Barua S, Khan MH, Faruquee MH, et al. (2001) Arsenic in drinking water and pregnancy outcomes. Environ Health Perspect 109∶629-631. 2. Guan H, Piao F, Zhang X, Li X, Li Q, et al. (2012) Prenatal exposure to arsenic and its effects on fetal development in the general population of Dalian. Biol Trace Elem Res 149∶10-15. 3. Hopenhayn C, Ferreccio C, Browning SR, Huang B, Peralta C, Gibb H, Hertz-Picciotto I (2003) Arsenic exposure from drinking water and birth weight. Epidemiology 14∶593-602. 4. Hopenhayn-Rich C, Browning SR, Hertz-Picciotto I, Ferreccio C, Peralta C, et al. (2000) Chronic arsenic exposure and risk of infant mortality in two areas of Chile. Environ Health Perspect 108∶667-673. 5. Huyck KL, Kile ML, Mahiuddin G, Quamruzzaman Q, Rahman M, et al. (2007) Maternal arsenic exposure associated with low birth weight in Bangladesh. J Occup Environ Med 49∶1097-1104. 6. Milton AH, Smith W, Rahman B, Hasan Z, Kulsum U, et al. (2005) Chronic arsenic exposure and adverse pregnancy outcomes in bangladesh. Epidemiology 16∶82-86. 7. Rahman A, Vahter M, Ekstrom EC, Rahman M, Golam Mustafa AH, et al. (2007) Association of arsenic exposure during pregnancy with fetal loss and infant death: a cohort study in Bangladesh. Am J Epidemiol 165∶1389-1396. 8. Rahman A, Vahter M, Smith AH, Nermell B, Yunus M, et al. (2009) Arsenic exposure during pregnancy and size at birth: a prospective cohort study in Bangladesh. Am J Epidemiol 169∶304-312. 9. Rahman A, Persson LA, Nermell B, El AS, Ekstrom EC, et al. (2010) Arsenic exposure and risk of spontaneous abortion, stillbirth, and infant mortality. Epidemiology 21∶797-804. 10. von Ehrenstein OS, Guha Mazumder DN, Hira-Smith M, Ghosh N, Yuan Y et al. (2006) Pregnancy outcomes, infant mortality, and arsenic in drinking water in West Bengal, India. Am J Epidemiol 163∶662-669. 11. Xu L, Yokoyama K, Tian Y, Piao FY, Kitamura F, et al. (2011) Decrease in birth weight and gestational age by arsenic among the newborn in Shanghai, China. Nihon Koshu Eisei Zasshi 58∶89-95. 12. Yang CY, Chang CC, Tsai SS, Chuang HY, Ho CK, et al. (2003) Arsenic in drinking water and adverse pregnancy outcome in an arseniasis-endemic area in northeastern Taiwan. Environ Res 91∶29-34.
(DOCX)

Table S2 Descriptive statistics of the study population (183 mother-newborn pairs). Abbreviations: N = number of subjects; P25 = 25^th^ percentile; P75 = 75^th^ percentile; SGA = Small for Gestational Age.
(DOCX)

Table S3 Sequences of 5′ exonuclease PrimeTime™ assays that were used for qPCR analysis.
(DOCX)

Table S4 Subset of genes that were associated with intrauterine growth, DNA methylation, oxidative stress, and As-related genes. Quality control: +: Quality control passed for given sequence, −: Quality control not passed for given sequence. Embryonal growth: Number of sequences Agilent array = 294 (160 unique genes); Number of sequences Agilent array after quality control filtering = 289 (158 unique genes). DNA methylation: Number of sequences Agilent array = 102 (49 unique genes); Number of sequences Agilent array after quality control filtering = 101 (48 unique genes). Oxidative stress: Number of sequences Agilent array = 275 (154 unique genes); Number of sequences Agilent array after quality control filtering = 269 (152 unique genes).
(DOCX)
