# Supplementary figures and images for: Expression of the sFLT1 Gene in Cord Blood Cells Is Associated to Maternal Arsenic Exposure and Decreased Birth Weight
Source: PLoS One. 2014 Mar 24;9(3):e92677. doi: 10.1371/journal.pone.0092677 (PMC3963915; doi:10.1371/journal.pone.0092677)

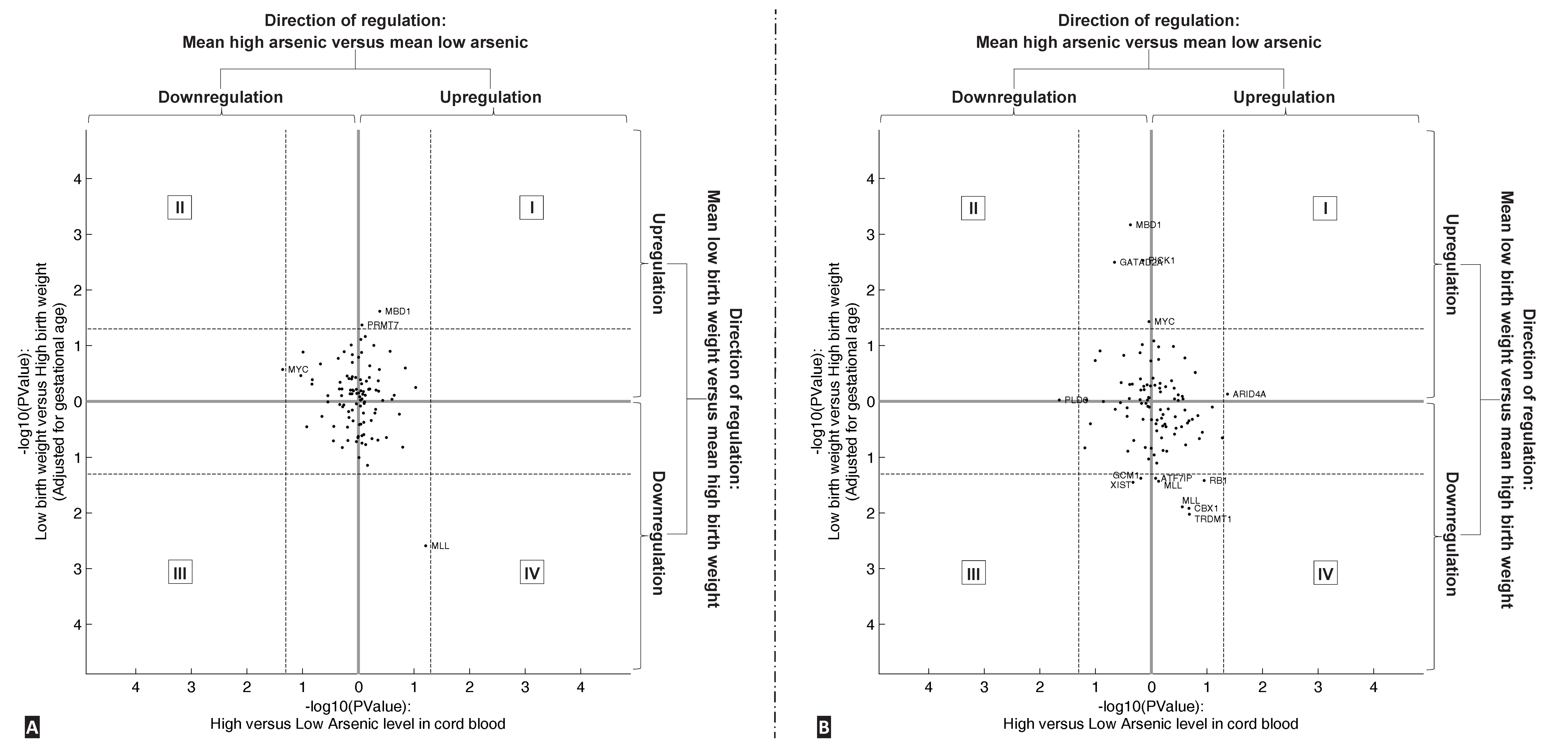

Supplement: Figure S1 — Genes associated to ‘DNA methylation’. This figure shows the potential of genes related to DNA methylation to link the level of As exposure to decreased birth weight for boys (A) and girls (B) separately. For each of the sequences, the significance (−log10(PValue)) of differential regulation between ‘high’ and ‘low’ As is plotted on the horizontal axis, and between ‘low’ and ‘high’ birth weight is plotted on the vertical axis. The direction of regulation (up or down) is included in the plot. The dashed threshold lines represent p-value equal to 0.05. For transcripts of which the p-value is smaller than 0.05 in either one of the comparisons, the corresponding gene symbol is included in the plot. (TIFF) [file pone.0092677.s001.tif]

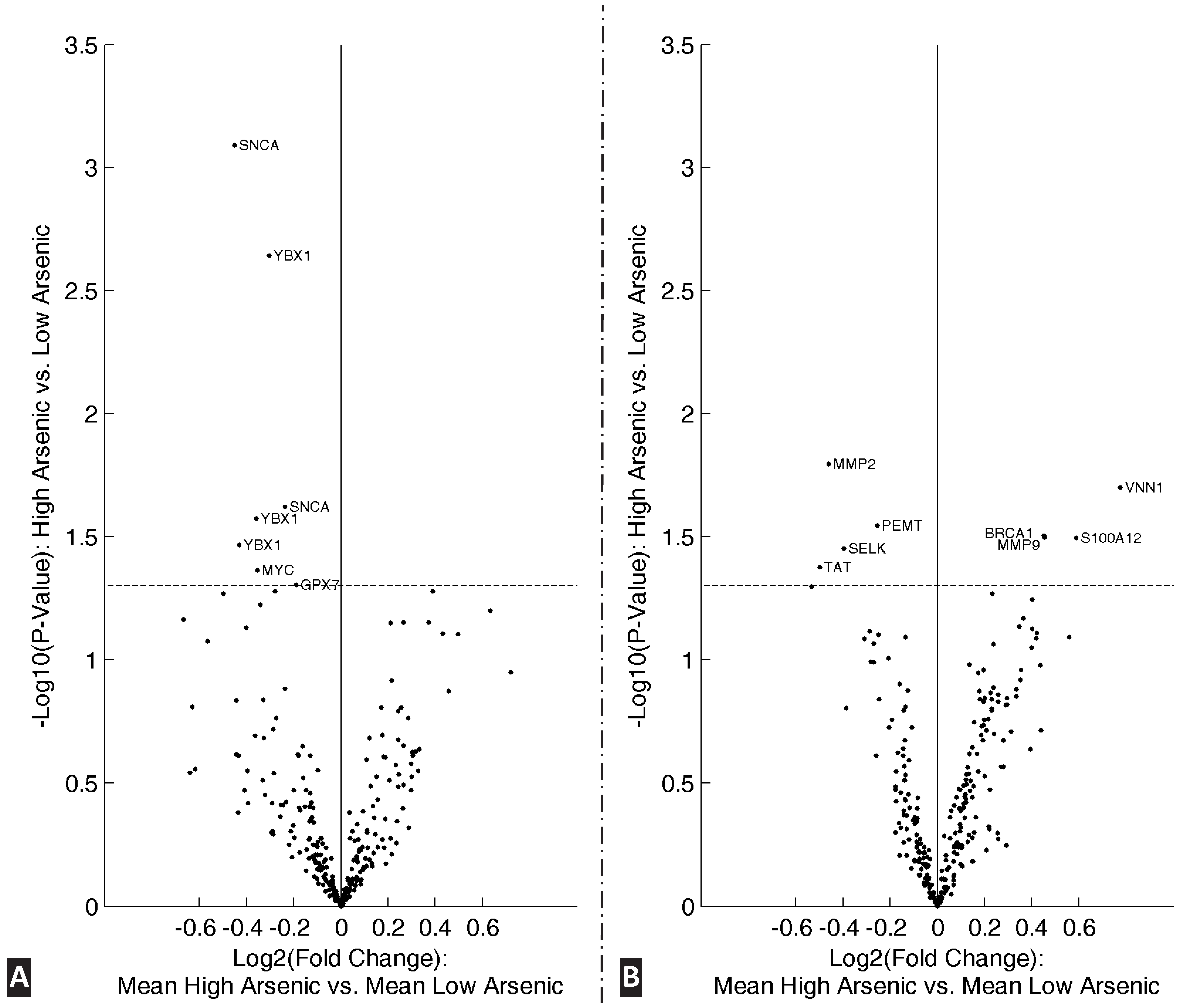

Supplement: Figure S2 — Genes associated to ‘Oxidative stress’. The volcano plot includes the p-value and fold change (in ‘high Arsenic’ versus ‘low Arsenic’) of genes related to oxidative stress (N = 152) for boys (A) and girls (B) separately. The dashed treshold lines correspond to p-value equal to 0.05. For transcripts of which the p-value is smaller than 0.05, the corresponding gene symbol is included in the plot. (TIFF) [file pone.0092677.s002.tif]
