# Supplementary material for: Expression of the sFLT1 Gene in Cord Blood Cells Is Associated to Maternal Arsenic Exposure and Decreased Birth Weight
Source: PLoS One. 2014 Mar 24;9(3):e92677. doi: 10.1371/journal.pone.0092677 (PMC3963915; doi:10.1371/journal.pone.0092677)
Supplement: Table S1 — Literature overview of adverse pregnancy outcomes in relation to As exposure. Abbreviations: CI: Confidence interval; OR = Odds ratio; P10 = 10th percentile; P25 = 25th percentile; P75 = 75th percentile; P90 = 90th percentile; SGA: small for gestational age 1. Ahmad SA, Sayed MH, Barua S, Khan MH, Faruquee MH, et al. (2001) Arsenic in drinking water and pregnancy outcomes. Environ Health Perspect 109∶629-631. 2. Guan H, Piao F, Zhang X, Li X, Li Q, et al. (2012) Prenatal exposure to arsenic and its effects on fetal development in the general population of Dalian. Biol Trace Elem Res 149∶10-15. 3. Hopenhayn C, Ferreccio C, Browning SR, Huang B, Peralta C, Gibb H, Hertz-Picciotto I (2003) Arsenic exposure from drinking water and birth weight. Epidemiology 14∶593-602. 4. Hopenhayn-Rich C, Browning SR, Hertz-Picciotto I, Ferreccio C, Peralta C, et al. (2000) Chronic arsenic exposure and risk of infant mortality in two areas of Chile. Environ Health Perspect 108∶667-673. 5. Huyck KL, Kile ML, Mahiuddin G, Quamruzzaman Q, Rahman M, et al. (2007) Maternal arsenic exposure associated with low birth weight in Bangladesh. J Occup Environ Med 49∶1097-1104. 6. Milton AH, Smith W, Rahman B, Hasan Z, Kulsum U, et al. (2005) Chronic arsenic exposure and adverse pregnancy outcomes in bangladesh. Epidemiology 16∶82-86. 7. Rahman A, Vahter M, Ekstrom EC, Rahman M, Golam Mustafa AH, et al. (2007) Association of arsenic exposure during pregnancy with fetal loss and infant death: a cohort study in Bangladesh. Am J Epidemiol 165∶1389-1396. 8. Rahman A, Vahter M, Smith AH, Nermell B, Yunus M, et al. (2009) Arsenic exposure during pregnancy and size at birth: a prospective cohort study in Bangladesh. Am J Epidemiol 169∶304-312. 9. Rahman A, Persson LA, Nermell B, El AS, Ekstrom EC, et al. (2010) Arsenic exposure and risk of spontaneous abortion, stillbirth, and infant mortality. Epidemiology 21∶797-804. 10. von Ehrenstein OS, Guha Mazumder DN, Hira-Smith M, Ghosh N, Yuan Y et al. (2006) Pr [file pone.0092677.s003.docx]

**Table S1.** Literature overview of adverse pregnancy outcomes in relation to As exposure

| **First Author, Year** | **Region** | **Arsenic: Exposure assessment** | **Adverse pregnancy outcome** |
| --- | --- | --- | --- |
| Our study | Flanders, Belgium | **Maternal blood:** Median = 0.7 µg/L, P25 = 0.3µg/L, P75 = 1.3 µg/L; | Increase of cord blood arsenic concentration by IQR (0.99 µg/L): |
|  |  | **Cord blood:** Median = 0.5 µg/L, P25 = 0.2 µg/L, P75 = 1.2 µg/L; | - Increased risk of SGA (52%, 95% CI: 21 - 91%); |
|  |  | **Drinking water standard Flanders:** < 10 µg/L | - Decrease in birth weight (45g, 95% CI: 15 - 75 g); |
| Ahmad, 2001 [1] | Bangladesh: Sampta (exposed group); | **Drinking water:** >= 100 µg/L (exposed) *versus < 20 µg/L* (nonexposed) | Exposed versus nonexposed group, significantly higher rates of: |
|  | Katiarchar (non-exposed group) |  | - Spontaneous abortion (p = 0.008); |
|  |  |  | - Stillbirth (p = 0.046); |
|  |  |  | - Preterm birth (p = 0.018) |
| Guan, 2012 [2] | China: Dalian | **Maternal blood:** Median = 5.3 µg/L; | Maternal arsenic concentration in blood negatively associated with: |
|  |  | **Cord blood:** Median = 3.7 | - Birth weight and height |
|  |  |  | - Chest circumference; |
|  |  |  | Cord blood arsenic concentration negatively associated with: |
|  |  |  | - Head circumference |
| Hopenhayn, 2003 [3] | Chile: Antofagasta (contaminated) and | **Drinking water:** 40 µg/L *versus* < 1 µg/L ; | Exposure to 40 µg/L of arsenic in drinking water: |
|  | Valparaíso (low exposure) | **Urine:** 54.3 µg/L (SD: 33.8) *versus* 5.3 µg/L (SD 3.3) | - Reduction in birth weight (–57 g; 95% CI: –123 to 9) |
| Hopenhayn-Rich, 2000 [4] | Chile: Antofagasta (contaminated) | **Drinking water:** Historical contamination Antofagasta, | Significant association between arsenic exposure and: |
|  | and Valparaíso (low exposure) | peak 1958-1970: 860 µg/L | - Late fetal mortality (RR = 1.7, 95% CI = 1.5-1.9); |
|  |  |  | - Neonatal mortality (RR = 1.53, 95% CI: 1.4 - 1.7); |
|  |  |  | - Postneonatal mortality (RR = 1.26, 95% CI = 1.2 - 1.3) |
| Huyck, 2007 [5] | Bangladesh: Sirajdikhan Upazila | **Drinking water:** < 1.0–734.0 µg/L; | Maternal hair arsenic measured early in pregnancy associated with: |
|  |  | **Maternal hair:** 0.14 - 3.28 µg/g; | - Decreased birth weight (β = -193.5 ± 90g) |
|  |  | **Maternal nail:** 0.19 – 6.15 µg/g; |  |
|  |  | **Newborn hair:** < 0.001 – 0.78 µg/g; |  |
|  |  | **Newborn nail:** 0.14–2.63 µg/g |  |
| Milton, 2005 [6] | Bangladesh: Chandpur district and | **Drinking water:** below detection limit to 1710 µg/L | Exposure > 50µg/L versus expsosure <= 50µg/L: |
|  | Chuadanga district |  | - Spontaneous abortion (OR = 2.5, 95% CI: 1.5 - 4.3); |
|  |  |  | - Stillbirth (OR = 2.5, 95% CI: 1.3 - 4.9); |
|  |  |  | - Neonatal death (OR = 1.8, 95% CI: 0.9 - 3.6) |
| Rahman, 2007 [7] | Bangladesh: Matlab | **Drinking water:** Mean: 239 µg/L; | Exposure > 50µg/L: |
|  |  | Median: 224 µg/L; P10 < 1 µg/L; P90: 513 µg/L | - Fetal Loss (Relative Risk = 1.14, 95% CI: 1.04 - 1.25); |
|  |  |  | - Infant death (Relative Risk: 1.17, 95% CI: 1.03 - 1.32) |
| Rahman, 2009 [8] | Bangladesh: Matlab | **Urine:** Median = 95 µg/L, P10 = 26 µg/L, P90 = 444 µg/L | Lower exposure level (0 - 100µg/L), increase of 1µg/L associated to: |
|  |  |  | - Reduction in birth weight with 1.68 g; |
|  |  |  | - Reduction in head circumference with 0.05 mm; |
|  |  |  | - Reduction in chest circumference with 0.14 mm. |
|  |  |  | With higher exposure level (> 100µg/L), no additional effect was observed. |
| Rahman, 2010 [9] | Bangladesh: Matlab | **Urine:** Median: 382 μg/L; P20: < 33 µg/L; P80: 249 -1253 µg/L | Exposure P80 versus P20: |
|  |  |  | - Spontaneous abortion: OR = 1.4, 95% CI: 0.96 - 2.2; |
| Rahman, 2010 [9] | Bangladesh: Matlab | **Urine:** Median: 390 μg/L; P20: < 38 µg/L; P80: 268-2019 µg/L | Exposure P80 versus P20: |
|  |  |  | - Infant mortality: Hazard ratio = 5.0, CI: 1.4 - 1.8 |
| von Ehrenstein, 2006 [10] | West Bengal, India | **Drinking water:** Categorical: 0-49 µg/L; 50-199 µg/L; >= 200 µg/L | Exposure to >= 200 µg/L: |
|  |  |  | - Increased risk of stillbirth (OR = 6.07, 95% CI: 1.54 to 24.0); |
|  |  |  | - Increased risk of neonatal death (OR = 2.81, 95% CI: 0.73 - 10.8) |
| Xu, 2011 [11] | China: Shangai | **Maternal blood**: Mean = 4,13 µg/L; | Maternal arsenic concentration in blood negatively associated to: |
|  |  | **Cord blood**: Mean = 3,82 µg/L | - Birth weight; |
|  |  |  | - Gestational age |
| Yang, 2003 [12] | Taiwan: 4 townships in the northeast | **Drinking water:** Exposed group: Up to 3590 µg/L, | Exposed *versus* control group: |
|  | (exposed group) versus 4 townships | highest median concentration of 4 townships = 140 µg/L; | - Reduction in birth weigth (25.09 g, 95%CI: 13.55 - 44.55) |
|  | with no historic evidence of arsenic | Control group: < 0,9 µg/L |  |
|  | water contamination (control group) |  |  |
|  |  |  |  |

Abbreviations:

CI: Confidence interval; OR = Odds ratio; P10 = 10^th^ percentile; P25 = 25^th^ percentile; P75 = 75^th^ percentile; P90 = 90^th^ percentile; SGA: small for gestational age

Reference List:

1. Ahmad SA, Sayed MH, Barua S, Khan MH, Faruquee MH, et al. (2001) Arsenic in drinking water and pregnancy outcomes. Environ Health Perspect 109: 629-631.

2. Guan H, Piao F, Zhang X, Li X, Li Q, et al. (2012) Prenatal exposure to arsenic and its effects on fetal development in the general population of Dalian. Biol Trace Elem Res 149: 10-15.

3. Hopenhayn C, Ferreccio C, Browning SR, Huang B, Peralta C, Gibb H, Hertz-Picciotto I (2003) Arsenic exposure from drinking water and birth weight. Epidemiology 14: 593-602.

4. Hopenhayn-Rich C, Browning SR, Hertz-Picciotto I, Ferreccio C, Peralta C, et al. (2000) Chronic arsenic exposure and risk of infant mortality in two areas of Chile. Environ Health Perspect 108: 667-673.

5. Huyck KL, Kile ML, Mahiuddin G, Quamruzzaman Q, Rahman M, et al. (2007) Maternal arsenic exposure associated with low birth weight in Bangladesh. J Occup Environ Med 49: 1097-1104.

6. Milton AH, Smith W, Rahman B, Hasan Z, Kulsum U, et al. (2005) Chronic arsenic exposure and adverse pregnancy outcomes in bangladesh. Epidemiology 16: 82-86.

7. Rahman A, Vahter M, Ekstrom EC, Rahman M, Golam Mustafa AH, et al. (2007) Association of arsenic exposure during pregnancy with fetal loss and infant death: a cohort study in Bangladesh. Am J Epidemiol 165: 1389-1396.

8. Rahman A, Vahter M, Smith AH, Nermell B, Yunus M, et al. (2009) Arsenic exposure during pregnancy and size at birth: a prospective cohort study in Bangladesh. Am J Epidemiol 169: 304-312.

9. Rahman A, Persson LA, Nermell B, El AS, Ekstrom EC, et al. (2010) Arsenic exposure and risk of spontaneous abortion, stillbirth, and infant mortality. Epidemiology 21: 797-804.

10. von Ehrenstein OS, Guha Mazumder DN, Hira-Smith M, Ghosh N, Yuan Y et al. (2006) Pregnancy outcomes, infant mortality, and arsenic in drinking water in West Bengal, India. Am J Epidemiol 163: 662-669.

11. Xu L, Yokoyama K, Tian Y, Piao FY, Kitamura F, et al. (2011) Decrease in birth weight and gestational age by arsenic among the newborn in Shanghai, China. Nihon Koshu Eisei Zasshi 58: 89-95.

12. Yang CY, Chang CC, Tsai SS, Chuang HY, Ho CK, et al. (2003) Arsenic in drinking water and adverse pregnancy outcome in an arseniasis-endemic area in northeastern Taiwan. Environ Res 91: 29-34.
