# Supplementary material for: Expression of the sFLT1 Gene in Cord Blood Cells Is Associated to Maternal Arsenic Exposure and Decreased Birth Weight
Source: PLoS One. 2014 Mar 24;9(3):e92677. doi: 10.1371/journal.pone.0092677 (PMC3963915; doi:10.1371/journal.pone.0092677)
Supplement: Table S2 — Descriptive statistics of the study population (183 mother-newborn pairs). Abbreviations: N = number of subjects; P25 = 25th percentile; P75 = 75th percentile; SGA = Small for Gestational Age. (DOCX) [file pone.0092677.s004.docx]

**Table S2.** Descriptive statistics of the study population (183 mother-newborn pairs)

| **Mothers** |  |  |  |
| --- | --- | --- | --- |
|  | **N** | **median (P25-P75)** | **min.-max.** |
| Gestational age (weeks) | 183 | 40 (39-40) | 34-42 |
| Age (years) | 183 | 30 (27-33) | 18-42 |
| Height (cm) | 181 | 168 (163-171) | 148-183 |
| Pre-pregnancy weight (kg) | 182 | 62 (57-69) | 42-117 |
|  |  |  |  |
|  | **N** | **categories** | **N (%)** |
| Pre-pregnancy body mass index (kg/m²) | 181 | < 18.5 | 12 (6.6%) |
|  |  | [18.5 , 25 [ | 129 (71.3%) |
|  |  | [25 , 30 [ | 27 (14.9%) |
|  |  | >= 30 | 13 (7.2%) |
| Educational level | 181 | Lower secondary | 18 (9.9%) |
|  |  | Higher secondary | 55 (30.4%) |
|  |  | Higher education | 108 (59.7%) |
| Parity | 182 | 0 | 70 (38.5%) |
|  |  | 1 | 61 (33.5%) |
|  |  | 2+ | 51 (28.0%) |
| Smoking before pregnancy | 179 | Never smoked | 94 (52.5%) |
|  |  | Ex smoker | 27 (15.1%) |
|  |  | Less than daily smoker | 11 (6.2%) |
|  |  | Daily smoker | 47 (26.3%) |
| Smoking during pregnancy | 178 | Yes | 23 (12.9%) |
|  |  | No | 155 (87.1%) |
| Passive smoking before pregnancy | 112 | Yes | 66 (58.9%) |
|  |  | No | 46 (41.1%) |
| Alcohol before pregnancy | 183 | Never | 27 (14.8%) |
|  |  | Less than monthly | 46 (25.1%) |
|  |  | Less than weekly | 38 (20.8%) |
|  |  | Weekly | 72 (39.3%) |
| Alcohol during pregnancy | 182 | Yes | 74 (40.7%) |
|  |  | No | 108 (59.3%) |
| Stress during pregnancy | 182 | Never-sometimes | 130 (71.4%) |
|  |  | Mostly-always | 52 (28.6%) |
| High Workload | 182 | Never-sometimes | 94 (51.7%) |
|  |  | Mostly-always | 88 (48.4%) |
| Infections during pregnancy | 180 | Yes | 61 (33.9%) |
|  |  | No | 119 (66.1%) |
| Use of folic acid during pregnancy | 183 | Yes | 117 (63.9%) |
|  |  | No | 66 (36.1%) |
| Caesarean section | 183 | Yes | 9 (4.9%) |
|  |  | No | 174 (95.1%) |
| Complications during pregnancy | 183 | Yes | 8 (4.4%) |
| (High blood pressure, gestational diabetes, and/or pre-eclampsia) |  | No | 175 (95.6%) |
| a) High Blood Pressure | 183 | Yes | 6 (3.3%) |
|  |  | No | 177 (96.7%) |
| b) Gestational Diabetes | 183 | Yes | 2 (1.1%) |
|  |  | No | 181 (98.9%) |
| c) Pre-eclampsia | 183 | Yes | 1 (0.6%) |
|  |  | No | 182 (99.5%) |
|  |  |  |  |
| **Newborns** | | | |
|  | **N** | **median (P25-P75)** | **min.-max.** |
| Birth weight (g) | 183 | 3520 (3200-3770) | 2175-4950 |
| Birth length (cm) | 180 | 51 (49-52) | 45-57 |
| Head circumference at birth (cm) | 178 | 35 (34-36) | 31-39 |
|  |  |  |  |
|  | **N** | **categories** | **N (%)** |
| SGA | 183 | Yes | 14 (7.7%) |
|  |  | No | 169 (92.3%) |
| Sex | 183 | Male | 93 (50.8%) |
|  |  | Female | 90 (49.2%) |
|  |  |  |  |
| **Family** | | | |
|  | **N** | **median (P25-P75)** | **min.-max.** |
| Paternal height (cm) | 176 | 181 (176-186) | 165-198 |
|  |  |  |  |
|  | **N** | **categories** | **N (%)** |
| Equivalent income | 167 | ≤1250€ | 51 (30.5%) |
|  |  | 1250-1500€ | 38 (22.8%) |
|  |  | >1500€ | 78 (46.7%) |

N = number of subjects; P25 = 25^th^ percentile; P75 = 75^th^ percentile; SGA = Small for Gestational Age
