# Supplementary material for: Expression of the sFLT1 Gene in Cord Blood Cells Is Associated to Maternal Arsenic Exposure and Decreased Birth Weight
Source: PLoS One. 2014 Mar 24;9(3):e92677. doi: 10.1371/journal.pone.0092677 (PMC3963915; doi:10.1371/journal.pone.0092677)
Supplement: Table S3 — Sequences of 5′ exonuclease PrimeTime™ assays that were used for qPCR analysis. (DOCX) [file pone.0092677.s005.docx]

**Table S3: S**equences of 5’ exonuclease PrimeTime™ assays that were used for qPCR analysis

| sFLT1: NM_001159920 |  |
| --- | --- |
| PrimeTime Probe | /56-FAM/TGCTAATCC/ZEN/TGAGAGTTGCCCTGC/3IABkFQ/ |
| PrimeTime Primer 2 (reverse) | ACCTTTCCCTTTTGAGTCCTG |
| PrimeTime Primer 1 (forward) | AACAGATAGTAACCCTGCCATG |
| sFLT1: NM_001160030 |  |
| PrimeTime Probe | /56-FAM/TGT CCC CGA /ZEN/GCC TCA GAT CAC TT/3IABkFQ/ |
| PrimeTime Primer 2 (reverse) | ACC ACT TTA GAC TGT CAT GCT |
| PrimeTime Primer 1 (forward) | GAC GAT GGT GAC GTT GAT GT |
| GNB2L1: NM_006098 (reference gene) |  |
| PrimeTime Probe | /56-FAM/AGTGGCTCT/ZEN/CATCCTGGACAGTGTAT/3IABkFQ/ |
| PrimeTime Primer 2 | CAAGCTATGGAATACCCTGGG |
| PrimeTime Primer 1 | AGAAGCGGACACAAGACAC |
| RPLP0: NM_001002 (reference gene) |  |
| PrimeTime Probe | /56-FAM/CCCTGTCTT/ZEN/CCCTGGGCATCAC/3IABkFQ/ |
| PrimeTime Primer 2 | TCGTCTTTAAACCCTGCGTG |
| PrimeTime Primer 1 | TGTCTGCTCCCACAATGAAAC |
| RPL13A: NM_012423 (reference gene) |  |
| PrimeTime Probe | /56-FAM/CTTCAGACG/ZEN/CACGACCTTGAGGG/3IABkFQ/ |
| PrimeTime Primer 2 | CTGTCACTGCCTGGTACTTC |
| PrimeTime Primer 1 | TGTTTGACGGCATCCCAC |
