# Supplementary material for: Expression of the sFLT1 Gene in Cord Blood Cells Is Associated to Maternal Arsenic Exposure and Decreased Birth Weight
Source: PLoS One. 2014 Mar 24;9(3):e92677. doi: 10.1371/journal.pone.0092677 (PMC3963915; doi:10.1371/journal.pone.0092677)
Supplement: Table S4 — Subset of genes that were associated with intrauterine growth, DNA methylation, oxidative stress, and As-related genes. Quality control: +: Quality control passed for given sequence, −: Quality control not passed for given sequence. Embryonal growth: Number of sequences Agilent array = 294 (160 unique genes); Number of sequences Agilent array after quality control filtering = 289 (158 unique genes). DNA methylation: Number of sequences Agilent array = 102 (49 unique genes); Number of sequences Agilent array after quality control filtering = 101 (48 unique genes). Oxidative stress: Number of sequences Agilent array = 275 (154 unique genes); Number of sequences Agilent array after quality control filtering = 269 (152 unique genes). (DOCX) [file pone.0092677.s006.docx]

**Table S4. S**ubset of genes that were associated with intrauterine growth, DNA methylation, oxidative stress, and As-related genes.

| **Agilent Probe ID** | **Gene Symbol** | **Gene Name** | **Sequence Agilent Array** | **Quality Control** |
| --- | --- | --- | --- | --- |
| **Process : Embryonal Growth** | | | | |
| A_23_P100704 | MAPK7 | mitogen-activated protein kinase 7 | GTGAGGCTCGGCTTGGATTATTCTGCAGGTTCATCTCAGACCCACCTTTCAGCCTTAAGC | + |
| A_23_P102183 | PNO1 | partner of NOB1 homolog (S. cerevisiae) | TCTTGCCTTTGGACTCTGGTGAAAAATACTTTACAGTGGTCGGTCACAAGAAACCATCTG | + |
| A_23_P104607 | PSMC3 | proteasome (prosome, macropain) 26S subunit, ATPase, 3 | ACAAGGAGAAGTTTGAGAACTTGGGGATCCAACCTCCAAAAGGGGTGCTGATGTATGGGC | + |
| A_23_P106127 | KIAA0586 | KIAA0586 | CATTTTACCTTGGCTTAAAACCCTCTCTCAGACTGTTTGGTTTTTGAGCATATTCTGAAA | + |
| A_23_P109950 | ACVR2B | activin A receptor, type IIB | TCATGTAGCAGAGACGATGTCACGAGGCCTCTCATACCTGCATGAGGATGTGCCCTGGTG | + |
| A_23_P111402 | RSPO3 | R-spondin 3 | GTCATGTGTTTTCTTTCTCAATGAGGGAAAAACAATTTTATTACCTGCTTAATGGTCCAC | - |
| A_23_P111481 | SRRT | serrate RNA effector molecule homolog (Arabidopsis) | GTCCTATGAAGCTCTGAGAATTTTTTGTACGATCAGCCTTACTGCTAATAAAAGCACTTC | + |
| A_23_P111487 | SRRT | serrate RNA effector molecule homolog (Arabidopsis) | GAGGAAGTGAAAAAGGAAGTCGCGTTTTTTAACAACTTCCTCACTGATGCTAAGCGCCCA | + |
| A_23_P111657 | SHH | sonic hedgehog | CGCGGGCGCCTACGCGCCGCTCACGGCCCAGGGCACCATTCTCATCAACCGGGTGCTGGC | + |
| A_23_P111835 | DLD | dihydrolipoamide dehydrogenase | AATTTTACATGGCTGGAGCTAGAATTTGATATGTGAACAGTTGTGTTTGAAGCACAGTGA | + |
| A_23_P114783 | PARP1 | poly (ADP-ribose) polymerase 1 | GACTAGTCCTATGGAAAAAACCAAAGCTTCGTTAGAATGTCTGCCTTACTGGTTTCCCCA | + |
| A_23_P121646 | SLC34A2 | solute carrier family 34 (sodium phosphate), member 2 | CCTTGACCCCCCTGATTGGAATCGGCGTGATAACCATTGAGAGGGCTTATCCACTCACGC | + |
| A_23_P12329 | APH1A | anterior pharynx defective 1 homolog A (C. elegans) | TTTTCTCAGGCCTGAGGGGGAACCATTTTTGGTGTGATAAATACCCTAAACTGCCTTTTT | + |
| A_23_P127322 | NODAL | nodal homolog (mouse) | ACTGAGGGCTCACTTGCCATTGAGATTTTCCACCAGCCAAAGCCCGACACAGAGCAGGCT | + |
| A_23_P12920 | RAD9A | RAD9 homolog A (S. pombe) | TCCTGTCTGTCTTCCGCTCACTGGCGATGCTGGAGAAGACGGTGGAAAAATGCTGCATCT | + |
| A_23_P131020 | GATAD2A | GATA zinc finger domain containing 2A | AAAGGATCAGGTCTGCTTTTAGTTTCATTTTTGTTTCTTTCCCGTCCCACTCTTTAAAAA | + |
| A_23_P131846 | SNAI1 | snail homolog 1 (Drosophila) | AACAATGTCTGAAAAGGGACTGTGAGTAATGGCTGTCACTTGTCGGGGGCCCAAGTGGGG | + |
| A_23_P132260 | PES1 | pescadillo homolog 1, containing BRCT domain (zebrafish) | GTGCAGCTGCCCCCACACCTTTCACCCTTTGTGACCGAGAAGGAAGGAGATTACGTTCCA | + |
| A_23_P133036 | SLC34A2 | solute carrier family 34 (sodium phosphate), member 2 | ATAGTAAATGGTCTGCATGATTTGTGCTTCTAGTGCTCTCATTTGGAAATGAGGCAGGCT | + |
| A_23_P133095 | RAPGEF2 | Rap guanine nucleotide exchange factor (GEF) 2 | CTGCTAATCTCAGTTCGCTCTGTGATGTCAAGTGCAGAATGTACAATTAACTGGTGATTT | + |
| A_23_P138967 | SDHD | succinate dehydrogenase complex, subunit D, integral membrane protein | CATACTTTGAAGAATTGATGTATGCCTCTTTGCCTCTGCTTTGTCATGCCATTAAGCTCA | + |
| A_23_P140241 | CHD8 | chromodomain helicase DNA binding protein 8 | GTACAGACAGCTAACTCTAGAAATGGGAAAAAGGGTCATCACACTGAAACGGTGTTCAAC | + |
| A_23_P141329 | RPS6KB1 | ribosomal protein S6 kinase, 70kDa, polypeptide 1 | AGGATTTTCATGTTGATGACTCGAAACTGACAGTATTAAGGGTAGGATGTTGCTTCTGAA | + |
| A_23_P141434 | ACACA | acetyl-CoA carboxylase alpha | GTAATAGAGGAAAACATCAAATGCATCAGCAGAGACTACGTCCTCAAGCAAATCCGCAGC | + |
| A_23_P141738 | SS18 | synovial sarcoma translocation, chromosome 18 | CTAGCCCAAAGGTAAGTTGCTATTTTCATCACAGTTGCCTATGCCCAGGGAATAAGATGT | + |
| A_23_P142013 | XAB2 | XPA binding protein 2 | GCATCCTGGACCTGCGTATCGCAACACCCCAGATCGTCATCAACTATGCCATGTTCCTGG | + |
| A_23_P142560 | ZEB2 | zinc finger E-box binding homeobox 2 | CTTTTAATCTGTGTTTCTGCAAGTGCCATCCTTGTACAGTGTTAAGAGGGTAACATGGGT | + |
| A_23_P142835 | DCTN1 | dynactin 1 | CCTCCTCCTTCAGGCATTGCTACTCTGGTCTCTGGCATTGCTGGTGAAGAACAGCAGCGA | + |
| A_23_P145114 | GCLC | glutamate-cysteine ligase, catalytic subunit | AGAATGCCTGGTTTTCGTTTGCAATTTGCTTGTGTAAATCAGGTTGTAAAAAGGCAGATA | + |
| A_23_P145146 | PDCD2 | programmed cell death 2 | ACAGAAGAATTTGTGTGGAAGCAGGATGTAACAGATACACCGTAAAGGCATCTTAAAGCC | + |
| A_23_P145153 | PDCD2 | programmed cell death 2 | TTGTAATAGAAACAGAAGATGAGATTATGCCTGAGGTTGTGGAAAAGGAAGATTACTCAG | + |
| A_23_P146354 | POMT1 | protein-O-mannosyltransferase 1 | CATCCATACAGCTCCATGCCTTTGTCTTTTTTAAATGTAATTAAAAAAGGAACCAACTGG | + |
| A_23_P146361 | POMT1 | protein-O-mannosyltransferase 1 | CCTCCTTTTGTGCAAAGTTAATTTTTTCTCGACAATAAAGATATTCCGTGTCTTCACCCC | + |
| A_23_P151662 | MAX | MYC associated factor X | CATCATAATGCACTGGAACGAAAACGTAGGGACCACATCAAAGACAGCTTTCACAGTTTG | + |
| A_23_P152768 | TUBG1 | tubulin, gamma 1 | CGATGTGGTGGTCCAGCCTTACAATTCACTCCTCACACTCAAGAGGCTGACGCAGAATGC | + |
| A_23_P152818 | KAT7 | K(lysine) acetyltransferase 7 | CAAAGTAATGTCCACTTTTCCCTTTCATGCTGCATATTAACTGGTTAATTATACTGCAGA | + |
| A_23_P153930 | ACVR2A | activin A receptor, type IIA | TTTCTGTGTAAAATGAGTAGGATGTCTCTTGGAAATGTTAAGAAAGAAGACCCTTTGTTG | + |
| A_23_P154938 | HIRA | HIR histone cell cycle regulation defective homolog A (S. cerevisiae) | ATTTCCTTTGGCCGATAATCAGGATTTCCCTATAAGTCACTTGGACATTGGTCACTTGTA | + |
| A_23_P155969 | PLK4 | polo-like kinase 4 | TTGTCTTCCTAAATCAGCACAACTTTTGAAATCTGTTTTTGTGAAAAATGTTGGTTGGGC | + |
| A_23_P156970 | MEST | mesoderm specific transcript homolog (mouse) | AAGGACCAATAGCATCTGTGCCAGAGATGTACTGTTATTAGCTGGGAAGACCAATTCTAA | + |
| A_23_P156977 | HUS1 | HUS1 checkpoint homolog (S. pombe) | CCACAAAGGCCTTATGCAATATTGTGAATAACAAGATGGTGCATTTTGATCTGCTTCATG | + |
| A_23_P157695 | RDH10 | retinol dehydrogenase 10 (all-trans) | TCCTAGGAACCGATACATGCACACAACTGCTTCAAATGGTAGGATGCTCTTCATGAAGGT | + |
| A_23_P15937 | SMAD2 | SMAD family member 2 | AAACAGCACTTGAGGTCTCATCAATTAAAGCACCTTGTGGAATCTGTTTCCTATATTTGA | + |
| A_23_P159390 | TOPBP1 | topoisomerase (DNA) II binding protein 1 | GAAGTAGTTCCATACTTGATGTTCTGACTGGAATCAATGTACAGCAACGAAGACTAGCAC | + |
| A_23_P161624 | FOSL1 | FOS-like antigen 1 | CTGGCCTCTCTAGCACAATTTGCACTAAATCAGAGACAAAATATTTCCCATTTGTGCCAG | + |
| A_23_P163027 | PARP2 | poly (ADP-ribose) polymerase 2 | GGTCCGTATGCGGTACCTTTTAAAGGTTCAGTTTAATTTCCTTCAGCTGTGGTGAATGTT | + |
| A_23_P164536 | PIK3C3 | phosphoinositide-3-kinase, class 3 | CCTGAGTTCTGCTTCCTTGGATGTCATTGCTTAAATATAGTCTTGAAGGGCTTGTTTTGA | + |
| A_23_P16648 | PCSK4 | proprotein convertase subtilisin/kexin type 4 | GAGAGGCTGGAGCACCCCAAAAGCCAGGGGAAAGTGGAGGGAGAGAAACGTGACACTGTC | + |
| A_23_P167389 | ARAP3 | ArfGAP with RhoGAP domain, ankyrin repeat and PH domain 3 | ATATTTGATACGTAGGGGTTCCATGAGAGATTTTGGGTTTTAAAGGAATGGTTTTACTGC | + |
| A_23_P18939 | RASA1 | RAS p21 protein activator (GTPase activating protein) 1 | ATGTCTCCTTTGCTCTTGCCAAAAAATAGCACACTTTTCCACATTCCAGTGATGTGTGAG | + |
| A_23_P19313 | TBP | TATA box binding protein | GACAAGTTGGTTTGAGGGAGAAAACTTTAAGTGTTAAAGCCACCTCTATAATTGATTGGA | + |
| A_23_P202280 | VCL | vinculin | GGGGCCTCTTCAAATTAGAAGACATTTATACTCTTTTTTCATGGACACTTTGAAATGTGT | + |
| A_23_P204579 | TDG | thymine-DNA glycosylase | ATGACGGACATCCACTAGAGATGGGTTTGAGGATTTTCCAAGCGTGTAATAATGATGTTT | + |
| A_23_P204581 | TXNRD1 | thioredoxin reductase 1 | CAAAAGCAAGTCATGGCTAGAGTATCCATGCAAGGTGTCTTGTTGCATGGAAGGGATAGT | + |
| A_23_P205549 | MAX | MYC associated factor X | TGAAGGGTTTTGTGTGTTTAGGCCTCATTTCTTTGTCTTTTTCCTACTCCGTTCCTGGCA | + |
| A_23_P207557 | HNF1B | HNF1 homeobox B | GGGTGCATGTGAGGATGAAAGGAGTGAATGTATAAAGACACCTTTCCCGATAACCCATAC | + |
| A_23_P208880 | UHRF1 | ubiquitin-like with PHD and ring finger domains 1 | TGTATTAGGGAAGAATGAGACAATTTTGTGTAGGCTTTTTCTAAAGTCCAGTACTTTGTC | + |
| A_23_P209879 | ATF2 | activating transcription factor 2 | TCATGTAAACGGTTAACAAGCTTACCAAGGTTAGCAAAACTTTCATTGTAAATCAGTCTG | + |
| A_23_P210358 | LIMS1 | LIM and senescent cell antigen-like domains 1 | TCTACCCATATTTAAAGCTATATCTCAAAGCAGTTGAGAGAAGAGGACCTATATGAATGG | + |
| A_23_P210853 | GINS1 | GINS complex subunit 1 (Psf1 homolog) | TCTTGCCAAATGCATTACGATTTCACATGGCTGCTGAAGAAATGGAGTGGTTTAATAATT | + |
| A_23_P2114 | LAMTOR1 | late endosomal/lysosomal adaptor, MAPK and MTOR activator 1 | CAGCCTCACTGCGGCTTATACAGTACCCTAACCTGCTACTAATCACAGAGAAAAATGTGA | + |
| A_23_P212579 | DAG1 | dystroglycan 1 (dystrophin-associated glycoprotein 1) | GAAGATTGCCTTGGTAAAGAAACTGGCCTTCGCCTTTGGAGACCGAAACTGTAGCACCAT | + |
| A_23_P212749 | HTT | huntingtin | CTTCCCCTCAGTTGTTTCTAAGAGCAGAGTCTCCCGCTGCAATCTGGGTGGTAACTGCCA | + |
| A_23_P213944 | HBEGF | heparin-binding EGF-like growth factor | CCCAGTGGAAAATCGCTTATATACCTATGACCACACAACCATCCTGGCCGTGGTGGCTGT | + |
| A_23_P214121 | AMD1 | adenosylmethionine decarboxylase 1 | TTGCTTCGCCCCAGAAGATTGAAGGTTTTAAGCGTCTTGATTGCCAGAGTGCTATGTTCA | + |
| A_23_P215406 | RAC1 | ras-related C3 botulinum toxin substrate 1 (rho family, small GTP binding protein Rac1) | TGTTAGTCGCTAACTTAGTAAGTGCTTTTCTTATAGAACCCCTTCTGACTGAGCAATATG | + |
| A_23_P216622 | FKTN | fukutin | TTACAGTTCATAAGTGAAACAGACTAATTCAATGGCAATACCTTTTGTATAGGTCCTGTG | + |
| A_23_P216649 | ABCA1 | ATP-binding cassette, sub-family A (ABC1), member 1 | CAAAATTCCATTACAGGGGCAGTGCCTTTGTAGCCTATGTCTTGTATGGCTCTCAAGTGA | + |
| A_23_P217804 | CDK11A/CDK11B | cyclin-dependent kinase 11B | AGAGGAGAAGAAGATGATTCTTTGGCCATCAAACCACCCCAGCAAATGTCTCGGAAAGAA | + |
| A_23_P217872 | ARID1A | AT rich interactive domain 1A (SWI-like) | AGGCCTGTGCAGTAGAGTGTAGACCCTTTCATGTACTGTACTGTACACCTGATACTGTAA | + |
| A_23_P22378 | SOX11 | SRY (sex determining region Y)-box 11 | GTGGAGGGGAGAGAAGAAGATGCTGATGATATTGATAAGATGTCGTGACGCAAAGAAATT | + |
| A_23_P250404 | RAD50 | RAD50 homolog (S. cerevisiae) | TTGCACATGCTCTGGTTGAGATAATAAAAAGTCGCTCACAGCAGCGTAACTTCCAGCTTC | + |
| A_23_P251480 | NBN | nibrin | TTGGGAAGAAACGTGAACTCAAGGAAGACTCACTATGGTCAGCTAAAGAAATATCTAACA | + |
| A_23_P25176 | TBX5 | T-box 5 | GGCATGGAGGGAATCAAAGTGTTTCTCCATGAAAGAGAACTGTGGCTAAAATTCCACGAA | + |
| A_23_P252371 | RBBP8 | retinoblastoma binding protein 8 | GGCAAGGAGCAGAAGACATAGACGTTGAAACAGAAACAGAAGGATGAAGGACAGTTTTTT | + |
| A_23_P253158 | EP400 | E1A binding protein p400 | TAGATGCTACCGTTGACCTGAGTTAAATTCATTTAGTCGTGTACGTAAAAACTCTCCTTT | + |
| A_23_P254081 | LIAS | lipoic acid synthetase | TTTTTAAAATTTGATTCCAGTTAATAACAGAGGTGGTGCCAGAATGCCTGGACTGCAGTG | + |
| A_23_P254801 | PLCG1 | phospholipase C, gamma 1 | CTGGGCCAGGGAAACAAAGTTTACATTGTCCTGTAGCTTTAAAACCACAGCGGGCAGGGT | + |
| A_23_P256603 | MLLT4 | myeloid/lymphoid or mixed-lineage leukemia (trithorax homolog, Drosophila); translocated to, 4 | CAAGCTCATGCTGGAGTGGCAGTTCCAGAAGAGACTCCAGGAGTCGAAGCAGAAGGACGA | + |
| A_23_P256716 | SMARCA5 | SWI/SNF related, matrix associated, actin dependent regulator of chromatin, subfamily a, member 5 | GCTTCACAAACTTGGATTTGACAAAGAAAATGTTTATGATGAATTGCGACAGTGTATTCG | + |
| A_23_P259641 | EZH2 | enhancer of zeste homolog 2 (Drosophila) | TTTATTGCCTTCTCACCAGCTGCAAAGTGTTTTGTACCAGTGAATTTTTGCAATAATGCA | + |
| A_23_P2601 | HSP90B1 | heat shock protein 90kDa beta (Grp94), member 1 | CCCTTCTCCCCTGCACTGTAAAATGTGGGATTATGGGTCACAGGAAAAAGTGGGTTTTTT | + |
| A_23_P26021 | COPS2 | COP9 constitutive photomorphogenic homolog subunit 2 (Arabidopsis) | TGCTTTTTTGATCAACTGGTTTGTGTTTTGCTGCTGCATTTATCCCAAGAAAAACAGCTT | + |
| A_23_P27167 | RNASEH1 | ribonuclease H1 | CCATTGAACAAGCAAAGACTCAAAACATCAATAAACTGGTTCTGTATACAGACAGTATGT | + |
| A_23_P27346 | SMAD4 | SMAD family member 4 | AGGAGAAACACATTCTTATTAATTCTTCACCTGTTATGTATGAAGGAATCATTCCAGTGC | + |
| A_23_P303390 | MYCN | v-myc myelocytomatosis viral related oncogene, neuroblastoma derived (avian) | GGCCCACCCGGAGACACCCGCGCAGAATCGCCTCCGGATCCCCTGCAGTCGGCGGGAGTG | + |
| A_23_P306500 | KRAS | v-Ki-ras2 Kirsten rat sarcoma viral oncogene homolog | CATCATTTGGTTGCGCTGACCTAGGAATGTTGGTCATATCAAACATTAAAAATGACCACT | + |
| A_23_P306507 | KRAS | v-Ki-ras2 Kirsten rat sarcoma viral oncogene homolog | CTGAGTCACACTGCATAGGAATTTAGAACCTAACTTTTATAGGTTATCAAAACTGTTGTC | + |
| A_23_P308552 | DLX3 | distal-less homeobox 3 | AGACGGTGAACCCCTACACCTACCACCACCAATTCAATCTCAATGGGCTTGCAGGCACGG | + |
| A_23_P318860 | APLNR | apelin receptor | CCAACATGGGCAAGGGTGGAGAACAGATGCACGAGAAATCCATCCCCTACAGCCAGGAGA | + |
| A_23_P322519 | FOSL1 | FOS-like antigen 1 | GACTGACAAACTGGAAGATGAGAAATCTGGGCTGCAGCGAGAGATTGAGGAGCTGCAGAA | + |
| A_23_P323272 | OSR1 | odd-skipped related 1 (Drosophila) | ACGTTTTCTTTTTTATTTTTGTCTTTGACCATATAAGCTTGTAACTCTGACTGCGGAGAG | - |
| A_23_P333650 | RAD9B | RAD9 homolog B (S. pombe) | CCATTATGCTACTTGTGAGGCAGAAGAGTTTTCTGTGAAGGAAAAAAGCCCATTAGAGTT | + |
| A_23_P334555 | PES1 | pescadillo homolog 1, containing BRCT domain (zebrafish) | ACTGTAGAGCGTTTAAAGGACAATAAGCCCAACTACAAACTCGACCACATCATCAAGGAA | + |
| A_23_P34093 | G6PD | glucose-6-phosphate dehydrogenase | CCACGGAGGCAGACGAGCTGATGAAGAGAGTGGGTTTCCAGTATGAGGGCACCTACAAGT | + |
| A_23_P344694 | MLLT4 | myeloid/lymphoid or mixed-lineage leukemia (trithorax homolog, Drosophila); translocated to, 4 | GCACATGTGTAGGTGTAATGGACATTGTAACATACTTCTCACTTAAAATGAAATTCTGTC | + |
| A_23_P34606 | MTOR | mechanistic target of rapamycin (serine/threonine kinase) | TTTCTGGTAACTGGAGGCCCAGATGTGCCCATCACGTTTTTTCTGAGGCTTTTGTACTTT | + |
| A_23_P34800 | NASP | nuclear autoantigenic sperm protein (histone-binding) | GCACAGTTCAGCAAATCTATTGAAGTCATTGAGAACAGAATGGCTGTACTAAACGAGCAG | + |
| A_23_P348323 | PCGF2 | polycomb group ring finger 2 | AACTGGCTGCATATAGGAGTCAGTTAGAATTGTTTCTTTCTCTCCCCGTTTCTCTCCCCA | + |
| A_23_P352879 | GCLC | glutamate-cysteine ligase, catalytic subunit | CTCTCAGCCCGTGTGTATAATATGAAGACCAAATGATAGAACTGTACTGTTTTCTGGGCC | + |
| A_23_P361495 | ATF7 | activating transcription factor 7 | AGCTTGTTCCATCAAGTTAGGGATTAATCGATTCCTTTATGTTTTATAAGAGTTTACCGG | + |
| A_23_P365738 | ARC | activity-regulated cytoskeleton-associated protein | CCCACAGATTTTATTTTTGCACATAAGCCATAACCAATCCTCAAGGCTGGCACAGGCTTT | + |
| A_23_P366376 | TDGF1 | teratocarcinoma-derived growth factor 1 | TAGGTGGTGGTAGAGAAGCAAGTAAAAAGGCTAAATGGAAGGGCAAGTTTCCATCATCTA | + |
| A_23_P37111 | DICER1 | dicer 1, ribonuclease type III | CGGTCGCTGTTGCACCTGATAGTAAGGGACAGATTTTTAATGAACATTGGCTGGCATGTT | + |
| A_23_P384072 | EPN1 | epsin 1 | GCAGGAGAGCTGGAGCTGCTGGCAGGAGAGGTGCCGGCCCGAAGCCCTGGGGCGTTTGAC | + |
| A_23_P384761 | GATA4 | GATA binding protein 4 | TTCGAAGCAAACAAACACAACACAACAGAATTCCTGGAAAGAAGACGACTGCTAAGACAC | + |
| A_23_P387071 | ZNF830 | zinc finger protein 830 | CTGAAGTGTTATATACCAAAATGCCAACTTTTCCACGACAGACTTAACTGTATAATTTCG | + |
| A_23_P39034 | SMARCA4 | SWI/SNF related, matrix associated, actin dependent regulator of chromatin, subfamily a, member 4 | CTGGCATCAGTAGCATCTGTAACAGCATTAACTGTCTTAAAGAGAGAGAGAGAGAATTCC | + |
| A_23_P39040 | SMARCA4 | SWI/SNF related, matrix associated, actin dependent regulator of chromatin, subfamily a, member 4 | AAGGAGCGCATTCGCAACCACAAGTACCGCAGCCTCAACGACCTAGAGAAGGACGTCATG | + |
| A_23_P40693 | EP300 | E1A binding protein p300 | TTTTTGAATCTTTCGTAGCCTAAAAGACAATTTTCCTTGGAACACATAAGAACTGTGCAG | + |
| A_23_P407203 | ZEB2 | zinc finger E-box binding homeobox 2 | CAGAATTAAATGTCACCACAAAAGCAGATGTTAACATAAGCCCAAATATGCTTTTTAGCC | + |
| A_23_P409287 | HNF1B | HNF1 homeobox B | AGTGTCTATTAGTTTTCTTTGTAAAGGTCAGAGTCAAAATTTCAAAAGTGATCTGTCCCC | + |
| A_23_P40952 | RAF1 | v-raf-1 murine leukemia viral oncogene homolog 1 | TAAGGTAGCAGGCAGTCCAGCCCTGATGTGGAGACACATGGGATTTTGGAAATCAGCTTC | + |
| A_23_P421465 | EP400 | E1A binding protein p400 | TCACAGAAAACGCTTTATTAGTGAACCTTGGGACCATGTCACGCAAGAGATTCAGCACTG | + |
| A_23_P424002 | POU2F1 | POU class 2 homeobox 1 | GGAACATTTGCCTAATTTGTAATAAAACACTGTCTTTTCAGGGTTGCTTCATGGGTTGGA | + |
| A_23_P433071 | BECN1 | beclin 1, autophagy related | TAGAAACTTTTGTCTTTACTGAGATGAGGATATGTTTGAGATGCACAGTTGGATAATGTG | + |
| A_23_P43484 | CDKN2A | cyclin-dependent kinase inhibitor 2A (melanoma, p16, inhibits CDK4) | GGGTTACTGGCTTCTCTTGAGTCACACTGCTAGCAAATGGCAGAACCAAAGCTCAAATAA | + |
| A_23_P43490 | CDKN2A | cyclin-dependent kinase inhibitor 2A (melanoma, p16, inhibits CDK4) | TTAAAAATGTCCTGCCTTTTAACGTAGATATATGCCTTCCCCCACTACCGTAAATGTCCA | + |
| A_23_P436138 | MAX | MYC associated factor X | TGATTGTTTTGACCCCTTACACAAATGTCTTACTCCTGGCTTTAATTAAGCTGCTTGAGG | + |
| A_23_P436353 | MLLT4 | myeloid/lymphoid or mixed-lineage leukemia (trithorax homolog, Drosophila); translocated to, 4 | CTATGAGAAAACATCAAAACTGCTGTAGTTTTCCATTTCTACTGTATTTCAGTTGCAACC | + |
| A_23_P4425 | FLII | flightless I homolog (Drosophila) | GACATCATGTTGCTAGACAATGGCCAAGAGGTCTACATGTGGGTGGGGACCCAGACTAGC | + |
| A_23_P45140 | KRAS | v-Ki-ras2 Kirsten rat sarcoma viral oncogene homolog | TTCAGTTGAGACCTTCTAATTGGTTTTTACTGAAACATTGAGGGAACACAAATTTATGGG | + |
| A_23_P48637 | HIF1A | hypoxia inducible factor 1, alpha subunit (basic helix-loop-helix transcription factor) | TTGTAAGAAGGCAGTAACCTTTCATCATGATCATAGGCAGTTGAAAAATTGTTACCACCG | + |
| A_23_P48663 | OTX2 | orthodenticle homeobox 2 | TGCATGCAGAGGTCCTATCCCATGACCTATACTCAGGCTTCAGGTTATAGTCAAGGATAT | + |
| A_23_P49033 | SNX1 | sorting nexin 1 | TCTCGGGTGACTCAATATGAAAGGGACTTCGAGAGGATTTCAACAGTGGTCCGAAAAGAA | + |
| A_23_P49060 | SPINT1 | serine peptidase inhibitor, Kunitz type 1 | GAGAAGTCTCAGCTAAGCTCACGTCCTGAGAAAGCTCAAAGGTTTGGAAGGAGCAGAAAA | + |
| A_23_P49481 | USP7 | ubiquitin specific peptidase 7 (herpes virus-associated) | GACCACTTCAACAAAGCCCCAAAGAGGAGTCGCTACACTTACCTTGAAAAGGCCATTAAA | + |
| A_23_P500936 | FOXA2 | forkhead box A2 | CAGGTCTCGGGTCCGATTAATTTATGGTTTCTGCGTGCTTTATTTATGGCTTATAAATGT | + |
| A_23_P501460 | CDK11A/CDK11B | cyclin-dependent kinase 11B | CTACAGCCAGCTGGGTGACGACGACCTGAAGGAGACGGGCTTCCACCTTACCACCACGAA | + |
| A_23_P50815 | TTYH1 | tweety homolog 1 (Drosophila) | GGCTCTGACCCCCTGATCTCAACTCGTGGCACTAACTTGGAAAAGGGTTGATTTAAAATA | + |
| A_23_P50817 | TTYH1 | tweety homolog 1 (Drosophila) | CGATGACACAGACGATGACGACCCTTTCAACCCTCAGGAATCCAAGCGCTTTGTGCAGTG | + |
| A_23_P55045 | SLC25A19 | solute carrier family 25 (mitochondrial thiamine pyrophosphate carrier), member 19 | GCAGTTCTCTTGCTACAGCTCCTTGAAGCACCTGTACAAGTGGGCCATACCAGCCGAAGG | + |
| A_23_P58482 | MGAT1 | mannosyl (alpha-1,3-)-glycoprotein beta-1,2-N-acetylglucosaminyltransferase | CCACTTAGCCCTTCTCTCCTTGCAGCCTAGCAGTTTATAGTTCTGAGATGGAAAGTTGAA | + |
| A_23_P58763 | PELO | pelota homolog (Drosophila) | TGGTGGACAGTGTGAAAGAGAATGCAGGCACCGTTAGGATATTCTCTAGTCTTCACGTTT | + |
| A_23_P58770 | HAND1 | heart and neural crest derivatives expressed 1 | TCGTGGTGGTTTTATTTTATTTTTCTTTTTGTCGCTGCACTTCCTGTTTAGTTCCAAGGG | + |
| A_23_P59418 | NRF1 | nuclear respiratory factor 1 | GGAATTGCATTTTTTAAAGCACCACTCTTGATTTTCTGGGATTGGTGAAGAAACTGCATT | + |
| A_23_P59426 | PAXIP1 | PAX interacting (with transcription-activation domain) protein 1 | TCTTGTTTTCCAGCTGCTTTCCTGGGGGATCAGACTGTGAAGCAGGAAGACAGATATAAT | + |
| A_23_P59657 | CUL1 | cullin 1 | CTTCTGGAAGGGTCTGACTGTGTGACCCGCAGCAAATAGTTCATGTTGGAAAGAATGAAA | + |
| A_23_P59677 | RINT1 | RAD50 interactor 1 | GCTGAGTATGTCTGTTGAAGACGAGCAGAGATATTAAATTATAACCAACTTTCAATTTCC | + |
| A_23_P60248 | TXN | thioredoxin | GGACAAAAGGTGGGTGAATTTTCTGGAGCCAATAAGGAAAAGCTTGAAGCCACCATTAAT | + |
| A_23_P60387 | NOTCH1 | notch 1 | ACCCATGGTACCAATCATGAATCTTTGTTTCAGGTTCAGTATTATGTAGTTGTTCGTTGG | + |
| A_23_P60517 | FXN | frataxin | GCCCCCAAGTTCTGATTTTTAATTTCTATGGAAGATTTTTTGGATTGTCGGATTTCCTCC | + |
| A_23_P60899 | TGS1 | trimethylguanosine synthase 1 | AAGATTGCTGAACACTTTGCTGGCCGTGTTAGTCAGTCCTTCAAGTGTGACGTTGTAGTA | + |
| A_23_P63371 | TAL1 | T-cell acute lymphocytic leukemia 1 | ACAAAACAAAACAACATTTTGAGAACAAAGATGACCATAACCACTGAAGGGAATCACATC | + |
| A_23_P66608 | KAT2A | K(lysine) acetyltransferase 2A | CTTGAGTGATGGCTTCAGGGGTTGGAAGTTCAGCCCAAACTGAAGGGGGCCATGCCTTGT | + |
| A_23_P71419 | COPS5 | COP9 constitutive photomorphogenic homolog subunit 5 (Arabidopsis) | GAATACGTTGAGTTCTTCTAGCTTGCTTACTAATGCAGACTATACCACTGGTCAGGTCTT | + |
| A_23_P75839 | TSG101 | tumor susceptibility gene 101 | GTTGAGCTCTTCTTAAAGTATTCTTCTCTTCCTTTTATCAGTAGGTGCCCAGAATAAGTT | + |
| A_23_P76654 | CDX2 | caudal type homeobox 2 | AGCTGGAGAAGGAGTTTCACTACAGTCGCTACATCACCATCCGGAGGAAAGCCGAGCTAG | - |
| A_23_P76774 | GSC | goosecoid homeobox | TACCTAACTCGAAGGACTTGCACAGACAGACGATGCTACTTTCTTGCACACGCGCTGCCT | + |
| A_23_P7697 | SNX2 | sorting nexin 2 | GCTGATAAAATACTGGGAAGCATTCCTACCTGAAGCCAAAGCCATTGCCTAGCAATAAGA | + |
| A_23_P77223 | MESP1 | mesoderm posterior 1 homolog (mouse) | ACCTTCGAAGTGGTTCCTTGGCAGACTGCCTTTCCTGGAAGAGGGCACGGGCGATCCCGA | + |
| A_23_P78281 | GNA13 | guanine nucleotide binding protein (G protein), alpha 13 | CTGAATTCTCCTGCAGTGCCAAGTATCAAAGGTGTCCTTAAATACTTGTAGGGATGAGGT | + |
| A_23_P78372 | THOC1 | THO complex 1 | CTGATGGATTTACAACTGAGTGACAGTAACTTTCGTCGACACATCCTGTTGCAGTATCTC | + |
| A_23_P79221 | ACVR1 | activin A receptor, type I | AAATGTAATGTCAGACTTTGCTGCATTTTACACATGTGCTGATGTTTACAATGATGCCGA | + |
| A_23_P81529 | ISL1 | ISL LIM homeobox 1 | GGTAGCAACACTGTGAAGACAATCATGGGATTTTACTAGAATTAAACAACAAACAAAACG | + |
| A_23_P82169 | SOX4 | SRY (sex determining region Y)-box 4 | GCGGAGGAGGACACGAACTGGAAGGGGGTTCACGGTCAAACTGAAATGGATTTGCACGTT | + |
| A_23_P85543 | RNF2 | ring finger protein 2 | CCTTGATACAGCCAGTGAGAAGCAGTATACCATTTATATAGCAACAGCCAGTGGCCAGTT | + |
| A_23_P88731 | RAD51 | RAD51 homolog (S. cerevisiae) | TTCTTTGGTTTTGGAGGAGGGGTATGAAGTATCTTTGACATGGTGCCTTAGGAATGACTT | + |
| A_23_P89310 | EPN2 | epsin 2 | TGGAGGAAGTCACCCAGCTGTTTCTCAGTCCCAGAGGCCGGTGGCTGGTTTTGAACTTGT | + |
| A_23_P89410 | BECN1 | beclin 1, autophagy related | CAGTTTAACTCTGAGGAGCAGTGGACAAAAGCTCTCAAGTTCATGCTGACGAATCTTAAG | + |
| A_23_P91390 | THBD | thrombomodulin | ACAGGGTCTGCAAGGTCTTTGGTTCAGCTAAGCTAGGAATGAAATCCTGCTTCAGTGTAT | + |
| A_23_P9166 | TLN1 | talin 1 | CTTTGAGGAGCAGATACTAGAAGCTGCCAAGTCCATTGCAGCAGCCACCAGTGCACTGGT | + |
| A_23_P930 | APH1A | anterior pharynx defective 1 homolog A (C. elegans) | AGATGGCCTATGTTTCTGGTCTCTCCTTCGGTATCATCAGTGGTGTCTTCTCTGTTATCA | + |
| A_23_P94879 | F2 | coagulation factor II (thrombin) | CCTGAAGAAGTGGATACAGAAGGTCATTGATCAGTTTGGAGAGTAGGGGCCACTCATATT | + |
| A_23_P96144 | BMPR2 | bone morphogenetic protein receptor, type II (serine/threonine kinase) | CCATGGTGTCTAAAGATATAGGAATGAACTGTCTGTGAAATGTTTTCAAGCCTATGGAGT | + |
| A_23_P99036 | ACVR1B | activin A receptor, type IB | CTGCTGACAATAAAGATAATGGCACCTGGACACAGCTGTGGCTTGTTTCTGACTATCATG | + |
| A_23_P99260 | METAP2 | methionyl aminopeptidase 2 | ATCCAGTTCTGCACCTATACCCTCTGGTGTTGCTTTTTAACCTTCCTGGAATCCATTTTC | + |
| A_23_P99710 | KIAA0586 | KIAA0586 | GCAAGTTGAACACAAACCATCACAAAGTTACCTACGTGTTAGAAATAAATCTGATATTGC | + |
| A_24_P102283 | ZEB2 | zinc finger E-box binding homeobox 2 | CCTGTTTATTGATTTTTATGAGTTTGTTAGAACATGCGCTTAATGCTTTTATACTCCGGG | + |
| A_24_P106542 | RSPO3 | R-spondin 3 | TGTCCCCCAACAAATGAGACAAGAAAGTGTACAGTGCAAAGGAAGAAGTGTCAGAAGGGA | + |
| A_24_P128145 | ATF2 | activating transcription factor 2 | CAATGTGCTGCTTACAAGTTCTGACTCAAGTGTAATTATTCAGCAGGCAGTACCTTCACC | + |
| A_24_P136866 | SLC8A1 | solute carrier family 8 (sodium/calcium exchanger), member 1 | CCACCTTTTCAATGGGATTTCATCTGTTAGTTACTGTGAGTCTCTTATTTTCCCATGTGG | + |
| A_24_P140608 | HBEGF | heparin-binding EGF-like growth factor | CCCATCTGTAGTAATTTATTGTCTGTCTACATTTCTGCAGATCTTCCGTGGTCAGAGTGC | + |
| A_24_P141736 | METAP2 | methionyl aminopeptidase 2 | AACAATCTAGGTTTTAGCTGTATGAGCTATGTTTATTATGGTGCTAATGTTCAGTAGCCA | + |
| A_24_P150361 | HSP90B1 | heat shock protein 90kDa beta (Grp94), member 1 | AAGAGATTTTCCTGAGAGAACTGATTTCAAATGCTTCTGATGCTTTAGATAAGATAAGGC | + |
| A_24_P150874 | GNA13 | guanine nucleotide binding protein (G protein), alpha 13 | GTATATTTTGTAATAACCCCGTAGATACTGTACCTAACAAAACATGACTCGTATTAGCTC | + |
| A_24_P15640 | UHRF1 | NaN | GCAGGGCCTGGTCACCCCCCGCACGTGGACACCCCAGGAGCCTTGGGGACCCCCAGGGTA | + |
| A_24_P175519 | TXN | thioredoxin | AAGTAGATGTGGATGACTGTCAGGATGTTGCTTCAGAGTGTGAAGTCAAATGCACGCCAA | + |
| A_24_P180225 | HTT | huntingtin | TGAACCTTTTCTGCCTGGTCGCCACAGACTTCTACAGACACCAGATAGAGGAGGAGCTCG | + |
| A_24_P191326 | MYL1 | myosin, light chain 1, alkali; skeletal, fast | AGAAAATTGAGTTTGAACAATTTCTGCCTATGATGCAAGCCATTTCCAACAACAAGGACC | + |
| A_24_P194845 | RAC1 | ras-related C3 botulinum toxin substrate 1 (rho family, small GTP binding protein Rac1) | CCAACGCATTTCCTGGAGAAGATATCCCTACTGCCTTTGACAATTATTCTGCCAATGTTA | + |
| A_24_P196851 | TLN1 | talin 1 | ACAAGTTTCTGCCTTCAGAGCTTCGAGATGAGCACTAAAGAAGCCTCTTCTATTTAATGC | + |
| A_24_P199500 | RNF2 | ring finger protein 2 | AAACTGCTTAGATTTTGTTGATGACATTAGATTAGTAGTTGCATTAAATAACTAAATTCC | + |
| A_24_P202527 | SMAD2 | SMAD family member 2 | TCAGTCTGTTAATCAGGGTTTTGAAGCCGTCTATCAGCTAACTAGAATGTGCACCATAAG | + |
| A_24_P2093 | XAB2 | XPA binding protein 2 | ACGTGGAAGGACTTTGAGGTCCGGCATGGCAATGAGGACACCATCAAGGAAATGCTGCGT | + |
| A_24_P211849 | TBX20 | T-box 20 | TTGCTCAACCTGAAGTCTGAAGAATTTAGAACTTTCATCTTTCCAGAAACAGTTTTTACG | + |
| A_24_P212851 | SMARCA4 | SWI/SNF related, matrix associated, actin dependent regulator of chromatin, subfamily a, member 4 | AGCCTCGGGTAAATTTGAGCTTCTTGATAGAATTCTTCCCAAACTCCGAGCAACCAACCA | + |
| A_24_P21715 | RAD9A | RAD9 homolog A (S. pombe) | CAAGTTTTCCTTGCTTTCCTGATACTCTTTGGCGCTGACTTGGAATTCTAAGAGCCTTGG | + |
| A_24_P22163 | DICER1 | dicer 1, ribonuclease type III | AGACAGTCTGGCAGGTGTACTATCCCATGATGCGGCCACTAATAGAAAAGTTTTCTGCAA | + |
| A_24_P225961 | DAG1 | dystroglycan 1 (dystrophin-associated glycoprotein 1) | TTGAGCATGACTTTTCTTGATGTCTGAAGCGTTATTTTGGGTACTTTTTAGGGAGGAATG | + |
| A_24_P226198 | RAD50 | RAD50 homolog (S. cerevisiae) | CCAAGTCACACGCTTTTGAATTATGCTTTGTAGAGTTTTGTCATTCAGAGTCAGCCAGGA | + |
| A_24_P231132 | ACVR2B | activin A receptor, type IIB | CTGTCACCAATGTGGACCTGCCCCCTAAAGAGTCAAGCATCTAAGCCCAGGACATGAGTG | + |
| A_24_P232809 | GSC | goosecoid homeobox | ACTGACGAGCAGCTCGAAGCTCTCGAGAACCTCTTCCAGGAGACCAAGTACCCGGACGTG | + |
| A_24_P235429 | ABCA1 | ATP-binding cassette, sub-family A (ABC1), member 1 | CCAAAGAGCCATGTGTCATGTAATACTGAACCACTTTGATATTGAGACATTAATTTGTAC | + |
| A_24_P245108 | USP7 | ubiquitin specific peptidase 7 (herpes virus-associated) | TTTTCCAGGAGAGAACTGTGTACAAATAAGGTGACTGGAGATGTGACCTGATGTGTCACG | + |
| A_24_P246125 | FLT1 | fms-related tyrosine kinase 1 (vascular endothelial growth factor/vascular permeability factor receptor) | GGTATTATTCTGTTTTGCACAGTTAGTTGTGAAAGAAAGCTGAGAAGAATGAAAATGCAG | - |
| A_24_P246467 | ATF2 | activating transcription factor 2 | AGTAGTCCACATACAGAAGCTATACAGCATAGTTCGGTCAGCACATCCAATGGAGTCAGT | + |
| A_24_P256380 | WLS | wntless homolog (Drosophila) | CTCTGGTATGACACATTTTCTGTGATCATTGTTAATTAGTGACATAGTAACATCTGTAGC | + |
| A_24_P261032 | RDH10 | retinol dehydrogenase 10 (all-trans) | TTGCAAGTTCCTTGGGATTGTTCAGTACTGCCGGAGTTGAGGATTACTGTGCCAGTAAAT | + |
| A_24_P278126 | NBN | nibrin | TGATGAAATGGAGTCATTTGAGTCTCTTAATAGCCATGTATCATAATTACCAAGTGAAGC | + |
| A_24_P279121 | MESP1 | mesoderm posterior 1 homolog (mouse) | ACGCAGGCTGAGGGGCAGGGGCAGGGGCGCGGGCTGGGCCTGGTATCCGCCGTCCGCGCC | + |
| A_24_P288116 | PIK3C3 | phosphoinositide-3-kinase, class 3 | CCATTGCTTTTAACAGGATTACCAAAGGCACACTCAGTAGTCAGTAAACACATTTCTAGG | + |
| A_24_P289265 | ABCA1 | ATP-binding cassette, sub-family A (ABC1), member 1 | GTGCTTCTGTACCAAAAGTGGAATTTCACGAGAGACATATTTTGAAACATTTCTCCTTTT | + |
| A_24_P296587 | DLX3 | distal-less homeobox 3 | GGTGGGTCTCAGACGTTTTTCCTATGGACTTATTTCTTCCATGTCCAGGACTTTGCACAA | + |
| A_24_P298939 | EP400 | E1A binding protein p400 | ACCTCGTCCCAAGACAGTTCTCAGGATACGCTGACAGAACAAATAACTCTGGAGAACCAG | + |
| A_24_P302584 | SOX11 | SRY (sex determining region Y)-box 11 | AGTACACTCCAATGTCTCTTTTGCAAGAGTTTTTCACAGAGGATTACATTTGTTCAAAAG | + |
| A_24_P303589 | CUL1 | cullin 1 | AGAAGCAGGAACAAGAAACCACACACAAAAACATCGAGGAAGACCGCAAACTACTGATTC | + |
| A_24_P305467 | GATAD2A | GATA zinc finger domain containing 2A | AAAGGACAGCAAACAATTTTATAATCCTTAAAGTGTAATAGACGGTTACACTAGTGCAGG | + |
| A_24_P30557 | TBX5 | T-box 5 | TTTTTTCCTTTTTTATAAACAGACCCTAATAAAGAGAACAGGGTAAGATGTGAGGCTGAG | + |
| A_24_P312915 | CDK11A/CDK11B | cyclin-dependent kinase 11B | AACAAGTTCCTGACCTACTTCCCCGGGAGGAGGATCAGCGCTGAGGACGGCCTCAAGCAT | + |
| A_24_P313597 | BECN1 | beclin 1, autophagy related | TTCAGAGATACCGACTTGTTCCTTACGGAAACCATTCATATCTGGAGTCTCTGACAGACA | + |
| A_24_P319635 | MCL1 | myeloid cell leukemia sequence 1 (BCL2-related) | ATCGAACCATTAGCAGAAAGTATCACAGACGTTCTCGTAAGGACAAAACGGGACTGGCTA | + |
| A_24_P324783 | ACVRL1 | activin A receptor type II-like 1 | CGAGGGATGAACAGTCCTGGTTCCGGGAGACTGAGATCTATAACACAGTATTGCTCAGAC | + |
| A_24_P330822 | HNF1B | HNF1 homeobox B | CCAGTATTCCCACACCTCCCGGTTTCCATCTGCAATGGTGGTCACAGATACCAGCAGCAT | + |
| A_24_P336754 | MCL1 | myeloid cell leukemia sequence 1 (BCL2-related) | ATGGCGCTCCCAGTGACTACTTTTTGACTTCTGTTTGTCTTACGCTTCTCTCAGGGAAAA | + |
| A_24_P336759 | MCL1 | myeloid cell leukemia sequence 1 (BCL2-related) | TATCGTTCTTGATCATAAGCCGCTTATTTATATCATGTATCTCTAAGGACCTAAAAGCAC | + |
| A_24_P336853 | PNO1 | partner of NOB1 homolog (S. cerevisiae) | GATTATCTCAGAAAAACCTCTCTGAATGATGACCCTTCCTTAATACTGGGTGATGTGTGA | + |
| A_24_P341897 | ACVR1B | activin A receptor, type IB | GGTGCCTCTTTTCAGTAGTGAGCAGCATCTAGTTTCCCTGGTGCCCTTCCCTGGAGGTCT | + |
| A_24_P345314 | TAL1 | T-cell acute lymphocytic leukemia 1 | ACCACCAACAATCGAGTGAAGAGGAGACCTTCCCCCTATGAGATGGAGATTACTGATGGT | + |
| A_24_P355772 | CDK11A/CDK11B | cyclin-dependent kinase 11B | GTGAGGAAGAAATGAGTGAAGATGAAGAACGAGAAAATGAAAACCACCTCTTGGTTGTTC | + |
| A_24_P361167 | CHD8 | chromodomain helicase DNA binding protein 8 | TCAGAAAGGGACTTCTCACTCATTGATGATCCTATGATGCCAGCTAACTCAGACTCCAGT | + |
| A_24_P365515 | FOXA2 | forkhead box A2 | TCCGGTTTCCACTACTGTGTAGACTCCTGCTTCTTCAAGCACCTGCAGATTCTGATTTTT | + |
| A_24_P365523 | FOXA2 | forkhead box A2 | CCTCCTACTACCAGGGGGTGTACTCCCGGCCCATTATGAACTCCTCTTAAGAAGACGACG | + |
| A_24_P367454 | ZEB2 | zinc finger E-box binding homeobox 2 | GATCCTAGAGGAGAAACAATCAGAAGAGCAGAAATGGTTATCCCTGTTTAAAATAAGCCC | + |
| A_24_P371962 | AMD1 | adenosylmethionine decarboxylase 1 | TCTCCTTTCACTGGTGTTGGACTTAAATCAGTTGAAATGTATTTCTGTACCACAATTTAC | + |
| A_24_P383080 | SRRT | serrate RNA effector molecule homolog (Arabidopsis) | GAGGAAGTGAAAAAGGAAGTCGCGTTTTTTAACAACTTCCTCACTGATGCTAAGCGCCCA | + |
| A_24_P384851 | SS18 | synovial sarcoma translocation, chromosome 18 | TCCAGGTCCTCAGTATCCTAACTACCCACAGGGACAAGGTCAGCAGTATGGAGGATATAG | + |
| A_24_P387179 | RPS6KB1 | ribosomal protein S6 kinase, 70kDa, polypeptide 1 | CTCAACCTTATCAAGGATTTTCATGTTGATGACTCGAAACTGACAGTATTAAGGGTAGGA | + |
| A_24_P408083 | DNMT1 | DNA (cytosine-5-)-methyltransferase 1 | TCTATGGAAGGCTCGAGTGGGACGGCTTCTTCAGCACAACCGTCACCAACCCCGAGCCCA | + |
| A_24_P42755 | FLT1 | fms-related tyrosine kinase 1 (vascular endothelial growth factor/vascular permeability factor receptor) | TGTGGCTGACTCTAGAATTTCTGGAATCTACATTTGCATAGCTTCCAATAAAGTTGGGAC | + |
| A_24_P47182 | VCL | vinculin | ATGGGGTTCAAGAGAGTAATGGGTTTCATATTTCTTATCACCACAGTAAGTTCCTACTAG | + |
| A_24_P497226 | RPS6KB1 | ribosomal protein S6 kinase, 70kDa, polypeptide 1 | TAACATTATAGCACAAGTATTATCTCAGTGGATTATCCGGAATAACATCTGAAAGATGGG | + |
| A_24_P521409 | RPS6KB1 | ribosomal protein S6 kinase, 70kDa, polypeptide 1 | CTCTTTTCCTTTAGGCAATGATAGTAAGAAATGCTAAAGATACAGCTCATACAAAAGCAG | + |
| A_24_P526177 | TDG | thymine-DNA glycosylase | TTTATACGTTTCCATTTCAACAACTGATGGCTGAAGCTCCTAATATGGCAGTTGTGAATG | + |
| A_24_P53519 | CHAF1A | chromatin assembly factor 1, subunit A (p150) | GTGCAGAGTTCTATATAGGATGCTGGATTAGTTCCTTTGATATTTGTAAAAATTCCCCCA | + |
| A_24_P56388 | HIF1A | hypoxia inducible factor 1, alpha subunit (basic helix-loop-helix transcription factor) | GTTGTCACAGTAAATATCTTGTTTTTTCTATGTACATTGTACAAATTTTTCATTCCTTTT | + |
| A_24_P576191 | FLT1 | fms-related tyrosine kinase 1 (vascular endothelial growth factor/vascular permeability factor receptor) | ATGGAATTAAGGCCATTGCAATGTATCATCTTTGTAGCATTGTCATCACTCCTAAGCTGC | + |
| A_24_P66239 | PELO | pelota homolog (Drosophila) | GGCAGCAATAATGTTCCTTGCAATAGGAATGAACAGAAGTCAACAGGAAATATTAAGAGA | + |
| A_24_P66672 | INTS1 | integrator complex subunit 1 | ATGAACTGCAACACGCACGGTTCCGAAGACATGGACGTCATCTCACACCTGATCAAGATC | + |
| A_24_P74981 | KDM1A | lysine (K)-specific demethylase 1A | TAGCTGTGAATACCCGCTCCACGAGTCAAACCTTTATTTATAAATGCGACGCAGTTCTCT | + |
| A_24_P753161 | BMPR2 | bone morphogenetic protein receptor, type II (serine/threonine kinase) | GTTCAAGCTCTAAATTGATGTGCTATATACTTAAAATCCTAGGAAGTTATCTGTAACCGG | + |
| A_24_P77219 | ARID1A | AT rich interactive domain 1A (SWI-like) | CATGGCGTGAACCGAACAGATGAAATGCTGCACACAGATCAGAGGGCCAACCACGAAGGC | + |
| A_24_P7887 | EP300 | E1A binding protein p300 | TGGCAGTATGTCGATGATATTTGGCTTATGTTCAATAATGCCTGGTTATATAACCGGAAA | + |
| A_24_P804263 | CSNK2B | casein kinase 2, beta polypeptide | AGATGTTGGAAAAGTACCAGCAAGGAGACTTTGGTTACTGTCCTCGTGTGTACTGTGAGA | + |
| A_24_P85574 | F2 | coagulation factor II (thrombin) | AGGAGGCCTTCGAGGCTCTGGAGTCCTCCACGGCTACGGATGTGTTCTGGGCCAAGTACA | + |
| A_24_P911545 | MLLT4 | myeloid/lymphoid or mixed-lineage leukemia (trithorax homolog, Drosophila); translocated to, 4 | ATATGAGGGGTCTTTTACCTTCCTACTTACTCAGTAAAGGTATTTTTGTATTCTCTGAGC | + |
| A_24_P911676 | SOX4 | SRY (sex determining region Y)-box 4 | GGCGGCTGGTTAATATCTCACACAGTTTAAAAAATCAGCCCCTAATTTCTCCATGTTTAC | + |
| A_24_P912587 | RAPGEF2 | Rap guanine nucleotide exchange factor (GEF) 2 | GCAAGCTTCCCATAGACAGTCTCGAAGGAGATTTAGAAAAATCAACCAGAAAGGTGAAAG | + |
| A_24_P912925 | PLK4 | polo-like kinase 4 | AATGCCACATGAAAAGCACTATACATTATGTGGAACTCCTAACTACATTTCACCAGAAAT | + |
| A_24_P91310 | PSMC4 | proteasome (prosome, macropain) 26S subunit, ATPase, 4 | GTTCAGAGGATCCTGCTGGAGCTGCTGAATCAGATGGATGGATTTGATCAGAATGTCAAT | + |
| A_24_P913900 | SLC8A1 | solute carrier family 8 (sodium/calcium exchanger), member 1 | CTTGTGCTTGAGGAACCAAAATGGATAAGAAGAGGAATGAAAGGTGGCTTCACAATCACA | + |
| A_24_P913947 | G6PD | glucose-6-phosphate dehydrogenase | CTTGCAGCTTGTCACTAGGAAGCCTTGTTTGGGGTCCCCATGCCCTTGAACCAGGTGAAC | + |
| A_24_P917744 | DCTN1 | dynactin 1 | AAGGGGAACAGGAATTCTGGGTTCCTGAAGTAGGAATACAGTGATTCACTGAAGGGAGAA | + |
| A_24_P922475 | ATF7 | activating transcription factor 7 | TGGTTAAAACACAAAATCATGGGCTTAGTAGAGTAAAACCAGTAAAACCAGATAGTAGTC | + |
| A_24_P927182 | POU2F1 | POU class 2 homeobox 1 | CTGCCTTTTTAATTACCAAACTACTCTCAGTTTTCAATGAATCAGTTCAAAGAAAGAATG | + |
| A_24_P927287 | KRAS | v-Ki-ras2 Kirsten rat sarcoma viral oncogene homolog | TCATTTCAAATATGTAGTATCTTCAGAATATTTGAGAAGGATTTGTATTATATAATTGAA | - |
| A_24_P92952 | ARID1A | AT rich interactive domain 1A (SWI-like) | ATCACCGTTGATGAACTCATTGGTTTCACAAGTCATTTGTGATGTACTGTTTTTGATTGG | + |
| A_24_P932785 | GATA4 | GATA binding protein 4 | AGGGTTGGAAATAACCGTTGTGGTAGGTTCCATGCAGTGTTTCCATCGGATGTCAGACGG | + |
| A_24_P935919 | RAPGEF2 | Rap guanine nucleotide exchange factor (GEF) 2 | GTACTGCTAGGTCTTTTGCAAGCACTTTATCATTAACATGTGACTTATGTTTTATTCCAG | + |
| A_24_P937582 | RNASEH1 | ribonuclease H1 | CACCATTGACTCATTTCTGTGTGTTCAGGTCTCATAACCAGTCTATAGTCAGTGTCATCT | + |
| A_24_P941441 | GNA13 | guanine nucleotide binding protein (G protein), alpha 13 | CGCCTTAATACCAGAAATGATTAGAAGTGCTGATTTAGATTCAACAAATACCATATGTCC | + |
| A_24_P94402 | MYCN | v-myc myelocytomatosis viral related oncogene, neuroblastoma derived (avian) | GTTAATCTCTGTTATGTACTGTACTAATTCTTACACTGCCTGTATACTTTAGTATGACGC | + |
| A_24_P945113 | ACVRL1 | activin A receptor type II-like 1 | GGTGTTCCAGGGTCGAAATTACACTTCTCGTACCTGGAGACGCTGTTTGTGGGAGCACTG | + |
| A_24_P96709 | CDK11A/CDK11B | cyclin-dependent kinase 11A | AGAATTAAATCATTTTCCTTGTTGTGGAGGAAAGAGCTGTGTTTTCTCCGTGACTTGCCA | + |
| A_24_P97374 | EOMES | eomesodermin | ATTTGTAAATTCTTAAGCAAATAGAAGCCGAGTGTTAAGGTGTTTTGCTTCTGAAAGAGG | + |
| A_24_P97931 | CSNK2B | casein kinase 2, beta polypeptide | CAACCAGTTTGTGCCCAGGCTCTACGGTTTCAAGATCCATCCGATGGCCTACCAGCTGCA | + |
| A_32_P101235 | UHRF1 | ubiquitin-like with PHD and ring finger domains 1 | AGACGTTCCAGTGTATCTGCTGTCAGGAGCTGGTGTTCCGGCCCATCACGACCGTGTGCC | + |
| A_32_P109002 | SMAD2 | SMAD family member 2 | GTGCCCTTGTCAATTTAAGACTAAGACTTTGAAGGTAAAACAAACAAACAAACATCAGTC | + |
| A_32_P110372 | SLC8A1 | solute carrier family 8 (sodium/calcium exchanger), member 1 | TGGAGGGGAGGATTTTGAGGACACTTGTGGAGAGCTCGAATTCCAGAATGATGAAATTGT | + |
| A_32_P113784 | SOX11 | SRY (sex determining region Y)-box 11 | GCGTTTGTACAGTCTGCATTTTTTCAAGCTCCCTGCAGTTTTGTTAAGAATCGGATGCAT | + |
| A_32_P123990 | PDCD2 | programmed cell death 2 | TCCTTATAGGCAGAATCTGTAAGTACGTTATGACTGCTAATGACTTTTAAAGCAAACATG | + |
| A_32_P12580 | SMAD2 | SMAD family member 2 | TTCATAGTAAAAGGAAACCAATTGTTGCAGATTTCTTTTCTTGTGAGGAAATACATGGCC | + |
| A_32_P129689 | DICER1 | dicer 1, ribonuclease type III | CCTGCTGCTGCAGTGAATTCTTAGTGCATCTATAAAATTTAAATTGTCTGTGGTTGATGT | + |
| A_32_P134209 | ACVR2B | activin A receptor, type IIB | TGGCCTAAAGCAGACATCCATGTAATTACAGTTGCAAAATGAAAACATTTTGGAAAGAAC | + |
| A_32_P135985 | TDGF1 | teratocarcinoma-derived growth factor 1 | AGCTGGCATCTGCCTTTCTATACAAAGCTACTATTAATCGACATTGACCTATTTCCAGAA | + |
| A_32_P142077 | POU2F1 | POU class 2 homeobox 1 | TTCTTCCTTTTTTGTCTTAATTATTAATTCAGGGGTGTTTGTCCACTGTTGTCAAAGGAG | + |
| A_32_P147078 | SLC8A1 | solute carrier family 8 (sodium/calcium exchanger), member 1 | GGGGGGCGGGAAATGCACTAATTGTGCTCTTCCTTATAAATGGTACATATTACTGACACA | + |
| A_32_P149416 | TXNRD1 | thioredoxin reductase 1 | CTCTAGCCATGACTTGCTTTTGGACAAAAATCAACTGCTAACGTTTTTCATCTCTAATAT | + |
| A_32_P155631 | SDHD | succinate dehydrogenase complex, subunit D, integral membrane protein | GAGGAATTATATCTAAGTTGTGAGACTGAGTTCTGTATTCTGGTGAGTTAATGGGGTTGC | + |
| A_32_P188226 | ACVRL1 | activin A receptor type II-like 1 | ATGTTGTCGTGTCTGAGCAACACTGTGTTATAGATCTCAGTCTCCCGGAACCAGGACTGT | + |
| A_32_P199884 | HORMAD1 | HORMA domain containing 1 | AGGTCTAAAGAAAGTCCAGATCTTTCTATTTCTCATTCTCAGGTTGAGCAGTTAGTCAAT | + |
| A_32_P213678 | SMARCA5 | SWI/SNF related, matrix associated, actin dependent regulator of chromatin, subfamily a, member 5 | GGTGTGTGGCTCTTATCTCTAGGACACCAATATTTAATGTTGCATATCATGTATCTCCAG | + |
| A_32_P215318 | ACACA | acetyl-CoA carboxylase alpha | AGCACTCTCGATTCATAATAGGTTCTGTGTCTGAAGATAACTCAGAGGATGAGATCAGCA | + |
| A_32_P217709 | RAC1 | ras-related C3 botulinum toxin substrate 1 (rho family, small GTP binding protein Rac1) | GTTACACAACCAATGCATTTCCTGGAGAATATATCCCTACTGTCTTTGACAATTATTCTG | + |
| A_32_P218249 | TDG | thymine-DNA glycosylase | GACGTTTTGTGCGGGTGCTTTGAAGTGCCTTGCATCAGGGATTAGGAGCAATTAAGTTAT | + |
| A_32_P25050 | RDH10 | retinol dehydrogenase 10 (all-trans) | GGCATATGTATGGAAGGGTGTAAAGATTCTTTTGAAAGGTTTATTCACATTGTAGAACAG | + |
| A_32_P28365 | NASP | nuclear autoantigenic sperm protein (histone-binding) | TGAGGAACTAAAGGAACTGCTACCCGAAATTAGAGAGAAGATAGAAGATGCAAAGGAGTC | + |
| A_32_P56154 | UHRF1 | ubiquitin-like with PHD and ring finger domains 1 | AACACTACCTGTTTAAGTTAAGCCCACTAAGAACAAAATCAGTGCCGATGAACGAGCCCT | + |
| A_32_P59486 | SDHD | succinate dehydrogenase complex, subunit D, integral membrane protein | TTAAGCTCACAATAAGGAAGAAATAACAGATAAGTCCATTGGTGGACAGCCTTCTTCTCT | + |
| A_32_P60185 | SDHD | succinate dehydrogenase complex, subunit D, integral membrane protein | AATAAGGAAGAAATAACAGATAAGTCCATTGGTGGACAGCCTTCTTCTCTTAATCACAAG | + |
| A_32_P65157 | ACACA | acetyl-CoA carboxylase alpha | GATGTGGTGGTCTACTCTGATGTCAATCTTGAGGGCTAGGTATGTCCCAAGATCTCTTAT | + |
| A_32_P926336 | UHRF1 | ubiquitin-like with PHD and ring finger domains 1 | TGAGTTCCCACTTAGCTTTTTACAGTTTCTTCGTCACTCTATTAAGTCTCCTCTATCTGG | + |
| A_32_P93187 | SMARCA5 | SWI/SNF related, matrix associated, actin dependent regulator of chromatin, subfamily a, member 5 | ATAGGGGCCAGAGTTTCTGCCTCCAAAATTTCCTCCCTTCATGGGTCCAAAATTTGAAGA | + |
| A_32_P96752 | SOX4 | SRY (sex determining region Y)-box 4 | GTTAAAACTCTAGCCCTTCAGTGAAGGAGACGTAAAATGGCGTGGGTAACAACAACTACC | + |
|  |  |  |  |  |
| **DNA Methylation** | | | | |
| A_23_P104323 | MGMT | O-6-methylguanine-DNA methyltransferase | TCTTCACCATCCCGTTTTCCAGCAAGAGTCGTTCACCAGACAGGTGTTATGGAAGCTGCT | + |
| A_23_P106194 | FOS | FBJ murine osteosarcoma viral oncogene homolog | AGAGGGTTCCTGTAGACCTAGGGAGGACCTTATCTGTGCGTGAAACACACCAGGCTGTGG | + |
| A_23_P107507 | CBX1 | chromobox homolog 1 | CAAGCGCAAAGCTGATTCTGATTCTGAAGATAAGGGAGAGGAGAGCAAACCAAAGAAGAA | + |
| A_23_P112950 | ATF7IP | activating transcription factor 7 interacting protein | TTTTGGGCCTTTCTGTGATCCTCAGTCAACAGATGTGATCTCTTCTACCCAGAGCAGTTA | + |
| A_23_P115636 | TRDMT1 | tRNA aspartic acid methyltransferase 1 | AAGGGACAGGGTCTGTGTTACAGACTGCAGAGGATGTGCAGGTTGAGAATATCTACAAAT | + |
| A_23_P117515 | ARID4A | AT rich interactive domain 4A (RBP1-like) | TTACAACCACAGAAAGCACTCAACTGGTTTGACATTGCTAAGTATATCCTGTATACTTTT | + |
| A_23_P12816 | HELLS | helicase, lymphoid-specific | TAGATGTCATAGAATTGGTCAGACAAAGCCAGTTGTTGTTTATCGCCTTGTTACAGCAAA | + |
| A_23_P131020 | GATAD2A | GATA zinc finger domain containing 2A | AAAGGATCAGGTCTGCTTTTAGTTTCATTTTTGTTTCTTTCCCGTCCCACTCTTTAAAAA | + |
| A_23_P133728 | GCM1 | glial cells missing homolog 1 (Drosophila) | CAAAACTGGCTGTCCCCCATTATGGCCAAATCCAGCGGGTAATCTTTATGAAGAGAAAGT | + |
| A_23_P136874 | ATRX | alpha thalassemia/mental retardation syndrome X-linked | ACCTCATGATGCCAAAGCCCCCAAATTTGATCATGAATCCTTCTAACTACCAGCAGATTG | + |
| A_23_P143774 | MOV10L1 | Mov10l1, Moloney leukemia virus 10-like 1, homolog (mouse) | AATACAGTATTACAAACGGTGTTTACATGGGATGCGATTTACCTCCTGCACTGCAGTCTC | + |
| A_23_P150609 | IGF2 | insulin-like growth factor 2 (somatomedin A) | CTCAACTCAGCTCCTTTAACGCTAATATTTCCGGCAAAATCCCATGCTTGGGTTTTGTCT | + |
| A_23_P154500 | DNMT3A | DNA (cytosine-5-)-methyltransferase 3 alpha | AGGGAGACGAGAACACCACACAAGACATTTTTCTACAGTATTTCAGGTGCCTACCACACA | + |
| A_23_P156953 | IGF2R | insulin-like growth factor 2 receptor | GCTTCTATAATTTTGCCTTTAACAGAAACTTTCAAAAGGGAAGAGTTTTTGTGATGGGGG | + |
| A_23_P17673 | DNMT3L | DNA (cytosine-5-)-methyltransferase 3-like | TAAGAGAATATTTCAAGTATTTTTCAACAGAACTCACTTCCTCTTTATAAATGAGTCACT | + |
| A_23_P201951 | ARID4B | AT rich interactive domain 4B (RBP1-like) | ATGTTTACAGGTTTGAATTAGGCTAAAAGGTCTTGCAGTGGCTTTTCATGGCCCTTCAAA | + |
| A_23_P203841 | BAZ2A | bromodomain adjacent to zinc finger domain, 2A | GCAATTTTCCCTTGGTATAAGATGTGCTAGATTAATTTCATTGTGAGGTGGATGGGGGAG | + |
| A_23_P204579 | TDG | thymine-DNA glycosylase | ATGACGGACATCCACTAGAGATGGGTTTGAGGATTTTCCAAGCGTGTAATAATGATGTTT | + |
| A_23_P204850 | RB1 | retinoblastoma 1 | GTGCCAGAATTTTAGGAACTTCAGAGATCGTGTATTGAGATTTCTTAAATAATGCTTCAG | + |
| A_23_P208090 | MBD1 | methyl-CpG binding domain protein 1 | CATGGAATTTAATGCTGAATATATAGAATTCAGAAAATTGTTGGGAGGACAGCCCTTTTG | + |
| A_23_P208880 | UHRF1 | ubiquitin-like with PHD and ring finger domains 1 | TGTATTAGGGAAGAATGAGACAATTTTGTGTAGGCTTTTTCTAAAGTCCAGTACTTTGTC | + |
| A_23_P20894 | EHMT1 | euchromatic histone-lysine N-methyltransferase 1 | TGATTTCAGACTCAGAAGCCGACGTTCGAGAGGAAGATTCTTACCTCTTTGATCTCGACA | + |
| A_23_P215956 | MYC | v-myc myelocytomatosis viral oncogene homolog (avian) | TTCAAATGCATGATCAAATGCAACCTCACAACCTTGGCTGAGTCTTGAGACTGAAAGATT | + |
| A_23_P217120 | EHMT1 | euchromatic histone-lysine N-methyltransferase 1 | TTGTAGCGTGAATAAATTTGCCATCACCTTTTGTGTGGTGGCCTGGCAGGTCATATACTT | + |
| A_23_P2355 | CBX5 | chromobox homolog 5 | CTTTGAGAGAGGACTGGAACCAGAAAAGATCATTGGGGCAACAGATTCCTGTGGTGATTT | + |
| A_23_P252335 | MIS18A | MIS18 kinetochore protein homolog A (S. pombe) | GCCGAATCCAAATTGTCCTTTGCCACTTGTAAAAGCTGAACTCTAGTCTGTGTCCTCCAT | + |
| A_23_P28733 | RBL1 | retinoblastoma-like 1 (p107) | ATGCAGAATCCCCTGCCAAACGCGTCTGTCAAGAAAATGATGACGTTTTACTGAAACGAC | + |
| A_23_P28953 | DNMT3B | DNA (cytosine-5-)-methyltransferase 3 beta | AACTGTGCCTTGTTTCAACAGTTTTTGCTAATTTTTAGGCTGAAAGATGACGGATGCCTA | + |
| A_23_P31315 | CBX3 | chromobox homolog 3 | GCGTTGGAAGAGTTGTTGGGGGTTTTTTGCATCCATAGCACTGGTTACTTTGAACAAATA | + |
| A_23_P334021 | IGF2R | insulin-like growth factor 2 receptor | GCCTTAATTTGCACAGTGTGTGACTTACAGAAACTGCATGAAAAATCATGGGCCAGAGCC | + |
| A_23_P3371 | RASGRF1 | Ras protein-specific guanine nucleotide-releasing factor 1 | TCTTTTGTAATGGATGAAGAAAGCCTCTACGAGTCTTCTCTCCGAATAGAACCAAAACTC | + |
| A_23_P3379 | RASGRF1 | Ras protein-specific guanine nucleotide-releasing factor 1 | AGGACCCCTTATATCATGAAAACCACTAAGCACTTCAATGACATCAGTAACTTGATTGCT | + |
| A_23_P348323 | PCGF2 | polycomb group ring finger 2 | AACTGGCTGCATATAGGAGTCAGTTAGAATTGTTTCTTTCTCTCCCCGTTTCTCTCCCCA | + |
| A_23_P357717 | TCL1A | T-cell leukemia/lymphoma 1A | TTTCCCCCCTTTATAGATGGTCACGCACCTGGGTGTTACAAAGTTGTATGTGGCATGAAT | + |
| A_23_P360626 | PLD6 | phospholipase D family, member 6 | TATATAGATTGGAAGTCACCAGAAATTGTTTTAGTTTGTGAATTCCCCCAGTGTATGTTC | + |
| A_23_P388780 | DMAP1 | DNA methyltransferase 1 associated protein 1 | GATACCAGTATTTGATGCTGGGCACGAACGACGGCGGAAGGAACAGCTTGAGCGTCTCTA | + |
| A_23_P420373 | DNMT3A | DNA (cytosine-5-)-methyltransferase 3 alpha | CAAAAAGTCTTCCTTCCAAGCGTGTATGATGAAATGAGTAAATTGATTAATTGGCGTAAC | + |
| A_23_P421379 | IGF2 | insulin-like growth factor 2 (somatomedin A) | TGCTTCCGGACAACTTCCCCAGATACCCCGTGGGCAAGTTCTTCCAATATGACACCTGGA | + |
| A_23_P48278 | ATF7IP | activating transcription factor 7 interacting protein | CCTTCTTTTATGGTCAAACTGAAAAACATTATTCCTCTATGAAACTTTCTAAGCCTGTGG | + |
| A_23_P586 | DMAP1 | DNA methyltransferase 1 associated protein 1 | CTCTGCTGAGCCGGCAGTGACTGAACCCGGACTTGGTCCTGACCCCAAGGACACCATCAT | + |
| A_23_P59210 | CDKN1A | cyclin-dependent kinase inhibitor 1A (p21, Cip1) | CATCCCTCCCCAGTTCATTGCACTTTGATTAGCAGCGGAACAAGGAGTCAGACATTTTAA | + |
| A_23_P59285 | GCM2 | glial cells missing homolog 2 (Drosophila) | GCAGGAGGCAGGGAAATGTGAAATGGCAATGATCTCTTATTGAGTTGGGAGATTCACCTT | + |
| A_23_P64217 | MLL | myeloid/lymphoid or mixed-lineage leukemia (trithorax homolog, Drosophila) | ACTTGCATGGAGTTGGTGCTTAAATGTGAGTTGATTTTGCTTTTTTAAAAGATACAGCAG | + |
| A_23_P70213 | APC | adenomatous polyposis coli | AACAATTGAAGAAGACTGTTGCCACTTAACCATTCCATGCGTTGGCACTTATCTATTCCT | + |
| A_23_P71213 | ASZ1 | ankyrin repeat, SAM and basic leucine zipper domain containing 1 | ACAATTTGGAGAGCTATCTGAAGAGACAAAGTTGGAAATCAGTGGTGATGAGTTCCTCAA | + |
| A_23_P77066 | SNRPN | small nuclear ribonucleoprotein polypeptide N | AAGATGCTGCAGCACATTGACTATAGAATGAGATGTATCCTGCAAGATGGCCGAATCTTC | + |
| A_23_P77430 | PRMT7 | protein arginine methyltransferase 7 | GTGGAGTTTCACCCCGACACAGGCGACATCATCATGGAGTTCAGGCATGCAGATACCCCA | + |
| A_23_P77437 | PRMT7 | protein arginine methyltransferase 7 | AGAAAAATCTTCAAGGCTAACCACTTGGAAGATAAAATTAACATCATAGAGAAACGGCCG | + |
| A_23_P91727 | PICK1 | protein interacting with PRKCA 1 | CCTGTCGTACTGCCTGAAGGTGAAGGAGATGGATGACGAGGAATACAGCTGCATTGCCCT | + |
| A_23_P92154 | MBD4 | methyl-CpG binding domain protein 4 | TCATGACTGGCTTTGGGAAAATCATGAAAAATTAAGTCTATCTTAAACTCTGCAGCTTTC | + |
| A_23_P97835 | TDRD1 | tudor domain containing 1 | CTGGTAAAAAGTTAAGTAAGTTAAATCGTATGTTTTCGCCTCTTCTGTGATCACCAATAG | + |
| A_24_P102636 | RB1 | retinoblastoma 1 | ACCTCCCATGTTGCTCAAAGAACCATATAAAACAGCTGTTATACCCATTAATGGTTCACC | + |
| A_24_P127812 | MLL | myeloid/lymphoid or mixed-lineage leukemia (trithorax homolog, Drosophila) | AAGTTTGTGTATTGCCAAGTCTGTTGTGAGCCCTTCCACAAGTTTTGTTTAGAGGAGAAC | + |
| A_24_P128041 | ATRX | alpha thalassemia/mental retardation syndrome X-linked | AAATCCAAGTCTTCAGGATCGTCACGATCAAAGAGGAAACCTTCAATTGTAAACAAAAAT | + |
| A_24_P136402 | MLL | myeloid/lymphoid or mixed-lineage leukemia (trithorax homolog, Drosophila) | TCCATTCCAAATGTGAGAATCTTTCAGATGAGATGTATGAGATTCTATCTAATCTGCCAG | + |
| A_24_P15640 | UHRF1 | NaN | GCAGGGCCTGGTCACCCCCCGCACGTGGACACCCCAGGAGCCTTGGGGACCCCCAGGGTA | + |
| A_24_P170613 | MLL | myeloid/lymphoid or mixed-lineage leukemia (trithorax homolog, Drosophila) | AGAGAGCGAGAGCAGTTCAAGTGACAGCGAGTTCAAGTGACAGCGAAGAAAATGAGCCCC | + |
| A_24_P178011 | MYC | v-myc myelocytomatosis viral oncogene homolog (avian) | CGCGCGCCCATTAATACCCTTCTTTCCTCCACTCTCCCTGGGACTCTTGATCAAAGCGCG | + |
| A_24_P185347 | MBD1 | methyl-CpG binding domain protein 1 | GCAGTAGACCCAGGCCTGCCTTCTGTGAAGCAAGAGCCACCTGACCCAGAGGAGGACAAG | + |
| A_24_P196298 | MLL | myeloid/lymphoid or mixed-lineage leukemia (trithorax homolog, Drosophila) | GCACTTTGAACATCCTCAGCACTCTCTCCAATGGCAATAGTTCTAAGCAAAAAATTCCAG | + |
| A_24_P196499 | ARID4B | AT rich interactive domain 4B (RBP1-like) | AAGTGGAAATTCAAATCGGTACCTTCCCAAAGTATTAGTGCCTTTCGATGGTGCCATAGC | + |
| A_24_P196704 | MLL5 | myeloid/lymphoid or mixed-lineage leukemia 5 (trithorax homolog, Drosophila) | ATACTGCATTTCAGCATACTCCAACATCAATTACTTTAACTGCTTCAAGAGTTTCCAAAG | + |
| A_24_P225308 | ARID4B | AT rich interactive domain 4B (RBP1-like) | GTTGAAAATGGTTTCAAGTTATTCAAATTTGTACAGGACTGTAAAGATTTGTTGACAGCA | + |
| A_24_P231010 | MOV10L1 | Mov10l1, Moloney leukemia virus 10-like 1, homolog (mouse) | GACAGGAAGTGATTGCAGTTGTGGAAGAAAATAAAGTGTCCAATGGACTGAAAGCAATCA | + |
| A_24_P252705 | TRDMT1 | tRNA aspartic acid methyltransferase 1 | CAAACTGGTAGACAGAATGTAGCTTAGTGGAATTCTTAAATTATGGGAATGTATTCACAG | + |
| A_24_P276102 | RBL1 | retinoblastoma-like 1 (p107) | TTTTACAATACAATATATGTAGGAAGAGTGAAGTCATTTGCACTGAAATACGACTTGGCG | + |
| A_24_P281913 | MLL | myeloid/lymphoid or mixed-lineage leukemia (trithorax homolog, Drosophila) | ATGAGAAAATGTCAGAATCTACAATGGATGCCTTCCAAAGCCTACCTGCGAGAAGCTGGA | + |
| A_24_P296568 | CBX1 | chromobox homolog 1 | CTTGCATCTAGAGCTACGTTGTAAAATTCTTTTAGGCATGTGTTAGATTTCTGTGTAAAC | + |
| A_24_P298174 | CBX1 | chromobox homolog 1 | AGCACATGAGATAGACAAATCAGAGGGAGGCAAGCACAAAGCTGATTCTGATTCTGAAGA | + |
| A_24_P305467 | GATAD2A | GATA zinc finger domain containing 2A | AAAGGACAGCAAACAATTTTATAATCCTTAAAGTGTAATAGACGGTTACACTAGTGCAGG | + |
| A_24_P330309 | ARID4A | AT rich interactive domain 4A (RBP1-like) | TTACAACCACAGAAAGCACTCAACTGGTTTGACATTGCTAAGTATATCCTGTATACTTTT | + |
| A_24_P348660 | ATRX | alpha thalassemia/mental retardation syndrome X-linked | GGGTTAATTGGAAATCAGAGTTTGAAATAAAACTTGGACCACTTTGTATACACTCTTCTC | + |
| A_24_P376322 | MBD1 | methyl-CpG binding domain protein 1 | GTTGTGCAAAGACTGTCGAGCACAGAGAATTGCCTTCAACCGGGAACAGAGAATGTTTAA | + |
| A_24_P38363 | MYC | v-myc myelocytomatosis viral oncogene homolog (avian) | CGGTTTTCGGGGCTTTATCTAACTCGCTGTAGTAATTCCAGCGAGAGGCAGAGGGAGCGA | + |
| A_24_P387437 | CTCFL | CCCTC-binding factor (zinc finger protein)-like | AAATCAGATAAGGCAAATGTGTACCTGTAAGGAATTTGTACTTTTTCATAATGCCCAGTG | - |
| A_24_P408083 | DNMT1 | DNA (cytosine-5-)-methyltransferase 1 | TCTATGGAAGGCTCGAGTGGGACGGCTTCTTCAGCACAACCGTCACCAACCCCGAGCCCA | + |
| A_24_P411863 | MLL5 | myeloid/lymphoid or mixed-lineage leukemia 5 (trithorax homolog, Drosophila) | AGGACCAAACAGTATTCCAACACCTACTGCTTCAGGGTTCTGTCCTCATCCTGGCTCTGT | + |
| A_24_P500584 | XIST | X (inactive)-specific transcript (non-protein coding) | AATATTTCAATGCCTATTCTCTGCAAGGTACTATGTTTCGTAAATTAAATAGGTCTGGCC | + |
| A_24_P526177 | TDG | thymine-DNA glycosylase | TTTATACGTTTCCATTTCAACAACTGATGGCTGAAGCTCCTAATATGGCAGTTGTGAATG | + |
| A_24_P52697 | H19 | H19, imprinted maternally expressed transcript (non-protein coding) | CGTCCCTTCTGAATTTAATTTGCACTAAGTCATTTGCACTGGTTGGAGTTGTGGAGACGG | + |
| A_24_P601643 | SNRPN | small nuclear ribonucleoprotein polypeptide N | GCATAGTAAATTCTAGTTAAAAATACTAAACTACTAAGTTTTAGTTAACATACATGCTCA | + |
| A_24_P620621 | CBX5 | chromobox homolog 5 | TGGACTTGCATGTTTGAGAAGGCTTCCAACCACATCTTCACTCAGACTCTTGGAATTCCT | + |
| A_24_P664995 | CBX5 | chromobox homolog 5 | TAAGCATTTATGTGTTTCCATAACTGACATCTGATGCAGACCTCATTCTCTCCCCCTCTT | + |
| A_24_P89457 | CDKN1A | cyclin-dependent kinase inhibitor 1A (p21, Cip1) | TCTGCTGCAGGGGACAGCAGAGGAAGACCATGTGGACCTGTCACTGTCTTGTACCCTTGT | + |
| A_24_P918740 | APC | adenomatous polyposis coli | GCTCAACTTCAGAATCTCACTAAAAGAATAGATAGTCCTCCTTTAACTGAAAATTTTTCC | + |
| A_24_P922909 | MLL | NaN | AAGAAAAAGCAGCCTCCACCACCAGAATCAGGTCTAGAGCGGAAGCTGGAGTGGTGGCCT | + |
| A_24_P923757 | ATF7IP | activating transcription factor 7 interacting protein | TGAGCTGACTTCTGAATCAACCTTTGATCGTACCTTTGAACCAAAGTCTGTACCAGTTGG | + |
| A_24_P931874 | MLL | NaN | GGAGTCCACAGGATCAGAGTGGACTTTAAGGATTCTGTTTCACTGAGGCCATCTATCCGA | + |
| A_24_P936758 | IGF2 | insulin-like growth factor 2 (somatomedin A) | CCAATTACATTTCATTTGCATGGATTTTGGTTTTCATGCTCTGTCCTCCCCTCCTTTGGT | + |
| A_32_P101235 | UHRF1 | ubiquitin-like with PHD and ring finger domains 1 | AGACGTTCCAGTGTATCTGCTGTCAGGAGCTGGTGTTCCGGCCCATCACGACCGTGTGCC | + |
| A_32_P200934 | HELLS | helicase, lymphoid-specific | TCAGTGTGTATCCACCACATTTAACTTATTCACTTACAGAACTATGCAAGAATTTCTCTG | + |
| A_32_P208350 | TDRD9 | tudor domain containing 9 | CTCTTTGGGTGATAGTCAGAGAGTGGTGTTTTTGTTCAGGTGGGAAGGATTGGAAACTCT | + |
| A_32_P21579 | TRDMT1 | tRNA aspartic acid methyltransferase 1 | TGGAATGTGATACTCCTTGTGTAGTAGAAATGAGTTGACTTTCATATTTAAGAGCTGATC | + |
| A_32_P218249 | TDG | thymine-DNA glycosylase | GACGTTTTGTGCGGGTGCTTTGAAGTGCCTTGCATCAGGGATTAGGAGCAATTAAGTTAT | + |
| A_32_P23333 | SNRPN | NaN | TGTCCTTTAATGAAAATGCTCTTGACACCAATGCATCCTAGCATCACAGCTTCAGGAAGC | + |
| A_32_P42989 | IGF2 | insulin-like growth factor 2 (somatomedin A) | TGGGCAGGTAATTTGGGGTGCCTCGAAGCGTTTTGGATCTCAGGCCAATGTGGGTTCCAC | + |
| A_32_P56154 | UHRF1 | ubiquitin-like with PHD and ring finger domains 1 | AACACTACCTGTTTAAGTTAAGCCCACTAAGAACAAAATCAGTGCCGATGAACGAGCCCT | + |
| A_32_P56661 | IGF2 | insulin-like growth factor 2 (somatomedin A) | GGGGGAGGGTATGTGAAGGGTGTTTAAAGCCAATCGATTTTGTACATGTTTGAAGATGCT | + |
| A_32_P60687 | MYC | v-myc myelocytomatosis viral oncogene homolog (avian) | TCCGTAGCTGTTCAAGTTTGTGTTTCAACTGTTCTCGTCGTTTCCGCAACAAGTCCTCTT | + |
| A_32_P7521 | MIS18A | MIS18 kinetochore protein homolog A (S. pombe) | ACGCAACCATTTTCCTTTTCACGTTTGGATAGCTTCTGTTCCTTATCCACAGAAACATTA | + |
| A_32_P926336 | UHRF1 | ubiquitin-like with PHD and ring finger domains 1 | TGAGTTCCCACTTAGCTTTTTACAGTTTCTTCGTCACTCTATTAAGTCTCCTCTATCTGG | + |
| A_32_P98732 | GCM1 | glial cells missing homolog 1 (Drosophila) | ATGTTAAATTGCCATATGAAGAGAAACACTAGTGATGTGCGTGGTCTCCTTGCCTCTTGT | + |
|  |  |  |  |  |
| **Arsenic related genes (Fei et al., 2013)** | | | | |
| A_23_P106362 | AQP9 | aquaporin 9 | TCTACATCAAGGGATGCACCTTCAGTCAAACTGTCAAAAAGCCCAGAATTCCCAAAGGCA | + |
| A_23_P120883 | HMOX1 | heme oxygenase (decycling) 1 | TGGGGAGGGAGGTGTTTAACGGCACTGTGGCCTTGGTCTAACTTTTGTGTGAAATAATAA | + |
| A_23_P12643 | AS3MT | arsenic (+3 oxidation state) methyltransferase | GGAAGGTGAAATTGTTGAAGTGGATGAAGAAACAGCAGCTATCTTGAAGAATTCAAGATT | + |
| A_23_P128855 | SLC39A2 | solute carrier family 39 (zinc transporter), member 2 | TGGACCCCAGGTTTCCTTTACATGAGATCCCATTTCTCACCCTGGACTAAGACAAAGATA | + |
| A_23_P138541 | AKR1C3 | aldo-keto reductase family 1, member C3 (3-alpha hydroxysteroid dehydrogenase, type II) | TTTTGAGTTCCAGTTGACTGCAGAGGACATGAAAGCCATAGATGGCCTAGACAGAAATCT | + |
| A_23_P50096 | TYMS | thymidylate synthetase | ACATGTATGTGCATTTCAATCCCACGTACTTATAAAGAAGGTTGGTGAATTTCACAAGCT | + |
| A_23_P5761 | NFE2L2 | nuclear factor (erythroid-derived 2)-like 2 | CAGTAGTTTCACTTTAACTGTAAACAATTTCTTAGGACACCATTTGGGCTAGTTTCTGTG | + |
| A_23_P94338 | ENPP2 | ectonucleotide pyrophosphatase/phosphodiesterase 2 | GGCCTGATGTCCGTGTTTCTCCGAGTTTCAGTCAGAACTGTTTGGCCTACAAAAATGATA | + |
| A_24_P397817 | LEP | leptin | GCTGAAGGGACCTTGAAGGGTAAAGAAGTTTGATATTAAAGGAGTTAAGAGTAGCAAGTT | + |
|  |  |  |  |  |
| **Process: Oxidative Stress** | | | | |
| A_23_P100501 | HMOX2 | heme oxygenase (decycling) 2 | CTAGCTGCTGGACTCTTGGCCTGGTACTACATGTGAAGCACCCATCATGCCACACCGGTA | + |
| A_23_P103996 | GCLM | glutamate-cysteine ligase, modifier subunit | ATCAAATCAAAAGGCTACATTTTACAAGCTAAAAGAAGGGGTTCTTAACTGACTTAGGAG | + |
| A_23_P105138 | CAT | catalase | CTCATCACTGGATGAAGATTCTCCTGTGCTAGATGTGCAAATGCAAGCTAGTGGCTTCAA | + |
| A_23_P106174 | PSEN1 | presenilin 1 | TATCCTCCTGGTGGTTCTGTATAAATACAGGTGCTATAAGGTGAGCATGAGACACAGATC | + |
| A_23_P107701 | ERCC1 | excision repair cross-complementing rodent repair deficiency, complementation group 1 (includes overlapping antisense sequence) | CCTATGAGCAGAAACCAGCGGACCTCCTGATGGAGAAGCTAGAGCAGGACTTCGTCTCCC | + |
| A_23_P109143 | PRNP | prion protein | TATATATTGCATAGGACAGACTTAGGAGTTTTGTTTAGAGCAGTTAACATCTGAAGTGTC | + |
| A_23_P110712 | DUSP1 | dual specificity phosphatase 1 | TGTCTACTCCTAGAAGAACCAAATACCTCAATTTTTGTTTTTGAGTACTGTACTATCCTG | + |
| A_23_P114862 | ANGPTL7 | angiopoietin-like 7 | AAGGAGCTTCCTTTTAAATTTTGTCTGTAGGAAATGGTTGAAAACTGAAGGTAGATGGTG | - |
| A_23_P115261 | AGT | angiotensinogen (serpin peptidase inhibitor, clade A, member 8) | TGTTCCAAAAAGAATTCCAACCGACCAGCTTGTTTGTGAAACAAAAAAGTGTTCCCTTTT | + |
| A_23_P11995 | PRDX1 | peroxiredoxin 1 | TCAGGATTATGGGGTCTTAAAGGCTGATGAAGGCATCTCGTTCAGGGGCCTTTTTATCAT | + |
| A_23_P120883 | HMOX1 | heme oxygenase (decycling) 1 | TGGGGAGGGAGGTGTTTAACGGCACTGTGGCCTTGGTCTAACTTTTGTGTGAAATAATAA | + |
| A_23_P121926 | SEPP1 | selenoprotein P, plasma, 1 | CGTAAACTATGACCTAGGGGTTTTCTGTTGGATAATTAGCAGTTTAGAATGGAGGAAGAA | + |
| A_23_P123608 | JAK2 | Janus kinase 2 | GGATAACATGGCTGGATGAAAGAAATGACCTTCATTCTGAGACCAAAGTAGATTTACAGA | + |
| A_23_P126135 | MFN2 | mitofusin 2 | TTGTTTTTATGTTCATTTGCTGGAGCGCAAGACGTGCTGACACAGTGAGTTTTCTCTGAT | + |
| A_23_P128447 | LRRK2 | leucine-rich repeat kinase 2 | GCAGAAAGAGATACAATCTTGCTTGACCGTTTGGGACATCAATCTTCCACATGAAGTGCA | + |
| A_23_P129157 | NEIL1 | nei endonuclease VIII-like 1 (E. coli) | TAGCAGGAGGCTCTCCTTGCTTGCACTCACCCTTTCTTATTGTCTTGCCCTGCATCTGGG | + |
| A_23_P1292 | ERCC6 | excision repair cross-complementing rodent repair deficiency, complementation group 6 | GCTAAACAACATTGCTTCCTAAACTTTCAAGTCCCTTTTTCTAACGGGCATTTCTGATTA | + |
| A_23_P129629 | MT3 | metallothionein 3 | AGTGTGGCTGGTGTCCCCTTCCCCTGCTGACCTTGGAGGAATGACAATAAATCCCATGAG | + |
| A_23_P12989 | PRDX5 | peroxiredoxin 5 | ATCTTTGGGAATCGACGTCTCAAGAGGTTCTCCATGGTGGTACAGGATGGCATAGTGAAG | + |
| A_23_P129896 | ALDH3A2 | aldehyde dehydrogenase 3 family, member A2 | CTTCCTGGAAGTTAGTTGCCAAAGTCATGCAAGCATCACCTGTCATTCTTGTGTTGGAGT | + |
| A_23_P130488 | ERCC2 | excision repair cross-complementing rodent repair deficiency, complementation group 2 | CAGAGGAACAAGCTGCTCTTTATTGAGACCCAGGATGGTGCCGAAACCAGTGTCGCCCTG | + |
| A_23_P131801 | SGK2 | serum/glucocorticoid regulated kinase 2 | GAATTACCTTCAGCTGCTAGGAAGAGCGACTCAAACTAACAATGGCTTCAACGAGAAGCA | + |
| A_23_P132611 | VHL | von Hippel-Lindau tumor suppressor | AGCTCAAGACCAGCCTGGCTAACATGGTGAAACCTCATCTCCACTTAAAATACAAAAATT | + |
| A_23_P134167 | PDSS2 | prenyl (decaprenyl) diphosphate synthase, subunit 2 | AGCTTAAGAGAACACTGTTCTGTTTGAAATGCTTTCTGTCACTGAAATTGGCTTAATTAG | + |
| A_23_P134176 | SOD2 | superoxide dismutase 2, mitochondrial | TTGATGTGTGGGAGCACGCTTACTACCTTCAGTATAAAAATGTCAGGCCTGATTATCTAA | + |
| A_23_P134295 | NUDT1 | nudix (nucleoside diphosphate linked moiety X)-type motif 1 | AATTCCACGGGTACTTCAAGTTCCAGGGTCAGGACACCATCCTGGACTACACACTCCGCG | + |
| A_23_P136077 | PARK2 | parkinson protein 2, E3 ubiquitin protein ligase (parkin) | GTACAACCACTATCTTTTGTTCTACCTGTATTGTCTGACTTCTCAGGAAGATCGTGAACA | + |
| A_23_P137856 | MUC1 | mucin 1, cell surface associated | GTACCGATCGTAGCCCCTATGAGAAGGTTTCTGCAGGTAATGGTGGCAGCAGCCTCTCTT | + |
| A_23_P139265 | SIRT3 | sirtuin 3 | CAGAAGAGATGCGGGACCTTGTGCAGCGGGAAACTGGGAAGCTTGATGGACCAGACAAAT | + |
| A_23_P141173 | MPO | myeloperoxidase | CCTGTTCTGGGTGCAGCTGAGAAAATGAGTGACTAGACGTTCATTTGTGTGCTCATGTAT | + |
| A_23_P142045 | PRDX2 | peroxiredoxin 2 | CCAACGTGGATGACAGCAAGGAATATTTCTCCAAACACAATTAGGCTGGCTAACGGATAG | + |
| A_23_P144054 | PRKCD | protein kinase C, delta | GTCTGCATTCGCTGGCTTCTCCTTTGTGAACCCCAAATTCGAGCACCTCCTGGAAGATTG | + |
| A_23_P144877 | ATOX1 | ATX1 antioxidant protein 1 homolog (yeast) | TGAAGAAAACAGGAAAGACTGTTTCCTACCTTGGCCTTGAGTAGCAGGGGCCTGGTCCCC | + |
| A_23_P145114 | GCLC | glutamate-cysteine ligase, catalytic subunit | AGAATGCCTGGTTTTCGTTTGCAATTTGCTTGTGTAAATCAGGTTGTAAAAAGGCAGATA | + |
| A_23_P145204 | HFE | hemochromatosis | CTATGAGATAGGTACTATTATCCCCATTTCTTTTTTAAATGAAGAAAGTGAAGTAGGCCG | + |
| A_23_P145357 | BAK1 | BCL2-antagonist/killer 1 | AAGGACTATCAACACCACTAGGAATCCCAGAGGTGGGATCCTCCCTCATGGCTCTGGCAC | + |
| A_23_P145844 | MET | met proto-oncogene (hepatocyte growth factor receptor) | AAAGCAACAGTCCACACTTTGTCCAATGGTTTTTTCACTGCCTGACCTTTAAAAGGCCAT | + |
| A_23_P145846 | MET | met proto-oncogene (hepatocyte growth factor receptor) | TGTGAACGTAAAATGTGTCGCTCCGTATCCTTCTCTGTTGTCATCAGAAGATAACGCTGA | + |
| A_23_P147465 | PARK2 | parkinson protein 2, E3 ubiquitin protein ligase (parkin) | GATGTTTTAATTCCAAACCGGATGAGTGGTGAATGCCAATCCCCACACTGCCCTGGGACT | + |
| A_23_P149470 | NDUFS2 | NADH dehydrogenase (ubiquinone) Fe-S protein 2, 49kDa (NADH-coenzyme Q reductase) | TTGGGGTTGTAACAGCAGAAGAAGCACTTAACTATGGTTTTAGTGGAGTGATGCTTCGGG | + |
| A_23_P149992 | PDLIM1 | PDZ and LIM domain 1 | CAAGCAGCCTTGTCATCGACAAAGAATCTGAAGTTTACAAGATGCTTCAGGAGAAACAGG | + |
| A_23_P152322 | COQ7 | coenzyme Q7 homolog, ubiquinone (yeast) | TTCTTCAGCTGATAAAGAAATTTCGGGATGAAGAGCTTGAGCACCATGACATAGGCCTCG | + |
| A_23_P152906 | ALOX12 | arachidonate 12-lipoxygenase | CTGCAAAGACTAGATCCTTTTTTACGCTTTGCAGACCGCATAGTCACTGTCTCAACTACT | + |
| A_23_P152909 | ALOX12 | arachidonate 12-lipoxygenase | ATCTTGAATTTCATGCTTTCCTAAAGTCTCTGCTGCTAAGGCTCTATTTCCTCCCCCAGT | + |
| A_23_P154840 | SOD1 | superoxide dismutase 1, soluble | AAACATTCCCTTGGATGTAGTCTGAGGCCCCTTAACTCATCTGTTATCCTGCTAGCTGTA | + |
| A_23_P160449 | PPP1R15B | protein phosphatase 1, regulatory (inhibitor) subunit 15B | CTAGCATACACTACCTCTTACCTGAGAGGTGTCTTTTAAAAACAAATCTTGGCAGCTGTC | + |
| A_23_P161727 | HSPB2 | heat shock 27kDa protein 2 | TATGGTTTGGTCCCATGGGACATGTCATAGCCTTGGTTTAGTTTTGGGTGGAGCTGAATA | + |
| A_23_P163782 | MT1H | metallothionein 1H | AAGTGCAAATGCACCTCCTGCAAGAAGAGCTGCTGCTCCTGTTGCCCCCTGGGCTGTGCC | + |
| A_23_P163787 | MMP2 | matrix metallopeptidase 2 (gelatinase A, 72kDa gelatinase, 72kDa type IV collagenase) | TTCTTCAAGGGTGCCTATTACCTGAAGCTGGAGAACCAAAGTCTGAAGAGCGTGAAGTTT | + |
| A_23_P163955 | PEMT | phosphatidylethanolamine N-methyltransferase | CTGGGGTTCGCTGGAACTTTCCTAGGTGATTACTTCGGGATCCTCAAGGAGGCGAGAGTG | + |
| A_23_P164650 | APOE | apolipoprotein E | GCCTTCCAGGCCCGCCTCAAGAGCTGGTTCGAGCCCCTGGTGGAAGACATGCAGCGCCAG | + |
| A_23_P164883 | PNKP | polynucleotide kinase 3'-phosphatase | GGGGCGGAAGAAGAAAGACTTCTCCTGCGCCGATCGCCTGTTTGCCCTCAACCTTGGCCT | + |
| A_23_P166306 | CBS | cystathionine-beta-synthase | GCTGATCGACTTTGTGTCTCTGTTGTCTAAAATAGGTTTTCCCTGTTCTGGACATTTCAT | + |
| A_23_P18505 | GAB1 | GRB2-associated binding protein 1 | ATAGTATTGTTTAGCTCCCAGAGAAACATTTGTTCCACAGTTAACACACTCGTAGTATTA | + |
| A_23_P20225 | RRM2B | ribonucleotide reductase M2 B (TP53 inducible) | TGCTCCTTTGTAAAAAGTTAAAGATTTGAAAGAGAATCTCATATTCCCGAGGCATTAGGA | + |
| A_23_P20248 | MAP2K1 | mitogen-activated protein kinase kinase 1 | ACAGTGAAATTTTGGTGAATGTGGGTAGTCATTCTTACAATTGCACTGCTGTTCCTGCTC | + |
| A_23_P20316 | CA3 | carbonic anhydrase III, muscle specific | AGACAGCAAGAATTGAGCTAATAATATGTTTTAACTCTTAACACCAGCAAGAAGTCAGTC | + |
| A_23_P203601 | UCP3 | uncoupling protein 3 (mitochondrial, proton carrier) | ACCTAAAGTAGCAGGACTCGGAATTCTCGGGAAATTATTATGACTCAATAAAAGAATTCA | + |
| A_23_P204581 | TXNRD1 | thioredoxin reductase 1 | CAAAAGCAAGTCATGGCTAGAGTATCCATGCAAGGTGTCTTGTTGCATGGAAGGGATAGT | + |
| A_23_P205611 | GMFB | glia maturation factor, beta | GAAAATGAGTGAGTCTTTGGTTGTTTGTGATGTTGCCGAAGATTTAGTGGAAAAGCTGAG | + |
| A_23_P205686 | PSEN1 | presenilin 1 | CTTTGGCAATTCTTCTTCTCAAGCACTGACACTCATTACCGTCTGTGATTGCCATTTCTT | + |
| A_23_P206661 | NQO1 | NAD(P)H dehydrogenase, quinone 1 | GTTTATATACAGTACACAGATACCTTGAAAGGAAGAGCTAATAAATCTCTTCTTTGCTGC | + |
| A_23_P206724 | MT1E | metallothionein 1E | GGCATCGGAGAAGTGCAGCTGCTGTGCCTGATGTGGGAACAGCTCTTCTCCCAGATGTAA | + |
| A_23_P206776 | TAT | tyrosine aminotransferase | CAGTGTGTCCCCATCTTAGCTGATGAGATCTATGGAGACATGGTGTTTTCGGATTGCAAA | + |
| A_23_P207400 | BRCA1 | breast cancer 1, early onset | AAGTGTTTTTCATAAACCCATTATCCAGGACTGTTTATAGCTGTTGGAAGGACTAGGTCT | + |
| A_23_P207699 | MAPT | microtubule-associated protein tau | ACCAGTTCTCTTTGTAAGGACTTGTGCCTCTTGGGAGACGTCCACCCGTTTCCAAGCCTG | + |
| A_23_P208132 | BCL2 | B-cell CLL/lymphoma 2 | ATCAGAGTTGTTGCTTCCCGGCGTCCCTACCTCCTCCTCTGGACAAAGCGTTCACTCCCA | + |
| A_23_P208595 | LDLR | low density lipoprotein receptor | GTACCTTCCTTAAGCCAGGAAAGGGATTCATGGCGTCGGAAATGATCTGGCTGAATCCGT | + |
| A_23_P208706 | BAX | BCL2-associated X protein | TGCCTTGGACTGTGTTTTTCCTCCATAAATTATGGCATTTTTCTGGGAGGGGTGGGGATT | + |
| A_23_P209430 | ALS2 | amyotrophic lateral sclerosis 2 (juvenile) | GGGAGAAAGGAACTTTTAAACTGCAGTTTTAACTTTTTCTAAGCTGTTTCTTGATGGGAG | + |
| A_23_P210210 | EPAS1 | endothelial PAS domain protein 1 | ACACTGAAAAATATTCCAAGCTTCATATTAACCCTACCTGTCAACGTAACGATTTCATGA | + |
| A_23_P210395 | CHRNA4 | cholinergic receptor, nicotinic, alpha 4 | AGCTCAGCACAGCCTCACCCCTGCAGGCGGTATCCAGAGGTGAGGGAGGCCTGAAATGTT | + |
| A_23_P210920 | GSS | glutathione synthetase | ATCCTTGAGATGTGGGTATAGCTCAGGGTAAGCTGCTCTGAGGTAAAGGTCCATGAACCC | + |
| A_23_P212196 | OGG1 | 8-oxoguanine DNA glycosylase | GTTCATCCCTTTTTCTGCTAATTCGAGTCATGGCTAATTTAACACCCTTTAGAACCTTAA | + |
| A_23_P212749 | HTT | huntingtin | CTTCCCCTCAGTTGTTTCTAAGAGCAGAGTCTCCCGCTGCAATCTGGGTGGTAACTGCCA | + |
| A_23_P215406 | RAC1 | ras-related C3 botulinum toxin substrate 1 (rho family, small GTP binding protein Rac1) | TGTTAGTCGCTAACTTAGTAAGTGCTTTTCTTATAGAACCCCTTCTGACTGAGCAATATG | + |
| A_23_P215566 | AHR | aryl hydrocarbon receptor | AATGGCTTCGGACAAAATATCTCTGAGTTCTGTGTATTTTCAGTCAAAACTTTAAACCTG | + |
| A_23_P215900 | SCARA3 | scavenger receptor class A, member 3 | TTCCCTGCTCAAAGCCTTTCCGCACCCTCCTGACTGTCCTGGATGTGCAACTGGTTTGCA | + |
| A_23_P215956 | MYC | v-myc myelocytomatosis viral oncogene homolog (avian) | TTCAAATGCATGATCAAATGCAACCTCACAACCTTGGCTGAGTCTTGAGACTGAAAGATT | + |
| A_23_P217258 | CYBB | cytochrome b-245, beta polypeptide | ACAATTGCAAGTCAACACCCTAATACCAGAATAGGAGTTTTCCTCTGTGGACCTGAAGCC | + |
| A_23_P219176 | RXRA | retinoid X receptor, alpha | AAGAGATGTGTTGTCACCCTCCTTATTTCTGTTACTACTTGTCTGTGGCCCAGGGCAGTG | + |
| A_23_P250813 | WRN | Werner syndrome, RecQ helicase-like | GCAATTCATGTAGTTTCTGGGTCTTCTGGGAGCCTACGTGAGTACATCACCTAACAGAAT | + |
| A_23_P252653 | STK25 | serine/threonine kinase 25 | CATAAGAACTGTGCTGACTTGGAAGGTGCCCTGTGCTATGTCGTGCCTGCAGGGACACGT | + |
| A_23_P254081 | LIAS | lipoic acid synthetase | TTTTTAAAATTTGATTCCAGTTAATAACAGAGGTGGTGCCAGAATGCCTGGACTGCAGTG | + |
| A_23_P255345 | VNN1 | vanin 1 | CTTGTTTAGTCTGAAGCCAACATCCGGACCTGTCTTAACAGTAACTCTGTTTGGGAGGTT | + |
| A_23_P256244 | OXR1 | oxidation resistance 1 | CAGTGTCTACTGTCTTTTCTTATCCCACAGGTGGATTGTACACATAATGAGCATCATTGC | + |
| A_23_P256384 | PSIP1 | PC4 and SFRS1 interacting protein 1 | TTGTGTTTGAAAGTAATAACAAGCTCAGACGAAGATGGTGGTTGTACATTATTCATCTAG | + |
| A_23_P258312 | NAPRT1 | nicotinate phosphoribosyltransferase domain containing 1 | CCCTGGTGAACAGTCTGTGTGCGGGGCAGTCCCCCTGAGACTCGGAGCGGGGCTGACTGG | + |
| A_23_P29939 | SNCA | synuclein, alpha (non A4 component of amyloid precursor) | TGACAGATGTTCCATCCTGTACAAGTGCTCAGTTCCAATGTGCCCAGTCATGACATTTCT | + |
| A_23_P30024 | NFKB1 | nuclear factor of kappa light polypeptide gene enhancer in B-cells 1 | CCCGCCTGAATCATTCTCGATTTAACTCGAGACCTTTTCAACTTGGCTTCCTTTCTTGGT | + |
| A_23_P305060 | NAMPT | nicotinamide phosphoribosyltransferase | TGCCTGTGGCTCTAATATGCACCTCAAGATTTTAAGGAGATAATGTTTTTAGAGAGAATT | + |
| A_23_P31399 | PON2 | paraoxonase 2 | CATTGACCCAGAAATGTATGGCATGTGTAGTTAATTTTATTCCAGTAAGGAACGGCCCTT | + |
| A_23_P315273 | MT3 | metallothionein 3 | AAGGACTGTGTGTGCAAAGGCGGAGAGGCAGCTGAGGCAGAAGCAGAGAAGTGCAGCTGC | + |
| A_23_P320113 | SRXN1 | sulfiredoxin 1 | GTTCAATAATAAGGACAAGAGCTTTTCCCATGCATTCTCTTTCCCCGGGAAAGTTGACTG | + |
| A_23_P325726 | ACOT11 | acyl-CoA thioesterase 11 | GGAGATCAGTCAGGTCCGCCTGTACACTCTGGAGGATGACAAGTTCCTCTCCTTCCACAT | + |
| A_23_P335239 | GAB1 | GRB2-associated binding protein 1 | GTTGAAGTCAAAGGACCTTTCTGACATAATCAAGCAATTTAGACTTAAGTGGTGCTTTGT | + |
| A_23_P338981 | CYGB | cytoglobin | CTCAAGCACAAGGTGGAACCGGTGTACTTCAAGATCCTCTCTGGGGTCATTCTGGAGGTG | + |
| A_23_P340158 | ISCU | iron-sulfur cluster scaffold homolog (E. coli) | GGGGGAAATTACCAGTAGAATGCCTTGGTCTGAATATTTGATAGAACCAATTGTTGTACA | + |
| A_23_P340728 | PSEN1 | presenilin 1 | TTCTACTTTGCCACAGATTATCTTGTACAGCCTTTTATGGACCAATTAGCATTCCATCAA | + |
| A_23_P34093 | G6PD | glucose-6-phosphate dehydrogenase | CCACGGAGGCAGACGAGCTGATGAAGAGAGTGGGTTTCCAGTATGAGGGCACCTACAAGT | + |
| A_23_P342009 | HFE | hemochromatosis | GAGTCATCAGTGGAATTGCTGTTTTTGTCGTCATCTTGTTCATTGGAATTTTGTTCATAA | + |
| A_23_P344392 | OGG1 | 8-oxoguanine DNA glycosylase | GCTGTGCCAGGCTTTTGGACCTCGGCTCATCCAGCTTGATGATGTCACCTACCATGGCTT | + |
| A_23_P346309 | BAX | BCL2-associated X protein | CCCGCGCGGACCCGGCGAGAGGCGGCGGCGGGAGCGGCGGTGATGGACGGGTCCGGGGAG | + |
| A_23_P346311 | BAX | BCL2-associated X protein | GAGCAGGGCGAATGGGGGGGGAGGCACCCGAGCTGGCCCTGGACCCGGTGCCTCAGGATG | + |
| A_23_P34637 | REN | renin | TACAGTAGTAAAAAGCTGTGCACACTGGCCATCCACGCCATGGATATCCCGCCACCCACT | + |
| A_23_P34767 | YBX1 | Y box binding protein 1 | AAATGAACAAAAGATTGGAGCTGAAGACCTAAAGTGCTTGCTTTTTGCCCGTTGACCAGA | + |
| A_23_P352266 | BCL2 | B-cell CLL/lymphoma 2 | TGGCTGATATTCTGCAACACTGTACACATAAAAAATACGGTAAGGATACTTTACATGGTT | + |
| A_23_P352879 | GCLC | glutamate-cysteine ligase, catalytic subunit | CTCTCAGCCCGTGTGTATAATATGAAGACCAAATGATAGAACTGTACTGTTTTCTGGGCC | + |
| A_23_P359245 | MET | met proto-oncogene (hepatocyte growth factor receptor) | GTGTCTGGACAGATTGTGGGAGTAAGTGATTCTTCTAAGAATTAGATACTTGTCACTGCC | - |
| A_23_P364517 | SELK | selenoprotein K | TCTGCAGGGAACTGGCCTGACTGACATGCAGTTCCATAAATGCAGATGTTTGTCTCATTA | + |
| A_23_P36757 | HNF1A | HNF1 homeobox A | CGTCCCCAGCCAGGACCCTGCCAGCATCCAGCACCTGCAGCCGGCCCACCGGCTCAGCGC | + |
| A_23_P371777 | ACE | angiotensin I converting enzyme (peptidyl-dipeptidase A) 1 | AGAAAAAGGACTCTGCTTCAGTCCACAGTACATAAAATGTACTCAGCTATCAGCTCTATA | + |
| A_23_P37191 | PSMB5 | proteasome (prosome, macropain) subunit, beta type, 5 | GGCCACCTTCTCTGTAGGTTCTGGCTCTGTGTATGCATATGGGGTCATGGATCGGGGCTA | + |
| A_23_P376488 | TNF | tumor necrosis factor | GGGGTATCCTGGGGGACCCAATGTAGGAGCTGCCTTGGCTCAGACATGTTTTCCGTGAAA | + |
| A_23_P377664 | ALS2 | amyotrophic lateral sclerosis 2 (juvenile) | AGATCTGTCCTGCAGTGTTTCTTTTTAGGTTTTTGAAACCACATAGCCTCTTGATTCTCA | + |
| A_23_P38235 | ACE | angiotensin I converting enzyme (peptidyl-dipeptidase A) 1 | AAGCCATGCAGCTGATCACGGGCCAGCCCAACATGAGCGCCTCGGCCATGTTGAGCTACT | + |
| A_23_P386411 | PPID | peptidylprolyl isomerase D | GACACCTTAGTTCCTTACTGTTTACAGTTTAGGAGTACTGATAGGGGTTCATGCTTAATA | + |
| A_23_P387471 | MICB | MHC class I polypeptide-related sequence B | TATGCATTACTCTGTGTCTACTATTATGTGTGCATAATTTATACCGTAAATGTTTACTCT | + |
| A_23_P38959 | VAV1 | vav 1 guanine nucleotide exchange factor | CTATGACTTCTGCGCCCGAGACCGATCAGAGCTGTCGCTCAAGGAGGGTGACATCATCAA | + |
| A_23_P389897 | NGFR | nerve growth factor receptor | TGTGGAAGGGACTAGGAGCACTGTAGTAAATGGCAATTCTTTGACCTCAACCTGTGATGA | + |
| A_23_P39590 | XDH | xanthine dehydrogenase | CACAGATATTGTCATGGATGTTGGCTCCAGTCTAAACCCTGCCATTGATATTGGACAGGT | + |
| A_23_P40174 | MMP9 | matrix metallopeptidase 9 (gelatinase B, 92kDa gelatinase, 92kDa type IV collagenase) | TGGAGGTGGGCTGGGCCCTCTCTTCTCACCTTTGTTTTTTGTTGGAGTGTTTCTAATAAA | + |
| A_23_P414343 | MT1H | metallothionein 1H | TGTGCCAAGTGTGCCCACGGCTGCATCTGCAAAGGGACGTCGGAGAAGTGCAGCTGCTGT | + |
| A_23_P417415 | ACOT11 | acyl-CoA thioesterase 11 | GTCCCTTGTTAAAGGGGCAGTGGGAGTTATGGGGTCATCAAGGACCTTGCCTCTCTGGAA | + |
| A_23_P418413 | OXSR1 | oxidative-stress responsive 1 | TGGTCTTTCTAAACGACTAAAGGATTTGTTGGGTTTTTGCTTAAGTTTTGAACCAAATCC | + |
| A_23_P420942 | MT1E | NaN | TTTTTCTCGTGGGACACAAACCCCAACTGTACCCCCTATGGTTTCAGAACAGAGCTGTGC | + |
| A_23_P423197 | RXRA | retinoid X receptor, alpha | AGGCAGGAGATGCATCTATTTTAAGATGCTTTGGAGCAGACAGCTTTAGCCGTTCCCAAT | + |
| A_23_P426292 | MAPK14 | mitogen-activated protein kinase 14 | GAATGAGGGAAATTGCTATTTTATTTGTATTCATGAACTTGGCTGTAATCAGTTATGCCG | + |
| A_23_P430120 | EPAS1 | endothelial PAS domain protein 1 | TGCCGGACAAGCCACTGAGCGCAAATGTACCCAATGATAAGTTCACCCAAAACCCCATGA | + |
| A_23_P43238 | NAPRT1 | nicotinate phosphoribosyltransferase domain containing 1 | AGGCGAGCGGGCAGCCTTTGTGGCCTATGCCTTGGCTTTTCCCCGGGCCTTCCAGGGCCT | + |
| A_23_P45022 | PPID | peptidylprolyl isomerase D | CTAATTTTTGTATTTTTAGTAGAGATGGGGTTTCACCATATTGGTCACGTCACGTTGGTC | + |
| A_23_P45396 | HSD17B10 | hydroxysteroid (17-beta) dehydrogenase 10 | ATGCTCACCTCGTACAGGCCATCATCGAGAACCCATTCCTCAATGGAGAGGTCATCCGGC | + |
| A_23_P46829 | FGF8 | fibroblast growth factor 8 (androgen-induced) | GGCCTCTACATCTGCATGAACAAGAAGGGGAAGCTGATCGCCAAGAGCAACGGCAAAGGC | + |
| A_23_P46903 | CAMK2G | calcium/calmodulin-dependent protein kinase II gamma | GGATTTCCATAAGTTTTACTTTGAGAATCTCCTGTCCAAGAACAGCAAGCCTATCCATAC | + |
| A_23_P4764 | INSR | insulin receptor | GTTCAGAGATCGTTCCTATACATTTCTGTTCATCTTAAGGTGGACTCGTTTGGTTACCAA | + |
| A_23_P47800 | DIABLO | diablo, IAP-binding mitochondrial protein | TTTTTGTCATGCCCTTAAGTTCAGCAACTGTTTAACCTGTTTTCAGTCTTATTTACGTCG | + |
| A_23_P502464 | NOS2 | nitric oxide synthase 2, inducible | TGTGTACAGTTATTTATGCCTCTGTATTTAAAAAACTAACACCCAGTCTGTTCCCCATGG | + |
| A_23_P51660 | MUTYH | mutY homolog (E. coli) | ATGGGCCAGCAAGTCCTGGATAATTTCTTTCGGTCTCACATCTCCACTGATGCACACAGC | + |
| A_23_P5325 | ERCC3 | excision repair cross-complementing rodent repair deficiency, complementation group 3 (xeroderma pigmentosum group B complementing) | GACACACAGGAAATGGCTTACTCAACCAAGCGGCAGAGATTCTTGGTAGATCAAGGTTAT | + |
| A_23_P5761 | NFE2L2 | nuclear factor (erythroid-derived 2)-like 2 | CAGTAGTTTCACTTTAACTGTAAACAATTTCTTAGGACACCATTTGGGCTAGTTTCTGTG | + |
| A_23_P58521 | ERCC8 | excision repair cross-complementing rodent repair deficiency, complementation group 8 | ACTATGCTTAAGGGACATTATAAAACTGTTGACTGCTGTGTATTTCAGTCAAATTTCCAG | + |
| A_23_P59418 | NRF1 | nuclear respiratory factor 1 | GGAATTGCATTTTTTAAAGCACCACTCTTGATTTTCTGGGATTGGTGAAGAAACTGCATT | + |
| A_23_P60248 | TXN | thioredoxin | GGACAAAAGGTGGGTGAATTTTCTGGAGCCAATAAGGAAAAGCTTGAAGCCACCATTAAT | + |
| A_23_P60283 | XPA | xeroderma pigmentosum, complementation group A | TGTTATGGCTTAAAAGCAAGTTTCAGTGAAGGTCACCTGGCCTGGTTGTGTGCACAATGT | + |
| A_23_P60306 | TLR4 | toll-like receptor 4 | TTTTTTCAGAACAAGTGATGTTTGATGGACCTCTGAATCTCTTTAGGGAGACACAGATGG | + |
| A_23_P61426 | MSRA | methionine sulfoxide reductase A | TCTCTGTGCAGAGAAAAGATGTGAGTCCGCTTGATGAATTCTAATGCTTTGCTTAGAGCT | + |
| A_23_P61447 | ETFDH | electron-transferring-flavoprotein dehydrogenase | AAGGTGGAGGAGGACCTGCTTACAATGGAATGTAAACTGCAGCTAGCCAGTTTCTTTCAA | + |
| A_23_P6335 | SERPIND1 | serpin peptidase inhibitor, clade D (heparin cofactor), member 1 | CCCTCATCTGAATACCAAGCACAGAAATGAGTGGTGTGACTAATTCCTTACCTCTCCCAA | + |
| A_23_P63751 | PRDX3 | peroxiredoxin 3 | AACATCGCACTCTTGTCAGACTTAACTAAGCAGATTTCCCGAGACTACGGTGTGCTGTTA | + |
| A_23_P69468 | NDUFB4 | NADH dehydrogenase (ubiquinone) 1 beta subcomplex, 4, 15kDa | TGGATCGAACATTTCACCTCTCATATTAAGTCTGGCAATGATGACTATATGTATTCCTGC | + |
| A_23_P70849 | NOS3 | nitric oxide synthase 3 (endothelial cell) | TCACCGCCTTCTCCCGGGAACCTGACAACCCCAAGACCTACGTGCAGGACATCCTGAGGA | + |
| A_23_P72537 | AIFM1 | apoptosis-inducing factor, mitochondrion-associated, 1 | TATTCAGCAGACTTTCTCTGTGTATGAGTGTGAATGATCAAGTCCTTTGTGAATATTTTC | + |
| A_23_P73972 | GPX7 | glutathione peroxidase 7 | ATTAGGATGAAATACCTGTGAAAGTGCCTAGGCAGTGCCAGCCAAATAGGAGGCATTCAA | + |
| A_23_P74001 | S100A12 | S100 calcium binding protein A12 | TGAAGGCTTTTTACCCAGCAATGTCCTCAATGAGGGTCTTTTCTTTCCCTCACCAAAACC | + |
| A_23_P74740 | PARK7 | parkinson protein 7 | CACTGTGTTCGCTCTAAACAAAACAGTGGTAGGTTAATGTGTTCAGAAGTCGCTGTCCTT | + |
| A_23_P76557 | NDUFA12 | NADH dehydrogenase (ubiquinone) 1 alpha subcomplex, 12 | TACTACTGAAATGAATGGCAAAAACACATTCTGGGATGTGGATGGAAGCATGGTGCCTCC | + |
| A_23_P78302 | NFE2L1 | nuclear factor (erythroid-derived 2)-like 1 | CTGGACCTGGACCTACAGCGGGGACTTAAATGCCTTCTTATCCAATATATCTTCTCAGAT | + |
| A_23_P79562 | FABP1 | fatty acid binding protein 1, liver | TGTGACCGAACTCAACGGCGACATAATCACCAATACCATGACATTGGGTGACATTGTCTT | - |
| A_23_P79931 | ATRN | attractin | GTGGCTACTCCTGACGAACAAAACAGGGATTTGGACATGTTCATCAATGCCTCCAAGAAT | + |
| A_23_P83098 | ALDH1A1 | aldehyde dehydrogenase 1 family, member A1 | GGAGAGTACGGTTTCCATGAATATACAGAGGTCAAAACAGTCACAGTGAAAATCTCTCAG | + |
| A_23_P85008 | MAOB | monoamine oxidase B | TATCTCTTCTTCCTTTTGTATCCTCCATTGTATCTTCATACAAAGGACAGTACACACTTG | + |
| A_23_P85015 | MAOB | monoamine oxidase B | ACCTTCCCTAGTTCTTTGCATTCGTCCTTAGAATACTGTATTGTTACAGCTGAAAGACAG | + |
| A_23_P86774 | NDUFS8 | NADH dehydrogenase (ubiquinone) Fe-S protein 8, 23kDa (NADH-coenzyme Q reductase) | TGACATCGACATGACCAAGTGCATCTACTGCGGCTTCTGCCAGGAGGCCTGTCCCGTGGA | + |
| A_23_P91769 | NDUFA6 | NADH dehydrogenase (ubiquinone) 1 alpha subcomplex, 6, 14kDa | ACGATCCATGAAGTCATTCAGTGGAAAGATGCACGTTGATACTATTTTAGAGCACAAATA | + |
| A_23_P94204 | OXR1 | oxidation resistance 1 | CATTGAGGAAATGAAGACTGGATACTTCTGTATCTGTGAAGTTGGCACAGGTAACATTTG | + |
| A_23_P97700 | TXNIP | thioredoxin interacting protein | TCCACCCTTTTCTGAGAGTTATTACAGCCAGAAAGTGTGGGCTGAAGATGGTTGGTTTCA | + |
| A_23_P98002 | CYP2E1 | cytochrome P450, family 2, subfamily E, polypeptide 1 | TGCCATTTTGCAGCATTTTAATTTGAAGCCTCTCGTTGACCCAAAGGATATCGACCTCAG | + |
| A_23_P98022 | SIRT1 | sirtuin 1 | CTGCCTTAAAACTAGAGATCAACTTTCTCAGCTGCAAAAGCTTCTAGTCTTTCAAGAAGT | + |
| A_23_P983 | PRDX6 | peroxiredoxin 6 | CCTGGTGTCATCACAGCCAAGGTTTTTAGGTTGCTATACCAATGGCTTATTAAATGAAAA | + |
| A_24_P101391 | YBX1 | Y box binding protein 1 | CATTATAGACGCTATCCACGTCGTGGTCCTCCACGCAATTACCAGCAAAATTACCAGAAT | + |
| A_24_P103448 | HFE | hemochromatosis | CTAAAGACGTATTGCCCAATGGGGATGGGACCTACCAGGGCTGGATAACCTTGGCTGTAC | + |
| A_24_P111996 | HFE | hemochromatosis | GAGACAAAACTAGAGACTCAAAGAGGGAGTGCATTTATGAGCTCTTCATGTTTCAGGAGA | + |
| A_24_P117029 | LDLR | low density lipoprotein receptor | ATTGCCTCTGAAATGCCTCTTCTTTATGTACAAAGATTATTTGCACGAACTGGACTGTGT | + |
| A_24_P134942 | VHL | von Hippel-Lindau tumor suppressor | TCTAAACTAGGATTGACATTCTACAGTTGTGATAATAGCATTTTTGTAACTTGCCATCCG | + |
| A_24_P141332 | CAMK2G | calcium/calmodulin-dependent protein kinase II gamma | ATCACCTTTTAAACAAGAAACGGAAGGCATTTGATGCAGAATTTTTGCATGACAACATAG | + |
| A_24_P151356 | SGK2 | serum/glucocorticoid regulated kinase 2 | AGAGGATGATGACATCTTGGATTGCTAGAAGAGAAGGACCTGTGAAACTACTGAGGCCAG | - |
| A_24_P151464 | SOD1 | superoxide dismutase 1, soluble | TCAGGAGACCATTGCATCATTGGCCGCACACTGGTGGTCCATGAAAAAGCAGATGACTTG | + |
| A_24_P155378 | PRDX5 | peroxiredoxin 5 | TTATTACTAGATGATTCGCTGGTGTCCATCTTTGGGAATCGACGTCTCAAGAGGTTCTCC | + |
| A_24_P163632 | OXR1 | oxidation resistance 1 | GGATTCTTTTCTTCATGAGAATTCGTTACACCAAGAAGAGAGTCAAAAAGAAAATATGCC | + |
| A_24_P168416 | PRDX2 | peroxiredoxin 2 | TGACTTCAAGGCCACAGCGGTGGTTGATGGCGCCTTCAAAGAGGTGAAGCTGTCGGACTA | + |
| A_24_P169688 | MICB | MHC class I polypeptide-related sequence B | CTGGTGCTTCAGAGTCAACGGACAGACTTTCCATATGTTTCTGCTGCTATGCCATGTTTT | + |
| A_24_P172768 | GMFB | glia maturation factor, beta | CTTATTTTGTATTTCTGGAAGAATGTAGTAATCTTCTAGACCGCTTAAAACCAATGCTCC | + |
| A_24_P175519 | TXN | thioredoxin | AAGTAGATGTGGATGACTGTCAGGATGTTGCTTCAGAGTGTGAAGTCAAATGCACGCCAA | + |
| A_24_P178011 | MYC | v-myc myelocytomatosis viral oncogene homolog (avian) | CGCGCGCCCATTAATACCCTTCTTTCCTCCACTCTCCCTGGGACTCTTGATCAAAGCGCG | + |
| A_24_P180225 | HTT | huntingtin | TGAACCTTTTCTGCCTGGTCGCCACAGACTTCTACAGACACCAGATAGAGGAGGAGCTCG | + |
| A_24_P194845 | RAC1 | ras-related C3 botulinum toxin substrate 1 (rho family, small GTP binding protein Rac1) | CCAACGCATTTCCTGGAGAAGATATCCCTACTGCCTTTGACAATTATTCTGCCAATGTTA | + |
| A_24_P216294 | CST3 | cystatin C | GACTTTGCCGTCGGCGAGTACAACAAAGCCAGCAACGACATGTACCACAGCCGCGCGCTG | + |
| A_24_P219552 | NFE2L1 | nuclear factor (erythroid-derived 2)-like 1 | GTTCTCTTTGCCATAAAGACTCCGTGTAACTGTGTGAACACTTGGGATTTTTCTCCTCTG | + |
| A_24_P221445 | OXR1 | oxidation resistance 1 | TGCGTTAGCAGCTGAGCCATTTAAAGTGAGTGATGGCTTTTATGGTACTGGAGAGACCTT | + |
| A_24_P222165 | GAB1 | GRB2-associated binding protein 1 | CCATTTGTTGCCTATTCCACTTAACTTTGTATTTGTTTGAAATCTACTGTTCGGATGCTG | + |
| A_24_P222410 | ALS2 | amyotrophic lateral sclerosis 2 (juvenile) | GCTACTACAGAAAGACGATTCTATTCAAAACTAAGTGATATCAAATCTCAGATTCTCAGG | + |
| A_24_P224488 | MAPT | microtubule-associated protein tau | AGTTTGCCATGTTGAGCAGGACTATTTCTGGCACTTGCAAGTCCCATGATTTCTTCGGTA | + |
| A_24_P227831 | ABCC1 | ATP-binding cassette, sub-family C (CFTR/MRP), member 1 | GGGTAAATATTAAGGAGATGGCCTCATGGGAATTTGACCTTGACTAGAAATAGAGACTGA | + |
| A_24_P232158 | SCARA3 | scavenger receptor class A, member 3 | GAAGACATCTCCTTGACCCAGTCTATTTATGACAAGAAGCTTGTGTTAATGCAGAAAAAT | + |
| A_24_P23245 | NDUFA6 | NADH dehydrogenase (ubiquinone) 1 alpha subcomplex, 6, 14kDa | TTCTGGTCATTAAGGGAAAGATCGAACTGGAAGAAACAATTAAAGTATGGAAGCAGCGGA | + |
| A_24_P26114 | OXSR1 | oxidative-stress responsive 1 | TTTCCAACAACTGATCCTGTGGGTACTTTGCTCCAAGTTCCAGAACAGATCTCTGCTCAT | + |
| A_24_P269687 | TOR1A | torsin family 1, member A (torsin A) | AGCTCAAAGACATTGAACACGCGTTGTCTGTGTCGGTTTTCAATAACAAGAACAGTGGCT | + |
| A_24_P273875 | PRDX3 | peroxiredoxin 3 | ACTCAGCTTCTCTCTCTTGTTAATCTCAATATTAAAACGGGTACAGAGGTAACTGCAAAA | + |
| A_24_P276983 | STK25 | serine/threonine kinase 25 | CTGGTGGAGCGAGTGCAGAGGTTTTCACACAACAGAAACCACCTGACATCCACCCGCTGA | + |
| A_24_P283288 | MAPK14 | mitogen-activated protein kinase 14 | AATGAAGACTGTGAGCTGAAGATTCTGGATTTTGGACTGGCTCGGCACACAGATGATGAA | + |
| A_24_P289648 | HFE | hemochromatosis | AGTTTTACTGGGCATCTCCTGAGCCTAGGCAATAGCTGTAGGGTGACTTCTGGAGCCATC | + |
| A_24_P292470 | UCP3 | uncoupling protein 3 (mitochondrial, proton carrier) | AACTCACCTCCAGGCCAGTACTTCAGCCCCCTCGACTGTATGATAAAGATGGTGGCCCAG | + |
| A_24_P314159 | APP | amyloid beta (A4) precursor protein | AGTGAAGATGGATGCAGAATTCCGACATGACTCAGGATATGAAGTTCATCATCAAAAATT | + |
| A_24_P324986 | ERCC8 | excision repair cross-complementing rodent repair deficiency, complementation group 8 | AAAGTACAACTTTGTGACTTGAAGTCTGGATCCTGTTCTCACATTCTACAGGGTATTTTT | + |
| A_24_P326635 | ADRBK1 | adrenergic, beta, receptor kinase 1 | CCTGCTCCTCAAGATCCGCGGTGGGAAACAGTTCATTTTGCAGTGCGATAGCGACCCTGA | + |
| A_24_P330633 | TAT | tyrosine aminotransferase | TGGGGGGAGTAAAGAATTATTTTGGATGCAAATAAATATCCTTTAATTGATCGACTTGCC | + |
| A_24_P337700 | VNN1 | vanin 1 | TGACATTACACAACTCAGTGAAACAACATCATTTAAGCCAAAATATCTCCCAACTGACTG | + |
| A_24_P354748 | HFE | hemochromatosis | GGTCATCCTGGGCTGTGAAATGCAAGAAGACAACAGTACCGAGGGCTACTGGAAGTACGG | + |
| A_24_P365129 | ACE | angiotensin I converting enzyme (peptidyl-dipeptidase A) 1 | CATTTTAAGGGACATTTTTATGACTTTTATGTGTATGTTTATGTAGAAATTTGGAAAATA | - |
| A_24_P365767 | CYBB | cytochrome b-245, beta polypeptide | GGTTTTTCTTAGTTCTTCTGCTTTTGCAATTGTGTTTGTGAAATTTGAATACTTGCAGGC | + |
| A_24_P372913 | HNF1A | HNF1 homeobox A | GAACCTGGCCTTCAGTGTACCGCGTCTACCCTGGGATTCAGGAAAAGGCCTGGGGTGACC | + |
| A_24_P375002 | YBX1 | Y box binding protein 1 | TGTATTTGTACACCAGACTGCCATAAAGAATAACCCCAGGAAGTACCTTCGCAGTGTAGG | + |
| A_24_P381945 | HMOX2 | heme oxygenase (decycling) 2 | AGGCTGCTTCCGGTAGTCCCTGTTTTTGCAGTACATGGGTGACTATCTCCCCTGTTGGAG | + |
| A_24_P38363 | MYC | v-myc myelocytomatosis viral oncogene homolog (avian) | CGGTTTTCGGGGCTTTATCTAACTCGCTGTAGTAATTCCAGCGAGAGGCAGAGGGAGCGA | + |
| A_24_P394940 | CYP2E1 | cytochrome P450, family 2, subfamily E, polypeptide 1 | TGAAGTAAAAGAGTATGTGTCTGAAAGGGTGAAGGAGCACCATCAATCTCTGGACCCCAA | + |
| A_24_P397566 | MAPK14 | mitogen-activated protein kinase 14 | GGCAGATCTGAACAACATTGTGAAATGTCAGAAGCTTACAGATGACCATGTTCAGTTCCT | + |
| A_24_P397817 | LEP | leptin | GCTGAAGGGACCTTGAAGGGTAAAGAAGTTTGATATTAAAGGAGTTAAGAGTAGCAAGTT | + |
| A_24_P398972 | COQ7 | coenzyme Q7 homolog, ubiquinone (yeast) | CTGGCTTTGAAGGGTAGTGGACACCAGGATCCTTTGGATTAATCCTCTGCCACCTCTCTC | + |
| A_24_P401990 | ERCC2 | excision repair cross-complementing rodent repair deficiency, complementation group 2 | TGCTGCATACTCTGGAGATCACCGACCTTGCTGACTTCTCCCCGCTCACCCTCCTTGCTA | + |
| A_24_P40306 | SERPIND1 | serpin peptidase inhibitor, clade D (heparin cofactor), member 1 | GCACAATAGCCCATGCTGTAAGCTCATAGAAGTCACTGTAACTGTAGTGTGTCTGCTGTT | + |
| A_24_P408772 | NAMPT | nicotinamide phosphoribosyltransferase | ACAGGCACCACTAATAATCAGACCTGATTCTGGAAACCCTCTTGACACTGTGTTAAAGGT | + |
| A_24_P414183 | OGG1 | 8-oxoguanine DNA glycosylase | GCGCTAAGGATGGTTTTATCTTCCCTTTATTACAAGAAGGAACAATAAAATAGAAACATT | + |
| A_24_P418816 | GPX7 | glutathione peroxidase 7 | CGTCTTGCCAACAAAAATGTGTGGCAAATAGAAGTATATCAAGCAATAATCTCCCACCCA | + |
| A_24_P48856 | CBS | cystathionine-beta-synthase | AGCCGTCAGACCAAGTTGGCAAAGTCATCTACAAGCAGTTCAAACAGATCCGCCTCACGG | + |
| A_24_P50759 | TNF | tumor necrosis factor | GGCTGAGGCCAAGCCCTGGTATGAGCCCATCTATCTGGGAGGGGTCTTCCAGCTGGAGAA | + |
| A_24_P53600 | ATRN | attractin | GAATTTGTGCACAAAACATTCTAAACACTAGTGAAGCCTGTTTCGTTGAACTAATTCTGG | + |
| A_24_P62883 | UCN | urocortin | CCAGGACCCGAGTCTGCGCTGGAGCCCCGGGGCACGGAACCAGGGTGGCGGGGCCCGCGC | + |
| A_24_P69538 | TLR4 | toll-like receptor 4 | ATGGTAAATCATGGAATCCAGAAGGAACAGTGGGTACAGGATGCAATTGGCAGGAAGCAA | + |
| A_24_P79054 | TGFB1 | transforming growth factor, beta 1 | CAACTATTGCTTCAGCTCCACGGAGAAGAACTGCTGCGTGCGGCAGCTGTACATTGACTT | + |
| A_24_P85158 | SIRT3 | sirtuin 3 | GTGGGGCTATTTTTAATGAGAGAAAATCTGTTCTTTCCAGCATGAAATACATTTAGTCTC | + |
| A_24_P910898 | MUTYH | mutY homolog (E. coli) | CTGTGATGGCCTGGCCAGGCAGCCGGAAGAGGTGGTATTGCAGGCCTCTGTCTCCTCATA | + |
| A_24_P912720 | CHRNA4 | cholinergic receptor, nicotinic, alpha 4 | GGCCCCCTTTCGCCCGCGTTCGATGGCCTGGGACCCCCAGAGCCAGAGGGAGGCAGGCTC | + |
| A_24_P913489 | LDLR | low density lipoprotein receptor | TGGCCACGAACATTTTGGTCACGAGATGGAGTCCAGGTGTCGTCCTCACTCCCTTGCTGA | + |
| A_24_P913947 | G6PD | glucose-6-phosphate dehydrogenase | CTTGCAGCTTGTCACTAGGAAGCCTTGTTTGGGGTCCCCATGCCCTTGAACCAGGTGAAC | + |
| A_24_P914134 | PRNP | prion protein | CTTCCCCGCCCCGCGTCCCTCCCCCTCGGCCCCGCGCGTCGCCTGTCCTCCGAGCCAGTC | + |
| A_24_P914519 | CYBB | cytochrome b-245, beta polypeptide | ACATTTTTCACCCAGACGAATTGTACGTGGGCAGACCGCAGAGAGTTTGCCTGTGCATAA | + |
| A_24_P919974 | REN | renin | AGAGGATCTTCCTCAAGAGAATGCCCTCAATCCGAGAAAGCCTGAAGGAACGAGGTGTGG | + |
| A_24_P923028 | TAT | tyrosine aminotransferase | TCTTTAGTGCTCTTATAACAGGACTAAATGTCTAGCAATCCTCTTCACTTTCCTGATGCA | + |
| A_24_P923684 | SIRT3 | sirtuin 3 | GGAATTGGTGACCTAGGAAAACTGTTGAATTCTAAAAAGAATGAAGTTAGTTTCTAACCC | + |
| A_24_P925191 | CAT | catalase | CTTGGGAATAGGAAGTAATACTGTATAAGACAAGACACTAACTTGTTATGCAGAAGGAAA | + |
| A_24_P929106 | PSIP1 | PC4 and SFRS1 interacting protein 1 | AATGGCCTTAGAAATACTATATAATCTGGTTGATTTTGGACTTGAAGTTTGGTGTCTGTC | + |
| A_24_P930276 | NFE2L1 | nuclear factor (erythroid-derived 2)-like 1 | TCCGAAAACCAACAAAATAGAACCGCGGTCCTATTCCATTATTCCTAGCTGCGGTATCCA | + |
| A_24_P930985 | RXRA | retinoid X receptor, alpha | GCCTAGAAAGAGGAAGTGCGTGAGTTCCTTTCCTTTGTGCTTCCGAGCAACCTGGACCTG | + |
| A_24_P935819 | SOD2 | superoxide dismutase 2, mitochondrial | GAAGATAATCGATAGTCATGTTTTTTAGACTCTCTGTATTGCTTGGTAAGCTACGTAGTA | + |
| A_24_P936319 | GAB1 | GRB2-associated binding protein 1 | GAAAGATACATGCATTCTATGGTAACAACTACTGTCAATAACATCTGATGTTACATGCAC | + |
| A_24_P940059 | IPCEF1 | interaction protein for cytohesin exchange factors 1 | CCCAGGGTTTTGTAATACATAATTGAAAATAAAAGTCCCTGAAACTAAATGTTTGCAGCC | + |
| A_24_P945165 | ACOT11 | acyl-CoA thioesterase 11 | GTATTAATTAATTTTGGACTTTGCTCACTAAAGTTTGAATGTTAAGAATAGATGTATACA | - |
| A_24_P98371 | PSIP1 | PC4 and SFRS1 interacting protein 1 | GAGAGAACCTGCATATATACCAGTCATTATCTGTTTGGTCCTTATACAGTTTTAACTTAC | + |
| A_32_P109653 | SNCA | synuclein, alpha (non A4 component of amyloid precursor) | TGTGGTTTGGTATTCCAAGTGGGGTCTTTTTCAGAATCTCTGCACTAGTGTGAGATGCAA | + |
| A_32_P138359 | MSRA | methionine sulfoxide reductase A | TGCGGCTCCGCTGCCGGTAGCGCCGTCCCCCGGGACCACCCTTCGGCTGGCGCCCTCCCA | + |
| A_32_P149416 | TXNRD1 | thioredoxin reductase 1 | CTCTAGCCATGACTTGCTTTTGGACAAAAATCAACTGCTAACGTTTTTCATCTCTAATAT | + |
| A_32_P151823 | OXR1 | NaN | AGGGCATGTCTTCTTATTCCATGTGACAGTGGCTGGCTGAGTTGCTAGTACGTTTTTGAA | + |
| A_32_P163469 | NFE2L1 | nuclear factor (erythroid-derived 2)-like 1 | AGAGAGAGAGATGCTGTTGAGCACATGACAAAATAAAATAAAATGGATGATTCATTCTTA | + |
| A_32_P167000 | APP | NaN | GGGCTATGTGATAAATAATCAGGAGAGAATCTATTCATGCACTAGTTTGATACAGCTAAA | + |
| A_32_P169754 | YBX1 | Y box binding protein 1 | GGTAAAATGGTTCAATGTAAGGAACGGATATGGTTTCATCCACAGGAACGACACCAAGGA | + |
| A_32_P171488 | DUSP1 | dual specificity phosphatase 1 | TTGACTCGATTAGTCCTCATAAGGTAAGCAAGGCAGATGGTGGCTGACCGGGAAATGCCT | + |
| A_32_P173177 | SELK | selenoprotein K pseudogene | ACTGTGTCTAATTTGCTACCAAATGTAATTATCAGGGTATTTATAAAACCATAGCTAACA | + |
| A_32_P177953 | GCLM | glutamate-cysteine ligase, modifier subunit | ATTTATTGTATGTTGGCTAGCAGTTCATCCTTCTGCAAAATATGCATTCAGAGAAATGTG | + |
| A_32_P180199 | NFKB1 | nuclear factor of kappa light polypeptide gene enhancer in B-cells 1 | AGCAGCAGCTGGCAAAGCTTAGTAAGCAACCTCATCCCCAGATGCATCCGCTCAGCCAGT | + |
| A_32_P180603 | BRCA1 | breast cancer 1, early onset | ACACTTTTTCTTCCTTCAGCAAGCAAAATTATTTATGAAGCTGTATGGTTTCAGCAACAG | + |
| A_32_P217709 | RAC1 | ras-related C3 botulinum toxin substrate 1 (rho family, small GTP binding protein Rac1) | GTTACACAACCAATGCATTTCCTGGAGAATATATCCCTACTGTCTTTGACAATTATTCTG | + |
| A_32_P218989 | YBX1 | Y box binding protein 1 | TAAATAGAACTATCTGCATTATCTATGCAGCATGGGATTTTTATTATTTTTACCTAAAGA | + |
| A_32_P22702 | PSIP1 | PC4 and SFRS1 interacting protein 1 | AGATTCCGTGATCACCCAAGTGCTGAATAAATCTCTTGCTGAACAAAGACAGCATGAGGA | + |
| A_32_P227525 | PRDX2 | peroxiredoxin 2 | ATGACTGAAAGCTGCGTGGGCAAAGGCTAGACGCACGGACGATCACACGCGTGGACCCGC | + |
| A_32_P25419 | SEPP1 | selenoprotein P, plasma, 1 | TAATGGCTTCTTCTACATATGGGAAAGTTAGGAAGGAAAAAGGCAAACCAAGATGATATA | + |
| A_32_P32043 | PSIP1 | NaN | AGTACAGCGTTTTACTCTGCTTTCTTAGTTTGGAGGATATACAAGTAGTTTTAGCTCCTT | + |
| A_32_P35906 | MMP2 | matrix metallopeptidase 2 (gelatinase A, 72kDa gelatinase, 72kDa type IV collagenase) | TGGGAGGAGTACAGTCAGCATCTATTCTTGGGCACCGGGAGGAGCCACTCTCTGGAATCT | + |
| A_32_P45009 | IDH1 | isocitrate dehydrogenase 1 (NADP+), soluble | GTTCTGGTGTCATAGATGTCCCATTTTGTGAGGTAGAGCTGTGCATTAAACTTGCACATG | + |
| A_32_P60687 | MYC | v-myc myelocytomatosis viral oncogene homolog (avian) | TCCGTAGCTGTTCAAGTTTGTGTTTCAACTGTTCTCGTCGTTTCCGCAACAAGTCCTCTT | + |
| A_32_P62434 | PPP1R15B | protein phosphatase 1, regulatory subunit 15B | ATGGGTTTTGTGGCAGTCCTTGGAAATATCCTAGGTAGAACTTAATGTAGAAATAAAAAG | + |
| A_32_P66881 | TLR4 | toll-like receptor 4 | TTACTGAGTGTTTCAGAGTGTGTTTGGTTTGAGCAGGTCTAGGGTGATTGAACATCCCTG | + |
| A_32_P74983 | MICB | MHC class I polypeptide-related sequence B | CTGCCTGGATCTCACCAGCACTTTCCCTCTGTTTCCTGACCTATGAAACAGAGAAAATAA | + |
| A_32_P78121 | SOD2 | superoxide dismutase 2, mitochondrial | AGTGGAATAAGGCCTGTTGTTCCTTGCAGTGGATCCTGATTTGGACAAGCAGCAATTTGT | + |
| A_32_P79396 | NAMPT | nicotinamide phosphoribosyltransferase | AGGGCCGATTATCTTTACATAGGACGCCAGCAGGGAATTTTGTTACACTGGAAGAAGGAA | + |
| A_32_P82448 | PDLIM1 | NaN | TGACACTGAACAAAACAGTTTTCCTTTAATTGTAAAAGCGGGCATCGCACAGCTGGTGTG | + |

**Quality control:** +: Quality control passed for given sequence, -: Quality control not passed for given sequence

**Embryonal growth:** Number of sequences Agilent array = 294 (160 unique genes); Number of sequences Agilent array after quality control filtering = 289 (158 unique genes)

**DNA methylation:** Number of sequences Agilent array = 102 (49 unique genes); Number of sequences Agilent array after quality control filtering = 101 (48 unique genes)

**Oxidative stress:** Number of sequences Agilent array = 275 (154 unique genes); Number of sequences Agilent array after quality control filtering = 269 (152 unique genes)
